# Targeting the Cell Cycle, RRM2 and NF-κB for the Treatment of Breast Cancers

**DOI:** 10.3390/cancers16050975

**Published:** 2024-02-28

**Authors:** Nahid Sultana, Howard L. Elford, Jesika S. Faridi

**Affiliations:** 1 Department of Physiology and Pharmacology, Thomas J. Long School of Pharmacy, University of the Pacific, Stockton, CA 95211, USA; n_sultana@u.pacific.edu; 2 Molecules for Health, Inc., Richmond, VA 23219, USA; mol4hlth@yahoo.com

**Keywords:** palbociclib, RRM2, NF-κB, cell cycle

## Abstract

**Simple Summary:**

Drug inhibitors of the cell cycle are utilized for treating hormone responsive breast cancer. Yet, over time, drugs such as palbociclib can become ineffective. Our study examines the effectiveness of adding a ribonucleotide reductase inhibitor to palbociclib therapy. We observed changes in cell cycle and other proteins, as well as the inhibition of tumor growth. Ultimately, our data suggest a strategy to target RRM2, NF-κB and cyclin D1 to supplement traditional therapies as well as offering improved efficacy for breast cancer treatment.

**Abstract:**

A hallmark of cancer is the dysregulation of the cell cycle. The CDK4/6 inhibitor palbociclib is approved for treating advanced estrogen-receptor-positive breast cancer, but its success is limited by the development of acquired resistance owing to long-term therapy despite promising clinical outcomes. This situation necessitates the development of potential combination strategies. Here, we report that didox, an inhibitor of ribonucleotide reductase in combination with palbociclib, can overcome palbociclib resistance in ER-positive and ER-negative breast cancers. This study shows didox downregulates an element of the cell cycle checkpoint, cyclin D1, accompanied by a reduction in NF-κB activity in vitro and tumor growth inhibition of palbociclib-resistant ER positive breast cancer tumor growth in vivo. Furthermore, didox induces cell cycle arrest at G1 as well as reduces ROS generated by on-target effects of palbociclib on the cell cycle. Our current study also reports that the CCND1 and RRM2 upregulation associated with palbociclib-resistant breast cancers decreases upon ribonucleotide reductase inhibition. Our data present a novel and promising biomarker-driven combination therapeutic approach for the treatment of ER-positive and ER-negative breast cancers that involves the inhibition of the CDK4/6-cyclinD1/pRb cell cycle axis that merits further clinical investigation in human models.

## 1. Introduction

The cell cycle is a series of regulated events orchestrated by specific enzymes and proteins. Cyclins and cyclin-dependent kinases (CDKs) are the key regulatory machinery known as cell cycle checkpoints that can speed, slow, or even halt the process. CDKs form a complex with cyclins during each phase of the cell cycle. During G1, CDK4/6 binds with cyclin D to form an active kinase complex, which phosphorylates the retinoblastoma protein 1 (Rb1) to counteract its inhibitory effect on the transcription factor E2F causing G1-S phase shift [1,2]. Uncontrolled cell growth owing to an abnormal cell cycle is one of the hallmarks of cancer [3]. In cancer cells, the CDK4/6–cyclin D1-Rb1-E2F axis is regarded as the most dysregulated cell cycle pathway [1].

Endocrine treatment is an effective first-line therapy for targeting ER+, HER2− breast cancers. Yet, its success is limited by the development of acquired resistance due to long-term therapy. Cyclin D and CDK4/6 complex-mediated phosphorylation of the retinoblastoma (Rb) tumor suppressor protein and subsequent inactivation drive the cell cycle from G1 into the S phase for DNA synthesis. This observation led to the development of the first selective CDK4/6 inhibitor, palbociclib, which induces cell cycle arrest at the G1 phase in cancer cells [4]. Palbociclib is a specific CDK4/6 inhibitor, which decreases the phosphorylation of Rb and subsequently inhibits cancer cell growth [5]. Additional efficacy was observed when palbociclib was given in combination with the selective estrogen receptor degrader (SERD) fulvestrant or the aromatase inhibitor (AI) letrozole [6]. Unfortunately, despite the success rate of cell cycle checkpoint CDK4/6 inhibitors, 10% of breast cancer patients have intrinsic resistance, and many later present with acquired resistance since the median response rate among patients receiving first-line treatment of CDK4/6 inhibitors plus hormone therapy was determined to be only 24–28 months in the PALOMA, MONALEESA, and MONARCH clinical trials [7].

The deregulation of cell signaling molecules and activation of several escape pathways could be the mechanisms behind the progression of resistance in breast cancer. This paves the pathway for designing novel therapeutic strategies to improve patient outcomes [8]. Here, we focus on circumventing palbociclib resistance by targeting estrogen-receptor-positive (ER+) and estrogen-receptor-negative (ER−) breast cancers with a unique ribonucleotide reductase subtype M2 (RRM2) enzyme inhibitor.

Tumor growth is dependent on the activity of ribonucleotide reductase (RR), which is the rate-limiting enzyme to catalyze the conversion of ribonucleotides to deoxyribonucleotides during DNA synthesis [9]. RR is a tetramer composed of two dissimilar subunits, RRM1 and RRM2. While RRM1 contains allosteric regulatory sites for the maintenance and balancing of deoxyribonucleotide triphosphate pools, RRM2 contains a binuclear iron center and a tyrosyl free radical for the enzymatic conversion of ribonucleotides to deoxyribonucleotides. The cell cycle regulates mammalian RR, whereby the RRM2 subunit is made in the late G1 prior to DNA replication, and it disappears in the late S or early G2 phase [10]. Overexpression of RRM2 is associated with higher proliferation and invasiveness of malignant cancers [11]. In previous studies, we have shown that RRM2 is upregulated in ER− as well as tamoxifen-resistant ER+ breast cancers [12,13,14]. Others have suggested that RRM2 may be a potential prognostic indicator for breast cancer treatment [15,16,17,18,19,20].

The free radical scavenger didox (3,4-dihydroxybenzohydroxamic acid), upon introduction to RRM2, chelates its iron (III) from the radical center, impeding the conversion of ribonucleotides to deoxyribonucleotides [21]. In addition to RR inhibition, didox also possesses synthetic antioxidant properties. This drug was originally developed as an antineoplastic and antiproliferative agent improving upon the activities of hydroxyurea [21,22]. It also improves the efficacy of DNA-targeting agents when used in combination [14,22,23].

The NF-κB transcription factor functions as a nuclear factor which binds to the enhancer element of the immunoglobulin kappa light-chain of activated B cells (IkB) [24]. Constitutive activation of NF-κB signaling can lead to various disorders as it regulates more than 500 genes responsible for proliferation, invasion, angiogenesis, metastasis, cellular transformation, and inflammation [25,26]. Upregulation of NF-κB in breast cancer primarily leads to increased cell proliferation, survival, metastasis and resistance to radiotherapy and chemotherapy [27,28]. Cell cycle progression and uncontrolled cell proliferation through upregulation of the expression of the cell cycle proteins cyclin D1 and CDKs with NF-κB activation have been reported in several studies [29,30].

In this study, we hypothesize that through the inhibition of RRM2, cyclin D1 and the NF-κB pathway, DDX can significantly halt the growth of ER+ and ER− breast cancer cells along with their PLB-resistant counterparts. We also want to delineate whether this combination therapy has the potential to arrest the cell cycle at G1 and re-sensitize PLB-resistant cells to CDK4/6 inhibition therapy. Our data present a new approach for the treatment of ER+ and possibly ER− breast cancers.

## 2. Materials and Methods

### 2.1. Cell Culture and Drug Treatment

MCF7 and MDA-MB-468 cells were purchased from ATCC every 6 months. The cells were maintained in advanced DMEM/F12 (Thermo Fisher Scientific, Waltham, MA, USA) supplemented with 5% FBS, 1% L-glutamine and 1% streptomycin and penicillin. Didox (DDX) was synthesized and kindly provided by Dr. Howard L. Elford, Molecules for Health, Inc. (Richmond, VA, USA). Palbociclib (PLB) was purchased from Sigma Aldrich (Burlington, MA, USA). Didox was dissolved in 0.9% sterile saline solution and palbociclib was dissolved in 0.1M HCl. All compounds were filtered through a 0.2 μM syringe filter and stored at −10 °C in the dark for a maximum of 1 week. The cells were treated with DDX, PLB, or a combination of DDX and PLB in phenol red free, serum free DMEM/F12 media (Thermo Fisher Scientific).

### 2.2. Establishment of the Palbociclib-Resistant Cell Lines

Palbociclib-drug-resistant cells were developed by culturing these in the media mentioned above containing increasing doses of palbociclib 0.1–4 μmol/L. The cells were subcultured at almost 95% confluency with an increment of 25% drug concentration. The resistant cells were established after 6 months and maintained in 1 μmol/L palbociclib.

### 2.3. IC_50_ Determination

IC_50_ values were normalized to those of their no-treatment controls and analyzed in GraphPad Prism by non-linear regression to obtain the IC_50_ values.

### 2.4. Western Blot Analysis

The cells were lysed with the cell lysis buffer (Cell Signaling Technology, Danvers, MA, USA) and lysates were clarified by centrifugation for 10 min at 14,000 rpm. After protein quantification with BCA assay, 50 µg of each sample was resolved by SDS-polyacrylamide gel electrophoresis and transferred to nitrocellulose membranes. Blots were blocked with blocking buffer (LI-COR Biosciences, Lincoln, NE, USA) for 1 h at room temperature, incubated with primary antibody (1:1000 dilution) overnight at 4 °C, followed by incubation with secondary goat anti-rabbit or goat anti-mouse IgG antibody (1:10,000 dilution) conjugated with IR Dye 800CW or 680RD (LI-COR Biosciences) at room temperature for 60 min. Membranes were subsequently washed, visualized, and quantified using Li-COR Odyssey Fc and CLX imaging system and image studio software. The following antibodies were used: RRM2 (Sigma Aldrich), Cell Cycle Regulation Antibody Sampler Kit II, NF-κB pathway sampler kit, NF-κB Family Member Antibody Sampler Kit, Phospho-p53 Antibody Sampler Kit, Rb Antibody Sampler Kit, Akt Isoform Antibody Sampler Kit, Phospho-Akt Pathway Antibody Sampler Kit, and GAPDH (Cell Signaling Technology).

### 2.5. Real Time-Quantitative Polymeric Chain Reaction (RT-qPCR) Analysis

Total RNA was isolated using RNeasy (QIAGEN, Germantown, MD, USA) according to the manufacturer’s protocol. Briefly, 1 µg RNA was converted to cDNA using the RevertAid first strand cDNA synthesis kit (Thermo Fisher Scientific). RT-qPCR reactions were performed using human predesigned CCND1 (1:20) and Tp53 (1:20) probe-based primers (Integrated DNA Technology, Coralville, IA, USA). Fold change was determined using the comparative 2^−ΔΔCt^ method and RPL13A housekeeping gene (Integrated DNA Technology).

### 2.6. Cell Cycle Analysis

The cells were grown in 100 mm cell culture dishes and incubated with drugs for 24 h. The cells were then harvested (1 × 10^6^ cells) with trypsin, washed with HBSS, resuspended in 200 μL PBS, fixed in 1 mL ice cold 70% ethanol, and stored overnight at −20 °C. Afterwards, the fixed cells were strained, centrifuged, and washed with 0.25 mL 1% PBS, resuspended with 200 μL cell cycle reagent (Luminex, Austin, TX, USA), and incubated in the dark for 30 min at room temperature. Cell cycle phase analysis was carried out using the Guava Muse flow cytometer and cell analyzer software with cell cycle kit (MCH100106) from Luminex according to the manufacturer’s protocol.

### 2.7. Cellular ROS Measurement

Cellular ROS levels were measured by using DCFDA/H2DCFDA—cellular ROS assay kit (ab113851, Abcam, Waltham, MA, USA) according to the manufacturer’s protocol. Briefly, the cells were plated (15,000 cells/well) in a 96-well plate in advanced DMEM/F12 media supplemented with 5% FBS, 1% L-glutamine and 1% streptomycin and penicillin with no treatment, 1 mM palbociclib, 30 mM didox or combination of 1 mM palbociclib and 30 mM didox. After 48 and 72 h, 40 mM DCFDA reagent was added, and fluorescence was measured after incubating for 1 hour at 37 °C.

### 2.8. NF-κB Activity Assay

A total of 40,000 cells were seeded in a 96-well plate and incubated overnight. The next day, NF-κB (CSS-013L Cignal reporter assay kit, QIAGEN) was transfected using Fugene HD transfection reagent (Promega, Madison, WI, USA) according to the manufacturer’s protocol. After 24 h, the cells were treated with DDX 30–900 μmol/L and luminescence was measured using Dual luciferase reagent (Promega).

### 2.9. Xenograft Studies

All animal procedures were approved by the Institutional Animal Care and Use Committee of the University of the Pacific (Protocol 22R04). MCF7 PR breast cancer cells were injected subcutaneously into the flank of nude female mice. Upon an average tumor volume of 100 mm^3^, mice were randomized into groups and treatments were initiated. As indicated, the mice received PLB (intraperitoneal, 10–25 mg/kg/day, *n* = 4), DDX (intraperitoneal, 425 mg/kg/day, *n* = 5), a combination of both (*n* = 2), or NT (*n* = 3). For both injections, the solutions were prepared daily and injected fresh. Body weights were measured every 3 days and tumor volumes were measured using the ellipsoid formula of [4/3p(r1)2(r2)], where r1 < r2.

### 2.10. Statistical Analysis

The data were analyzed using unpaired two-sample *t*-test of GraphPad Prism software Version 9.4. The results are presented as mean ± SEM. *p*-value < 0.05 was considered statistically significant.

## 3. Results

### 3.1. Resistance to Palbociclib (PLB) Alters Expression of Proteins Involved in Cell Growth and Cell Cycle Regulatory Pathways

**Figure 1:**
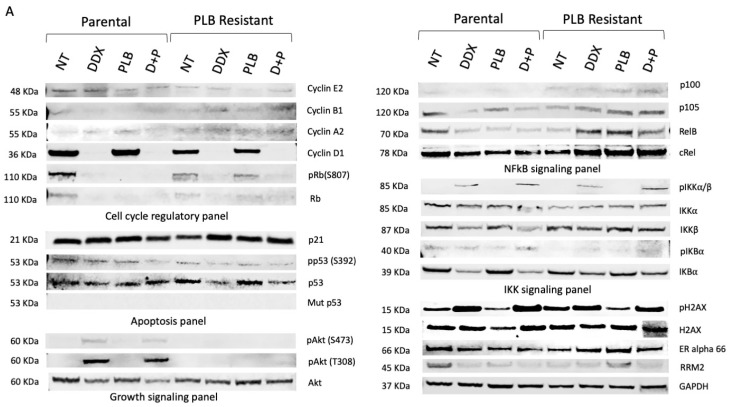

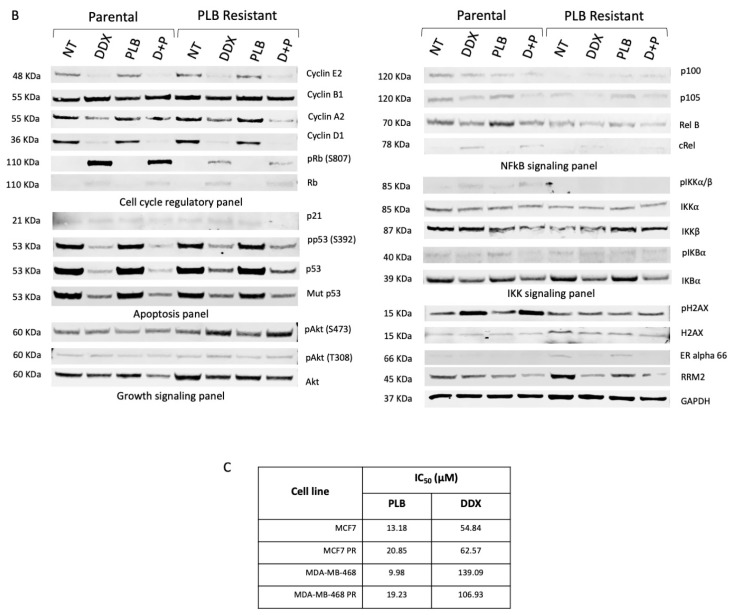
Resistance to palbociclib alters expression of proteins involved in cell growth and cell cycle regulatory pathways. Western blot analysis of cellular and apoptotic proteins of MCF7 and MCF7 PR (**A**), Western blot analysis of MDA-MB-468 and MDA-MB-468 PR (**B**) cells treated with vehicle (NT), 600 µmol/L DDX, 1 µmol/L PLB, and combination of 600 µmol/L DDX + 1 µmol/L PLB. IC_50_ values of drug palbociclib and didox in all cell lines (**C**). The uncropped blots are shown in Supplementary Materials.

Here, we seek to evaluate the effect of palbociclib resistance on cell cycle, apoptosis, growth signaling, NF-κB and IKK signaling in Rb-sensitive (MCF7) and Rb-deficient (MDA-MB-468) parental and palbociclib (PLB)-resistant breast cancer cells. With palbociclib resistance, we observed that pRb (S807), Rb, cyclin D1, p21, p105, RelB, IKKα, ERα and RRM2 levels are downregulated in untreated MCF7 cells (Rb-sensitive). Interestingly, upon palbociclib treatment, we observed that resistant cells expressed elevated p21, p100, p105, RelB, ERα and RRM2 levels when compared to the NT group of MCF7 PR cells. Yet, the pRb (S807) levels remained unchanged with PLB treatment in MCF7 PR cells (Figure 1A).

Next, we examined the inhibition of ribonucleotide reductase on our parental and resistant breast cancer cells by using the ribonucleotide reductase inhibitor didox alone or in combination with palbociclib. Cell cycle regulatory proteins cyclin D1, Rb and pRb S807 along with IKK and NF-κB signaling proteins p100, p105, RelB, cRel and IKKβ were downregulated with DDX and combination treatment. Yet, pIKKα/β was significantly increased with DDX combination in both parental and palbociclib-resistant MCF7 cells. DDX alone or in combination with PLB also caused an increase in the DNA damage signaling protein pH2AX, indicating DNA damage due to PLB (Figure 1A).

Although, MDA-MB-468 ER− cells are Rb deficient, here, we have found that DDX treatment increased the Rb levels again in MDA-MB-468 parental and palbociclib-resistant breast cancer cells. We also found upregulation of cyclin D1, AKT and RRM2 with palbociclib resistance in MDA-MB-468 breast cancer cells compared to parental cells. Upon DDX treatment, we observed downregulation of the cell cycle regulatory proteins cyclin D1, cyclin A2, and cyclin E2 along with the NF-κB signaling proteins p100, p105, and RelB and the apoptotic proteins p53, phospho-Tp53 (S392), and mutant p53 in MDA-MB-468 cells along with its palbociclib-resistant counterpart (Figure 1B). DDX increased the DNA damage signaling protein pH2AX expression in MDA-MB-468 parental PLB-resistant cells (Figure 1B). DDX did not reduce wild-type p53 in MCF7 parental or PLB-resistant cells (Figure 1A). Yet, mutant p53 and phospho-p53 S392 decreased in the DDX- and DDX+PLB-treated groups in both parental and PLB-resistant MDA-MB-468 cell (Figure 1B). Upregulation of cyclin D1, AKT and RRM2 was only observed in the MDA-MB-468 PLB-resistant NT group but not in parental MDA-MB-468 cells (Figure 1B). The IC_50_ values of PLB drug were higher for PLB-resistant breast cancer cells compared to parental cells (Figure 1C).

### 3.2. Inhibition of Ribonucleotide Reductase Alters Cell Cycle Regulatory and NF-κB Pathway Expression in a Dose-Dependent Manner

**Figure 2:**
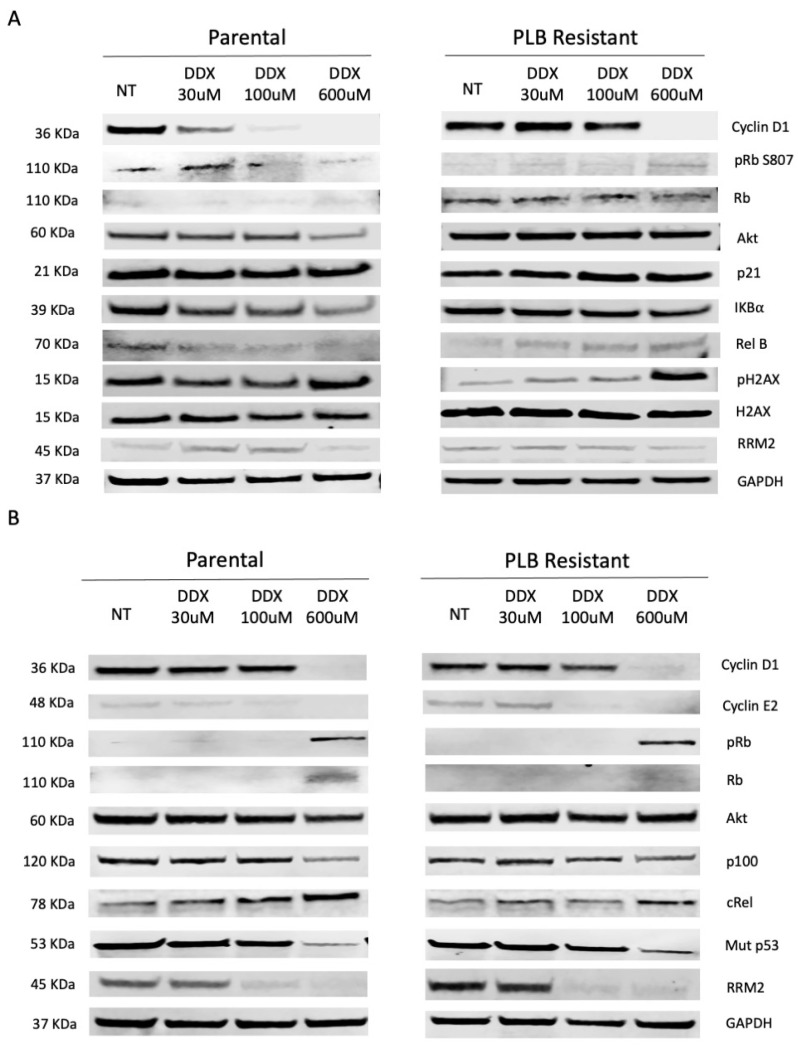
DDX inhibits cell cycle regulatory proteins and RRM2 in a dose-dependent manner in both parental and PLB-resistant ER+ and ER− breast cancer cell lines. Western blot analysis of cellular and apoptotic proteins in MCF7 (**A**) and MDA-MB-468 (**B**) cells treated with vehicle (NT), 30 µmol/L DDX, 100 µmol/L DDX, and 600 µmol/L DDX for 24 h. The uncropped blots are shown in Supplementary Materials.

Based on the proliferation assays, we determined to use the ER+ MCF7 (Rb active) and the ER− MDA-MB-468 (Rb-deficient) parental and PLB-resistant breast cancer cell lines. To determine the optimal concentration of DDX, we conducted dose-dependent Western blot analysis on cell cycle regulatory, apoptotic and NF-κB pathways using DDX concentrations varying between 30 and 600 μmol/L in MCF7 and MDA-MB-468 parental and PLB-resistant cells. DDX reduced the expression of cell cycle regulatory proteins cyclin D1, phosphorylated Rb S807 and NF-κB regulatory RelB and IKBα but increased pH2AX level in MCF7 parental and PLB-resistant cell lines (Figure 2A). Interestingly, DDX also reduced cyclin D1, cyclin E2, NF-κB regulated p100 and mutant p53 while increasing Rb, pRb S807 and cRel expression in MDA-MB-468 parental and PLB-resistant cell lines (Figure 2B). We observed the highest number of alterations in the mentioned proteins at the highest DDX dose in both MCF7 and MDA-MB-468 parental and PLB-resistant cell lines.

### 3.3. Inhibition of Ribonucleotide Reductase Causes Cell Cycle Arrest at G1 Phase and ROS Reduction

**Figure 3:**
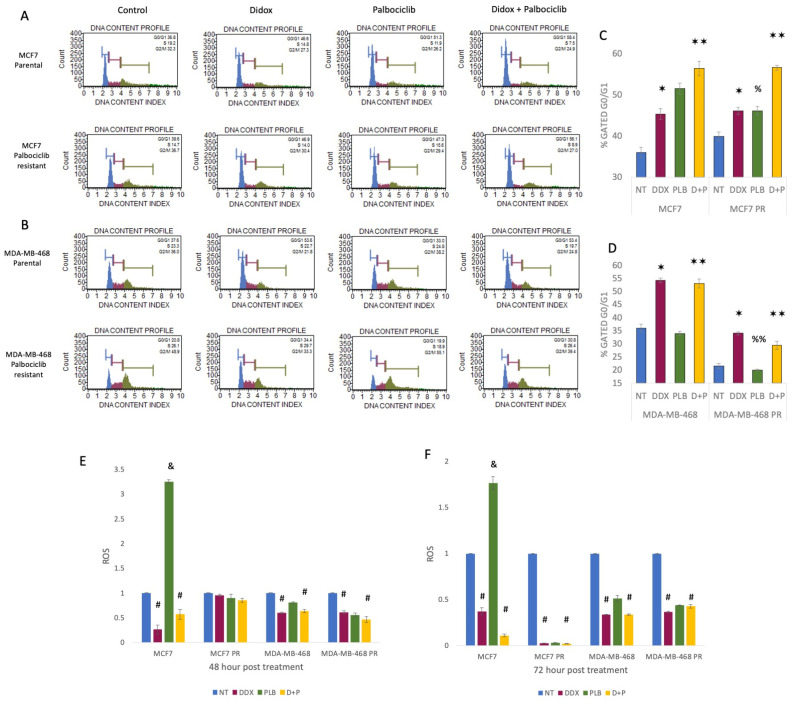
DDX alters the cell cycle and causes cell cycle arrest in MCF7 and MDA-MB-468 parental and palbociclib-resistant breast cancer cells at G1 and reduces ROS. Cell cycle analysis by flow cytometry of MCF7 and MCF7 PR (**A**), MDA-MB-468 and MDA-MB-468 PR (**B**) cells treated with NT, DDX 100 µmol/L, PLB 1 µmol/L and combination of DDX and PLB for 24 h. Difference between percent gated at G0/G1 in MCF7 and MCF7 PR (**C**), MDA-MB-468 and MDA-MB-468 PR (**D**) with same treatment conditions as before. DDX scavenges free radicals in MCF7 and MDA-MB-468 breast cancer cells and their palbociclib-resistant counterparts (**E**,**F**). Data are presented as mean ± SEM, *n* = 3. ∗ *p* < 0.009, ∗∗ *p* < 0.01; significant difference between NT and DDX-, NT and D+P-treated breast cancer cells. % *p* < 0.02, %% *p* < 0.0002; significant difference between MCF7 and MCF7 PR, MDA-MB-468 and MDA-MB-468 PR breast cancer cells treated with PLB. & *p* < 0.0001; significant difference between NT and PLB. # *p* < 0.001; significant difference between NT and DDX or D+P.

To further understand whether palbociclib resistance may lead to cell cycle changes, we used flow cytometry, and evaluated the effect of DDX alone and in combination with palbociclib on the cell cycle profile of breast cancer cell lines. We report that the development of palbociclib resistance in ER+ MCF7 and ER− MDA-MB-468 breast cancer cells yielded a shift in their cell cycle profile (Figure 3A,B). As palbociclib treatment is known to induce G1 cell cycle arrest in Rb-sensitive cells, we observed a significant increase in the percentage of cells in G1 in parental MCF7 (Rb-sensitive) but not MDA-MDB-468 (Rb-deficient) breast cancer cells (Figure 3A,B). However, MCF7 palbociclib-resistant cells exhibited a reduced G1 cell cycle arrest as compared to parental MCF7 cells (Figure 3C). Interestingly, MDA-MB-468 PR cells exhibited a lower percentage of G1 cells both untreated and with PLB treatment (Figure 3D).

Next, to determine whether RR inhibition would have an additive effect on G1 arrest, the cells were treated with DDX alone or in combination with PLB. Upon treatment with DDX, we observed an increase in the percentage of G1 cells from 36.8% (NT) to 46.6% (DDX alone) to 58.4% (DDX+PLB) in MCF7 cells. Similarly, in the MCF7 palbociclib-resistant counterpart, the cell cycle shift to the G1 phase was 46.9% with DDX and 56.1% with combination therapy as compared with 39.6% of NT (Figure 3A,B). Additionally, we also observed significantly higher G1 cell cycle arrest in MDA-MB-468 and MDA-MB-468 PR cells treated with DDX alone and with the combination of DDX and PLB compared to no treatment. The G1 cell cycle shifted to 53.6% with DDX and 53.4% with combination therapy from 37.6% with NT in MDA-MB-468 cells. On the other hand, palbociclib-resistant MDA-MB-468 cells had a G1 phase shift from 20.8% with NT to 34.4% with DDX alone and to 30.8% with combination treatment (Figure 3C,D). There appears to be no additive benefit of PLB treatment on G1 arrest in the parental or palbociclib-resistant Rb-deficient MDA-MB-468 cells.

Along with cell cycle arrest, oxidative stress is another stress response signal that is induced by reactive oxygen species (ROS), which is known to initiate cancer angiogenesis, metastasis, and survival at different concentrations. The inhibition of CDK4/6 by on-target effects of palbociclib (1 µM) induced ROS in MCF7 cells, which was reduced by the ribonucleotide reductase inhibitor DDX. Moreover, DDX shows an ROS-reducing effect on MDA-MB-468 cells as well as their palbociclib-resistant counterparts (Figure 3E,F).

### 3.4. Inhibition of Ribonucleotide Reductase Reduces ER+ Palbociclib-Resistant Tumor Growth and Decreases NF-κB Activation, Whereas Palbociclib Resistance Increases CCND1 and RRM2 Expression

**Figure 4:**
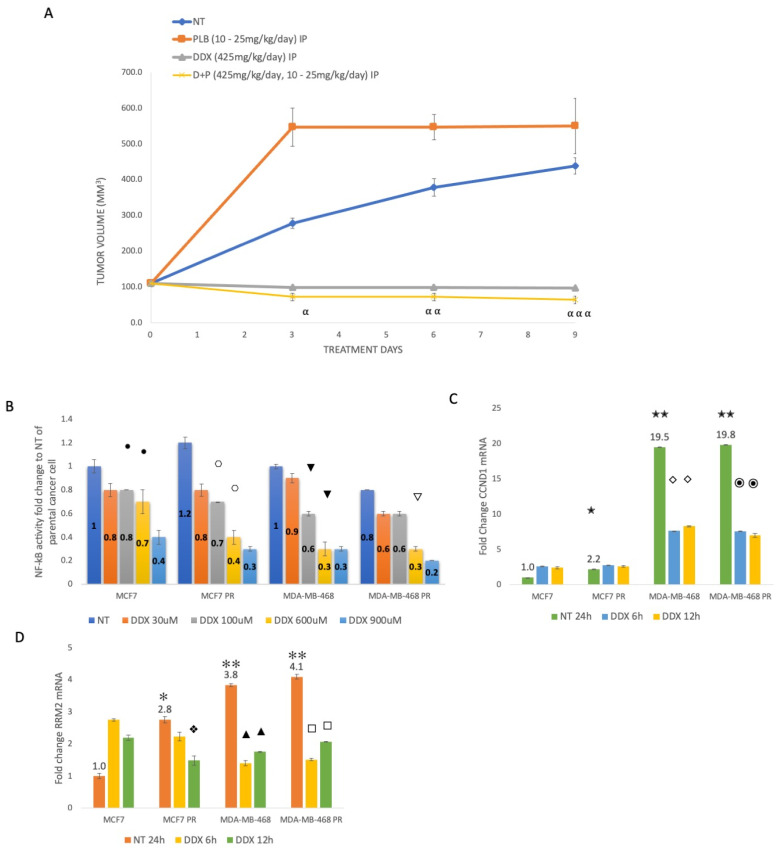
Inhibition of ribonucleotide reductase reduces ER+ palbociclib-resistant tumor growth and decreases NF-κB activation, whereas palbociclib resistance increases CCND1 and RRM2 expression. Mice bearing MCF7 PR tumors displayed reduced tumor growth with D+P treatment when compared with PLB alone, DDX alone or NT (**A**). Promoter analysis of NF-κB in all breast cancer cells treated with DDX at a dose ranging from 30 µmol/L to 1 mmol/L for 6 h (**B**). RT-qPCR of CCND1 (**C**) and RRM2 (**D**) mRNA fold changes with didox 100 µmol/L for 6 h and 12 h compared to NT in ER+ and ER− breast cancer and their PLB-resistant counterparts. All experiments were carried out in triplicate. Comparisons between groups were made by two-sample *t* tests. ⍺ *p* < 0.012, MCF7 PR PLB day 3 vs. MCF7 PR D+P day 3. ⍺⍺ *p* < 0.0004, MCF7 PR PLB day 5 vs. MCF7 PR D+P day 5. ⍺⍺⍺ *p* < 0.0043, MCF7 PR PLB day 7 vs. MCF7 PR D+P day 7. • *p* < 0.05, MCF7 NT vs. DDX treatment 100 mmol/L and 600 mmol/L. ⎔ *p* < 0.003; significant difference between MCF7 PR NT and DDX treatment 100 mmol/L and 600 mmol/L, ▼ *p* < 0.003, MDA-MB-468 NT and DDX treatment 100 mmol/L and 600 mmol/L, ▽ *p* < 0.0007 MDA-MB-468 PR NT and DDX treatment 600 mmol/L. ✭ *p* = 0.002; significant difference between MCF7 and MCF7 PR breast cancer cell lines. ✭✭ *p* = 0.0001; significant difference between MCF7 and MDA-MB-468 and MDA-MB-468 PR breast cancer cell lines. ✻ *p* < 0.01 and ✻✻ *p* < 0.0003; significant difference between MCF7 and MCF7 PR, MDA-MB-468, MDA-MB-468 PR breast cancer cells. ◇ *p* < 0.0003 and ⦿ *p* < 0.002; significant difference between MDA-MD-468 NT and DDX treatment 6 h and 12 h, MDA-MB-468 PR NT and DDX treatment 6 h and 12 h. ❖ *p* < 0.03, MCF7 PR NT and DDX treatment 12 h. ▲ *p* < 0.0003; significant difference between MDA-MD-468 NT and DDX treatment 6 h and 12 h, □ *p* < 0.0002, MDA-MB-468 PR NT and DDX treatment 6 h and 12 h.

Nude mice were injected with MCF7 palbociclib-resistant cells to determine the benefit of adding DDX treatment in vivo. Treatment began when tumors were approximately 110 mm^3^. After nine days of treatment, the no-treatment group reached a tumor average volume of 438 mm^3^, whereas the PLB-treated mice had a tumor volume average of 549.2 mm^3^. Animals treated with DDX and combination therapy DDX+PLB developed significantly smaller tumors, with average tumor volumes of 96.8 mm^3^ and 63.9 mm^3^, respectively (Figure 4A).

To examine whether NF-κB is activated in PLB-resistant cells, we performed an NF-κB activity assay. When comparing MCF7 untreated cells to MDA-MB-468 cells, we observed 24-fold greater NF-κB activity. We also observed a small increase in palbociclib resistance in MCF7 cells but not in MDA-MB-468 cells (Figure 4B). RRM2 inhibition reduced NF-κB activity by 60–80% in both ER+ and ER− cells, as well as in their palbociclib-resistant counterparts, in a dose-dependent manner (Figure 4B).

To determine whether palbociclib resistance leads to transcriptional changes, we assessed the mRNA expression of CCND1 and RRM2 in ER+ and ER− parental and PLB-resistant breast cancers. We observed more than a 2.2-fold increase in CCND1 and 2.8-fold increase in RRM2 expression in PLB-resistant as compared to parental ER+ MCF7 breast cancer cells (Figure 4C,D). ER− MDA-MB-468 breast cancer cells exhibited an approximately 19-fold higher expression of CCND1 and 4-fold greater expression of RRM2 as compared to MCF7 cells (Figure 4C,D). As MDA-MB-468 breast cancer cells already have higher levels of CCND1 and RRM2 expression, we did not observe additional increased expression in these genes with PLB resistance (Figure 4C,D). To determine the effect of inhibiting RR on expression, the cells were treated with DDX for six and twelve hours, and the expression of RRM2 was downregulated in a time-dependent manner in MCF7 PLB-resistant, MDA-MB-468 and MDA-MB-468 PR cells (Figure 4D). Reduced CCND1 expression was also observed in ER− cells with DDX treatment at both six and twelve hours (Figure 4C).

## 4. Discussion

The development of acquired and de novo palbociclib resistance in breast cancers remains a management challenge in clinical oncology for the treatment of hormone-receptor-positive and HER2-negative breast cancer. Limited data exist regarding the role of CDK4/6 and cyclin D1 interaction in the development of palbociclib resistance, and effective targets for palbociclib-resistant ER+ and ER− breast cancers are largely unknown. To date, there are no studies examining the feasibility of a combination of CDK4/6 inhibitor palbociclib with an RRM2 inhibitor for treating both ER+ and ER− palbociclib-resistant breast cancers. In addition, this combination strategy might have the possibility of avoiding the development of palbociclib resistance.

Multiple studies have reported a direct link between the overexpression of RRM2 in breast cancers and increased cell proliferation and invasiveness, as well as drug and chemotherapy resistance [12,13,29,31]. Previously, we found that RRM2 is upregulated in ER− and drug-resistant breast cancer cells, rationalizing our hypothesis of targeting ribonucleotide reductase for ER+ as well as ER− breast cancer treatment [12,13,14]. Our current study shows increased expression of CCND1 and RRM2 mRNA with palbociclib resistance in ER+ MCF7 and ER− MDA-MB-468 cells compared to each parental cell line (Figure 4C,D). We also observed an upregulation of cyclin D1 and RRM2 protein levels in ER− palbociclib-resistant breast cancer cells, which suggests a possible mechanism for the development of palbociclib resistance (Figure 1B). The inhibition of ribonucleotide reductase by DDX alone or in combination with palbociclib decreases RRM2 and cyclin D1 and pRb levels in MCF7 ER+ breast cancer cells. We also observed a strong inhibition of ribonucleotide reductase on ER+ palbociclib-resistant tumor growth using DDX or in combination with palbociclib (Figure 4A). Although the exact mechanism underlying palbociclib resistance is largely unknown, this study demonstrates that the inhibition of RRM2 by DDX circumvents the emergence of palbociclib resistance.

NF-κB signaling has a unique function in breast cancer as it regulates a myriad of key regulatory genes necessary for cell proliferation, invasion, metastasis, and angiogenesis. Some studies have reported that NF-κB suppression can re-sensitize resistant breast cancer cells to treatment [32,33,34]. We have demonstrated that DDX alters the expression of various NF-κB proteins, including p105 and p100, halting the aid of these proteins in cancer cell survival and proliferation. DDX alone or in combination with PLB significantly decreased the expression of RelB, cRel, IKKβ, p105 and p100 in vitro (Figure 1A,B). We have also observed that NF-κB promoter activity decreases by 20–70% beginning at a concentration of 100 mM DDX in ER+ and ER− breast cancer cells and in their palbociclib-resistant counterparts (Figure 4B). This supports the role of DDX not only as an inhibitor of ribonucleotide reductase but also as an inhibitor of NF-κB activation perhaps at higher concentrations.

Cyclin D1 plays a crucial role in regulating the progress of cell cycle progression during the transition from the G1 to the S phase. The cyclin D1 gene (CCND1) is amplified in approximately 20% of breast carcinomas [35]. Intensive studies have been conducted to establish cyclin D1 as a prognostic biomarker in breast cancer [36]. The dysregulation of cyclin D1 function or gene expression causes a loss of normal cell cycle control during cancer development [35,36,37,38]. Specifically, overexpression of cyclin D1 has been proposed as a mechanism of resistance to CDK4/6 inhibitors [36]. This is the first study to examine the potential of an RR inhibitor DDX in combination with CDK4/6 inhibitor palbociclib for the treatment of ER+ and ER− breast cancers with or without palbociclib resistance. We observed that DDX inhibited cyclin D1 expression alone or in combination with palbociclib in parental and palbociclib-resistant ER+ and ER− breast cancer cells (Figure 1A,B and Figure 2A,B).

Cyclin D1 functions to form a complex with CDK4/6 and phosphorylate Rb, allowing for the progression of the cell cycle to the next DNA synthesis phase. Downregulation of pRb is the mechanism through which PLB functions [39]. It was previously reported that PLB treatment decreases the palbociclib effectors, pRb and total Rb levels, resulting in G1 arrest in breast cancer [40,41]. We have also observed that DDX treatment has the same inhibitory function on pRb and likely total Rb, as does PLB treatment (Figure 1A). Interestingly, our study confirms that upon the development of PLB resistance, PLB treatment loses its effect of reducing the expression of pRb (S807) protein levels, as evident in PLB-resistant ER+ breast cancer MCF7 cells (Figure 1A).

In ER− parental and palbociclib-resistant MDA-MB-468 (Rb-deficient) breast cancer cells, DDX also downregulated cyclin E2 and cyclin A2 and unexpectedly restored the presence of the cell cycle repressor Rb, which is an integral part of the cell cycle checkpoint inhibiting the progression past G1 (Figure 1B and Figure 2B). The ability to restore Rb to Rb-deficient breast cancers may improve the efficacy of PLB treatment when given in combination with ribonucleotide reductase inhibitors such as DDX.

Here, we demonstrate a comprehensive analysis of the impact of RR inhibition by DDX in combination with the CDK4/6 inhibitor palbociclib on the cell cycle in parental and palbociclib-resistant ER+ and ER− breast cancer cells. These data reveal that DDX reduces the protein levels of RRM2, NF-κB and cyclin D1, and Rb, resulting in a significant accumulation of cells at the G1 phase of the cell cycle arrest in the cell cycle (Figure 3A–D). We also demonstrate that palbociclib treatment alone fails to cause cell cycle arrest at the G1 phase in both ER+ and ER− palbociclib-resistant breast cancer cells (Figure 3C,D). These data are consistent with the inhibitory effects of DDX on the cell cycle. The unique mechanism of action of DDX with palbociclib to block the progression of the cell cycle supports a strategy for combination therapies for patients with ER+ and ER− parental and palbociclib-resistant breast cancer. In addition, we observed a significant increase in pH2AX levels in DDX-treated ER+ and ER− breast cancers, which confirms the DNA damaging effect of the drug (Figure 1A,B). Yet, DDX has exhibited acceptable toxicity in various animal models and in early clinical trials [42,43,44,45,46,47].

Our previous study showed downregulation of mutant p53 with DDX treatment without any negative impact on wild-type p53 in triple-negative breast cancers [14]. In this study, we show that DDX has a similar inhibitory effect on mutant p53 in parental ER− MDA-MB-468 breast cancer cells and in their palbociclib-resistant counterpart cells in a time-dependent manner (Figure 1B). This inhibitory effect was nonexistent in ER+ breast cancer with wild-type p53 and its palbociclib-resistant counterpart (Figure 1A).

## 5. Conclusions

In summary, our findings strongly support the hypothesis that DDX in combination with palbociclib can potentially treat ER+ and ER− breast cancers and perhaps prevent palbociclib resistance by targeting RRM2, NF-κB, cyclin D1 and pRb. Also, DDX successfully targets mutant p53 in ER− breast cancer with no inhibition of wild-type p53. We also found evidence that RRM2 and cyclin D1 levels are upregulated in ER− palbociclib-resistant breast cancer, giving rise to a difficult-to-treat breast cancer population. Reduced expression of RRM2, cyclin D1, and NF-κB protein, and an elevated level of pH2AX result in decreased breast cancer growth and survival. We have also observed that the addition of DDX halts cell cycle progression at G1. Ultimately, our data suggest a strategy to target RRM2, NF-κB protein, and cyclin D1 to supplement traditional therapies while offering improved efficacy for breast cancer treatment.

The restoration of Rb in MDA-MB-468 (Rb-deficient) ER− breast cancer cells after DDX treatment appears to re-sensitize the cancer cells to palbociclib therapy. However, the exact mechanism of this effect is still unknown. Future studies need to be performed to provide a path for the development of new drug molecules targeting the restoration of Rb status in ER− Rb-deficient breast cancer.

## Data Availability

Data is contained within the article.

## Supplementary Materials

The following supporting information can be downloaded at: https://www.mdpi.com/article/10.3390/cancers16050975/s1.
