# Supplementary material for: Targeting the Cell Cycle, RRM2 and NF-κB for the Treatment of Breast Cancers
Source: Cancers (Basel). 2024 Feb 28;16(5):975. doi: 10.3390/cancers16050975 (PMC10930692; doi:10.3390/cancers16050975)

1A

Cyclin E2

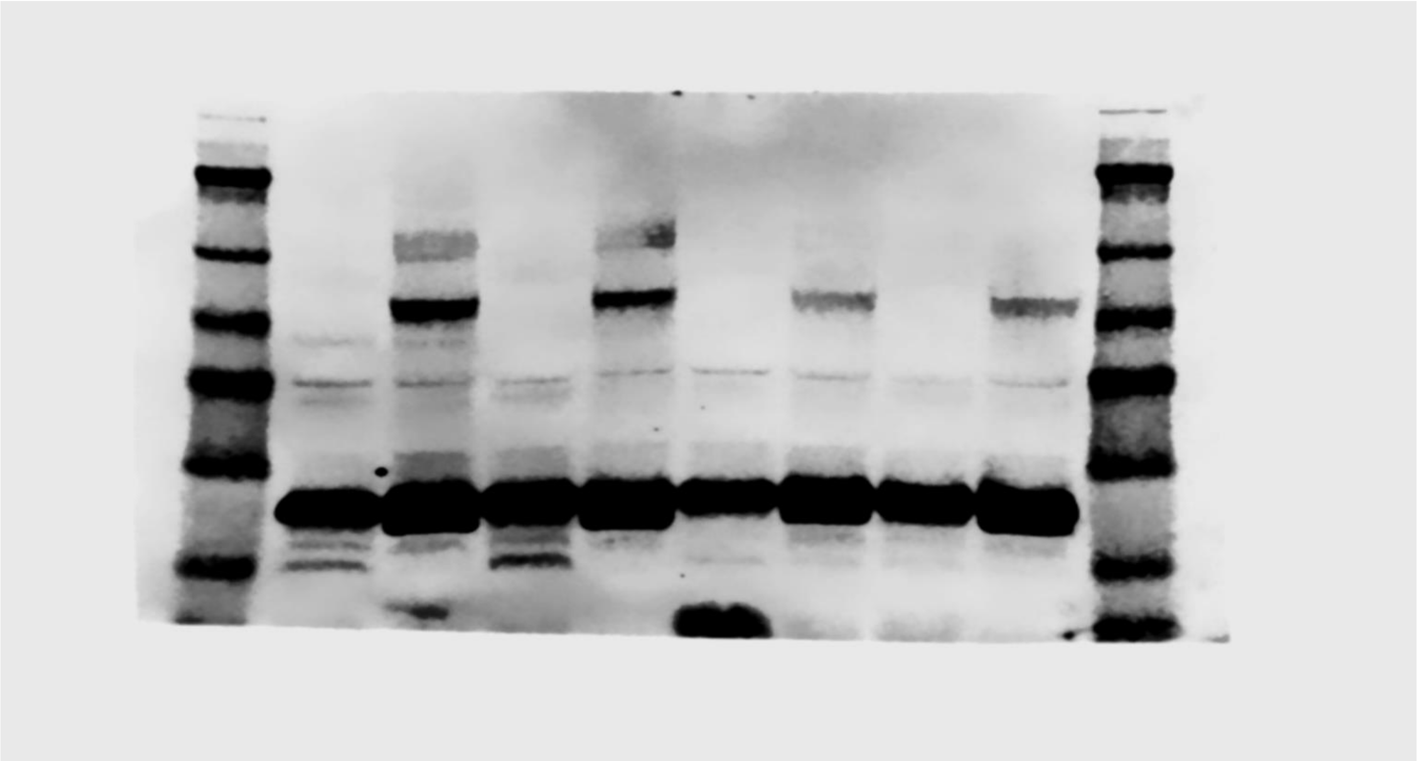

1A

Cyclin B1

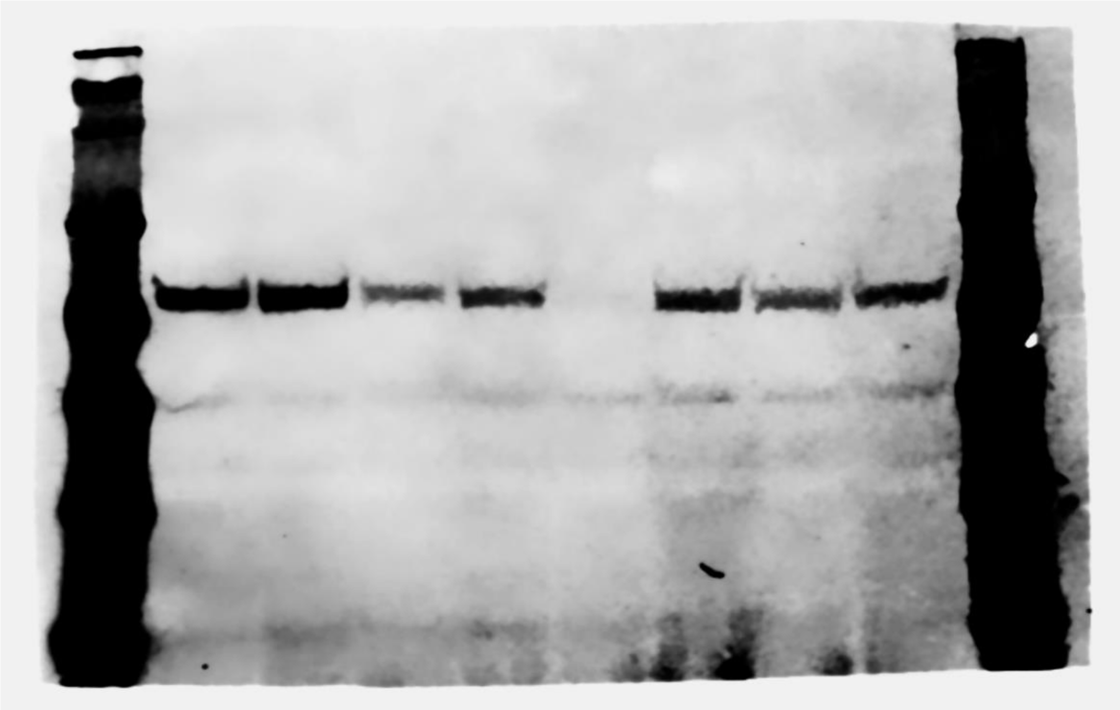

1A

Cyclin A2

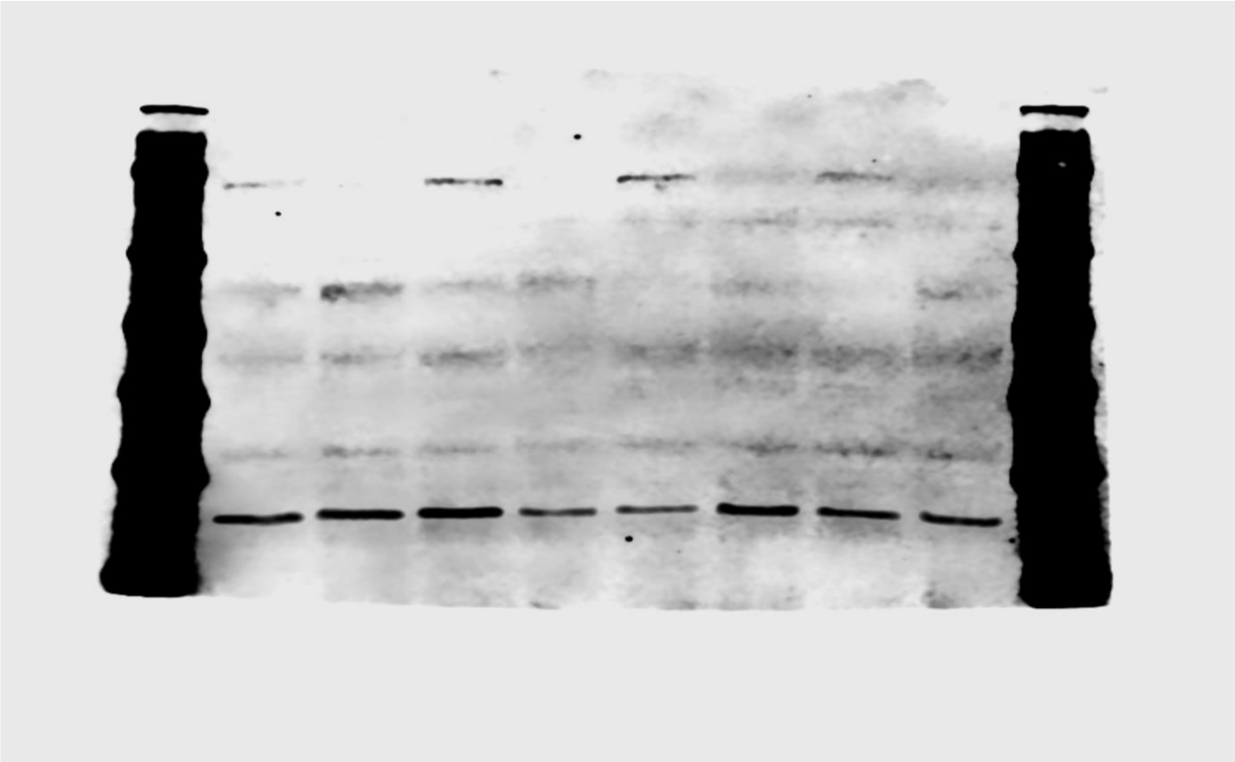

1A

Cyclin D1

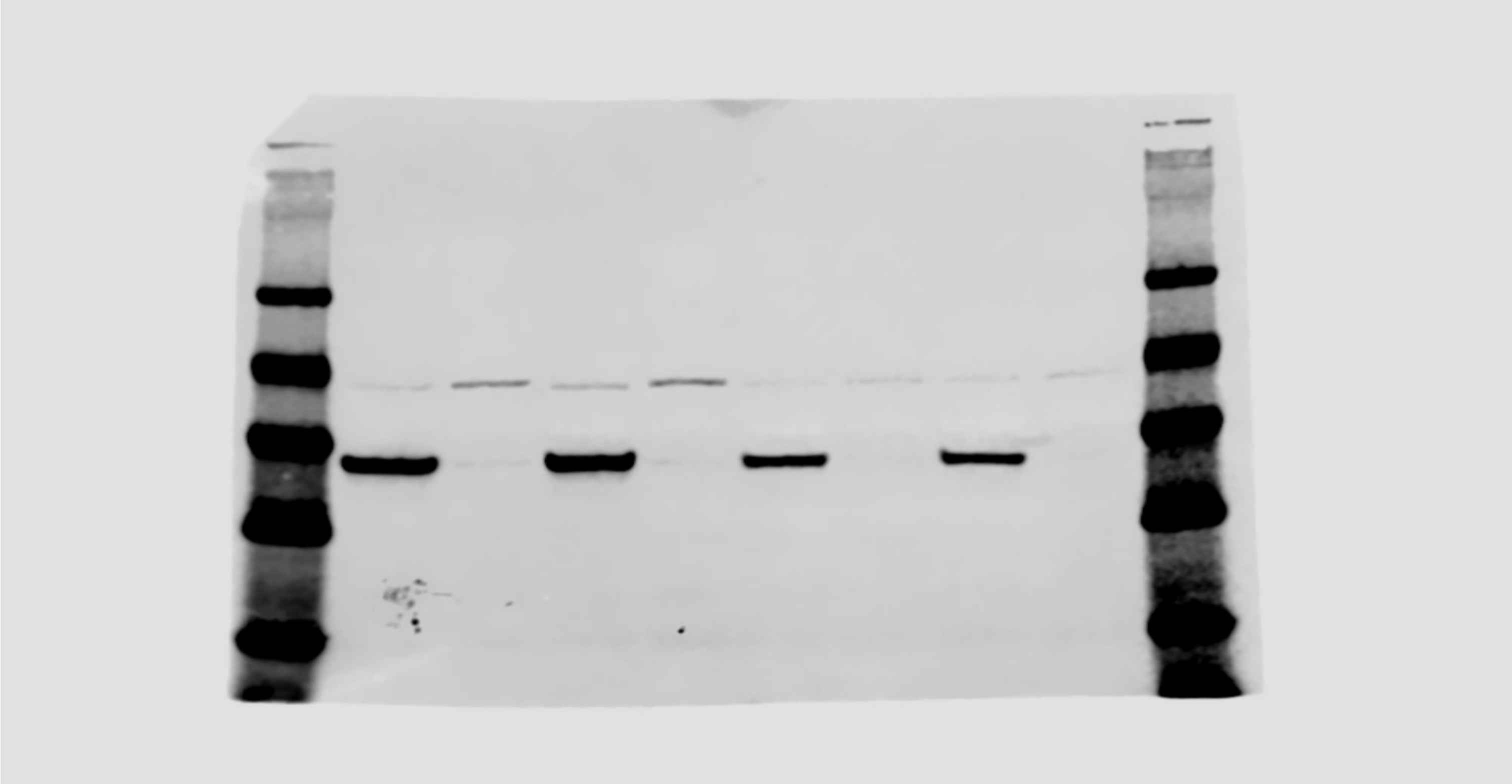

1A

pRb(S807)

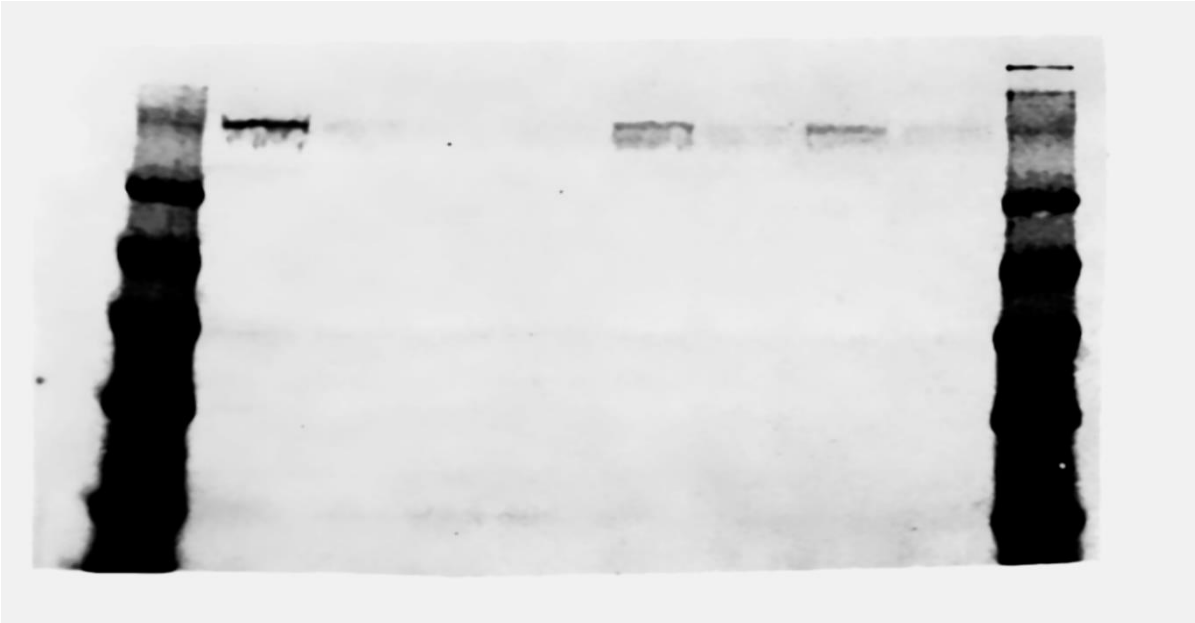

1A

Rb

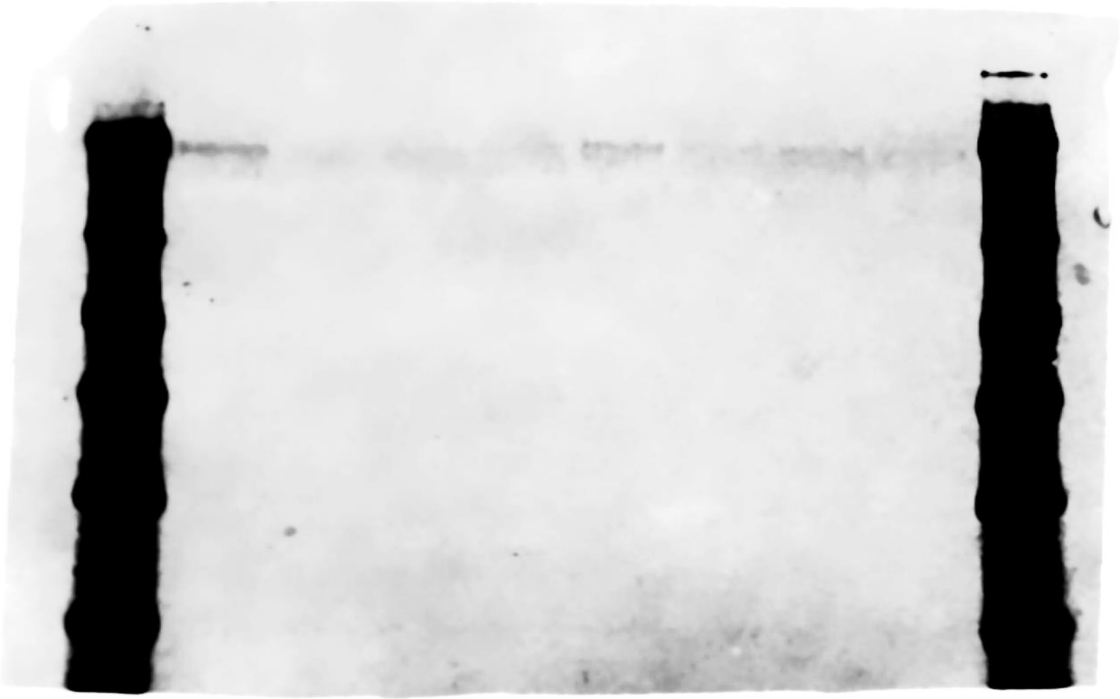

1A

p21

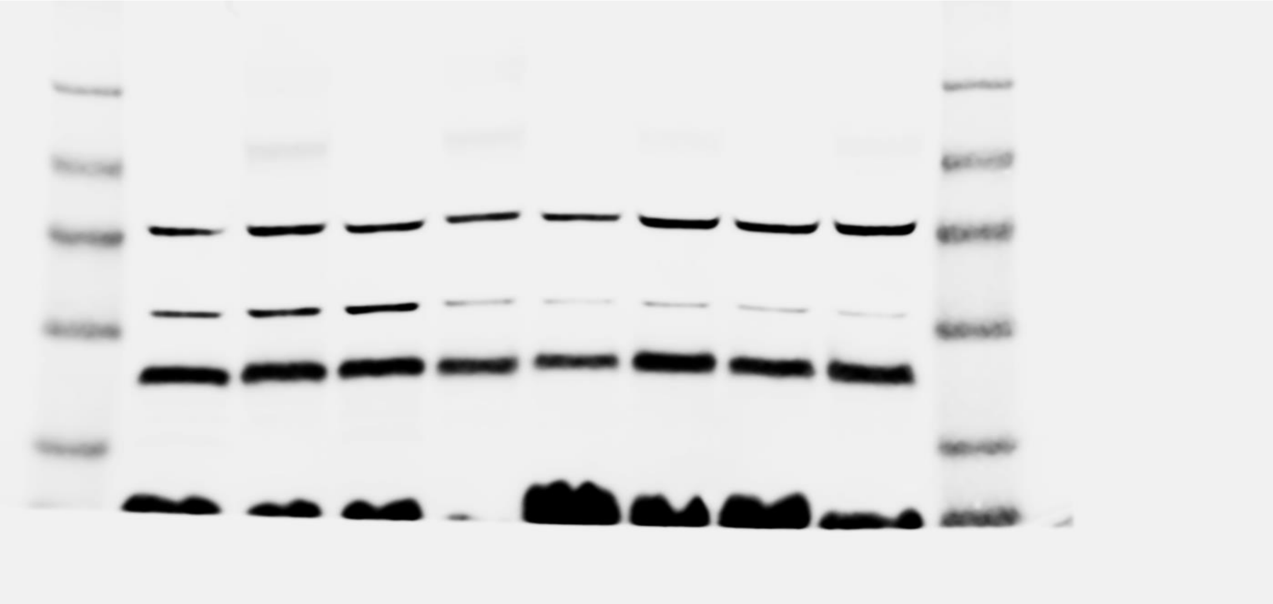

1A

pp53 (S392)

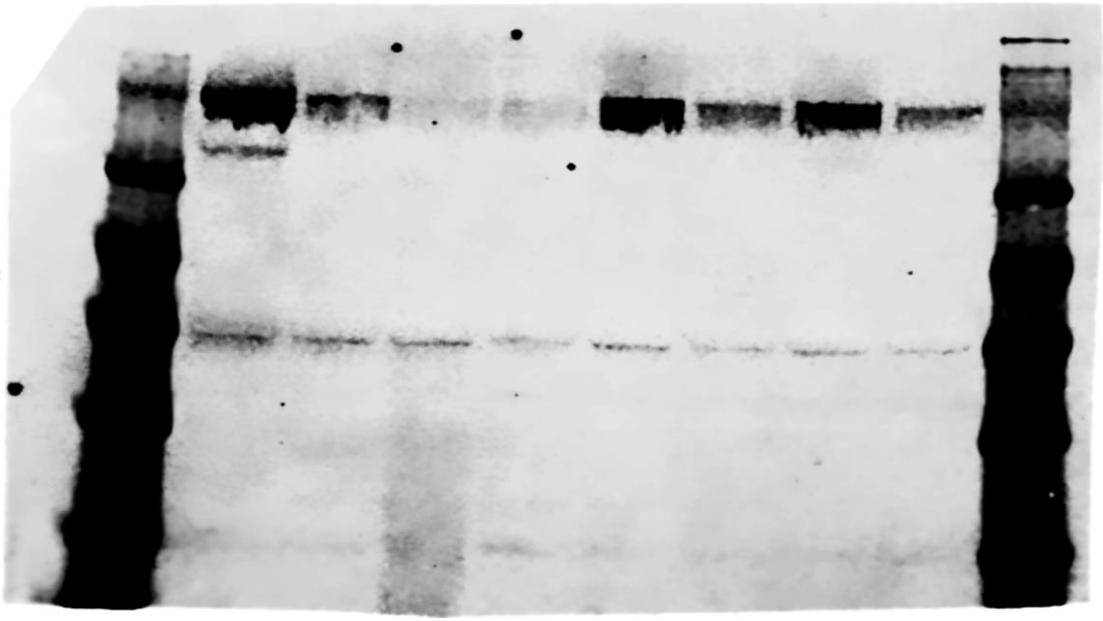

1A

p53

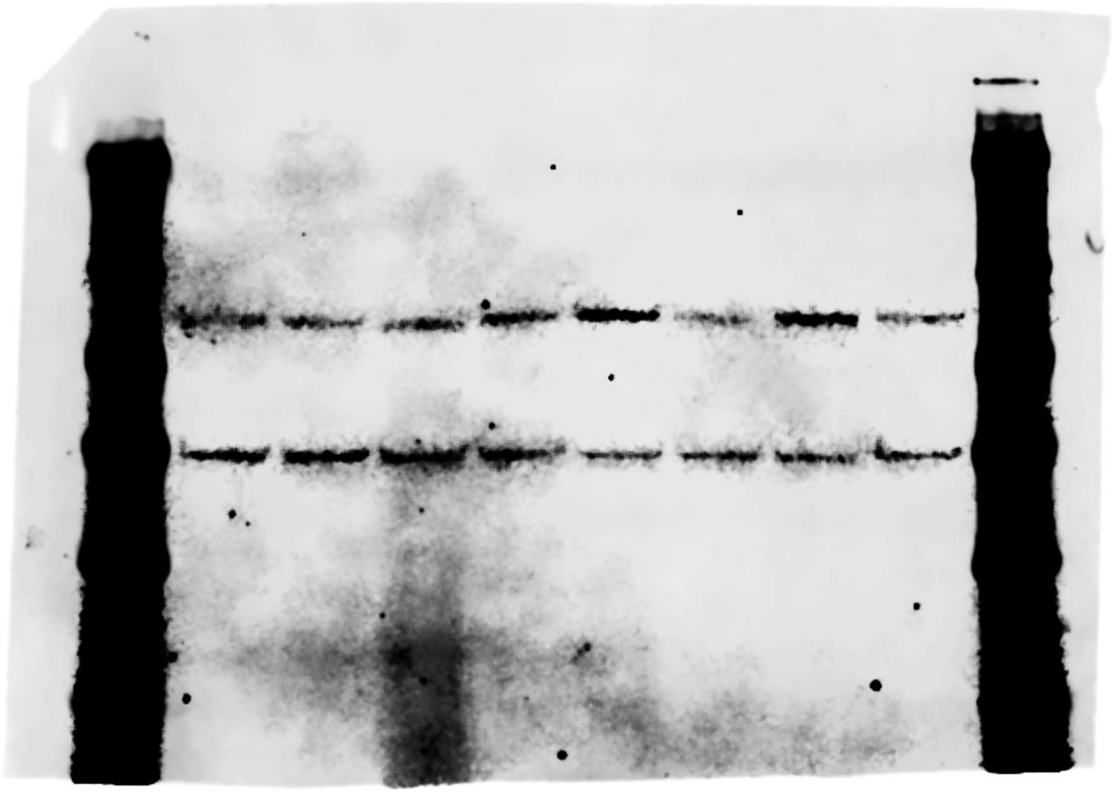

1A

Mut p53

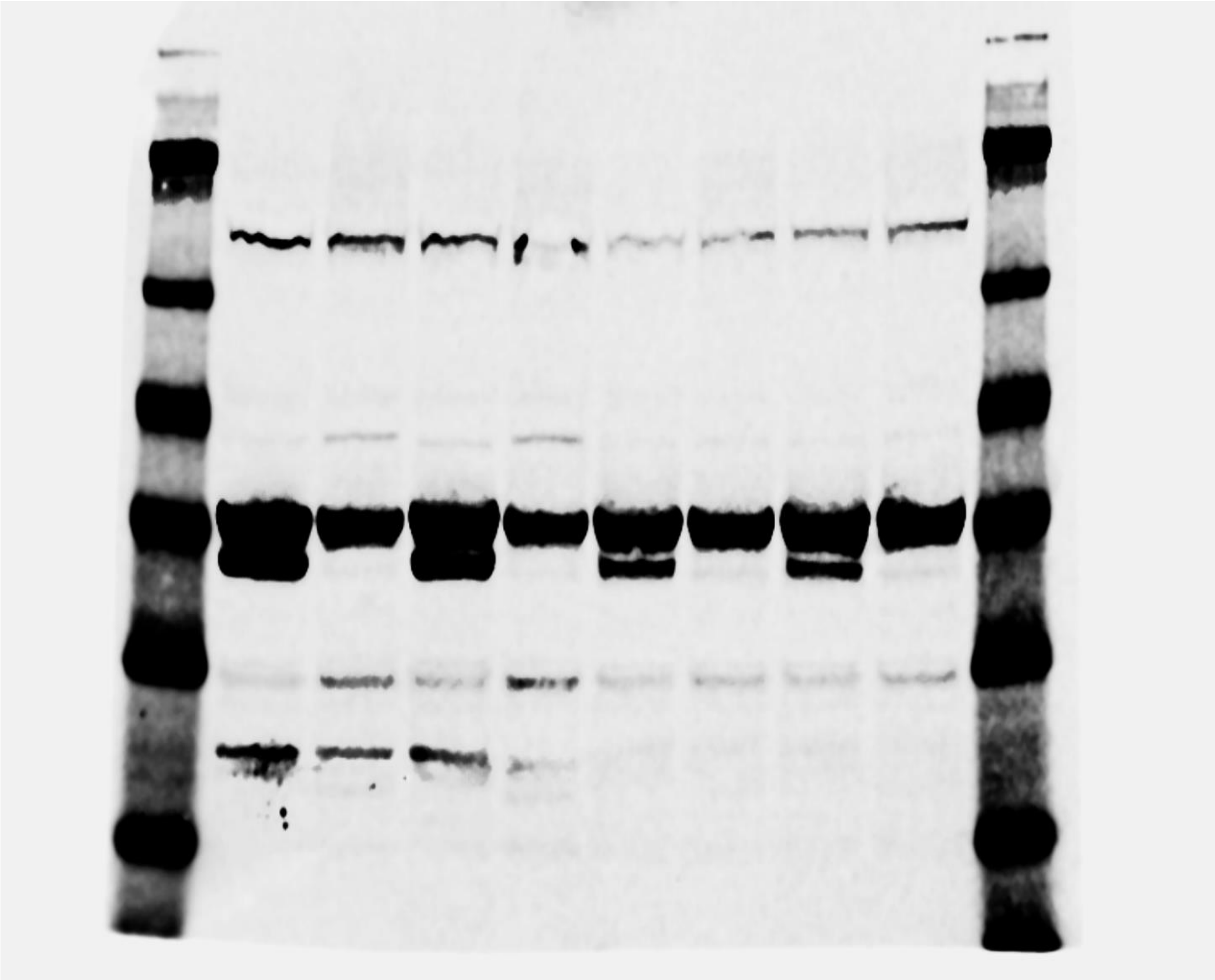

1A

pAkt (S473)

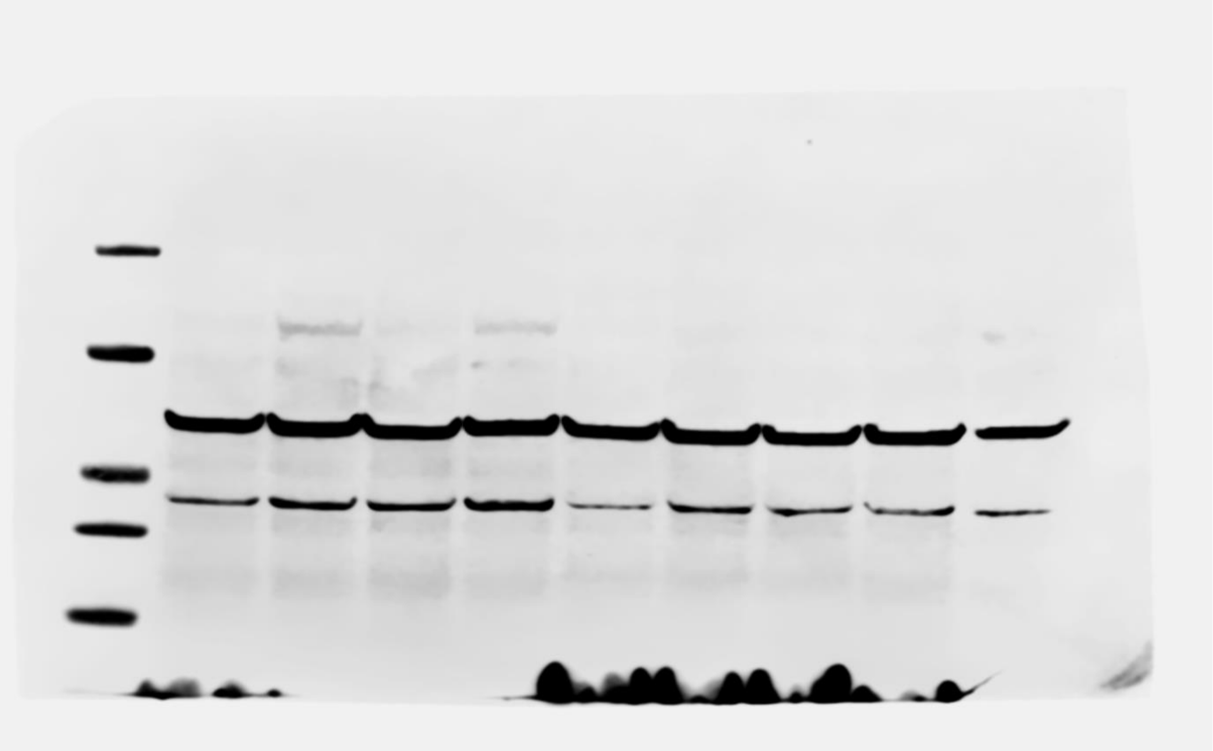

1A

pAkt (T308)

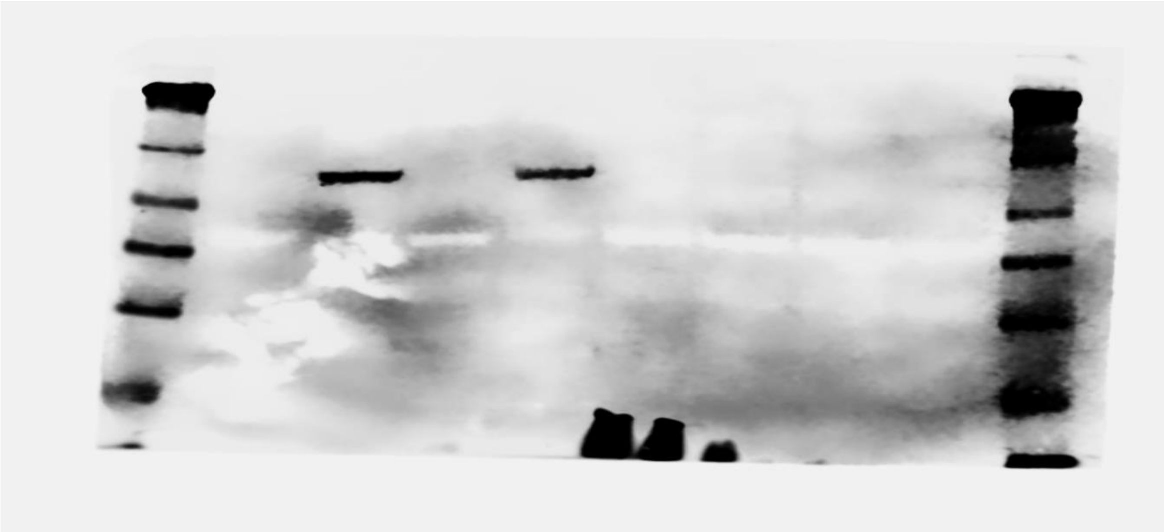

1A

Akt

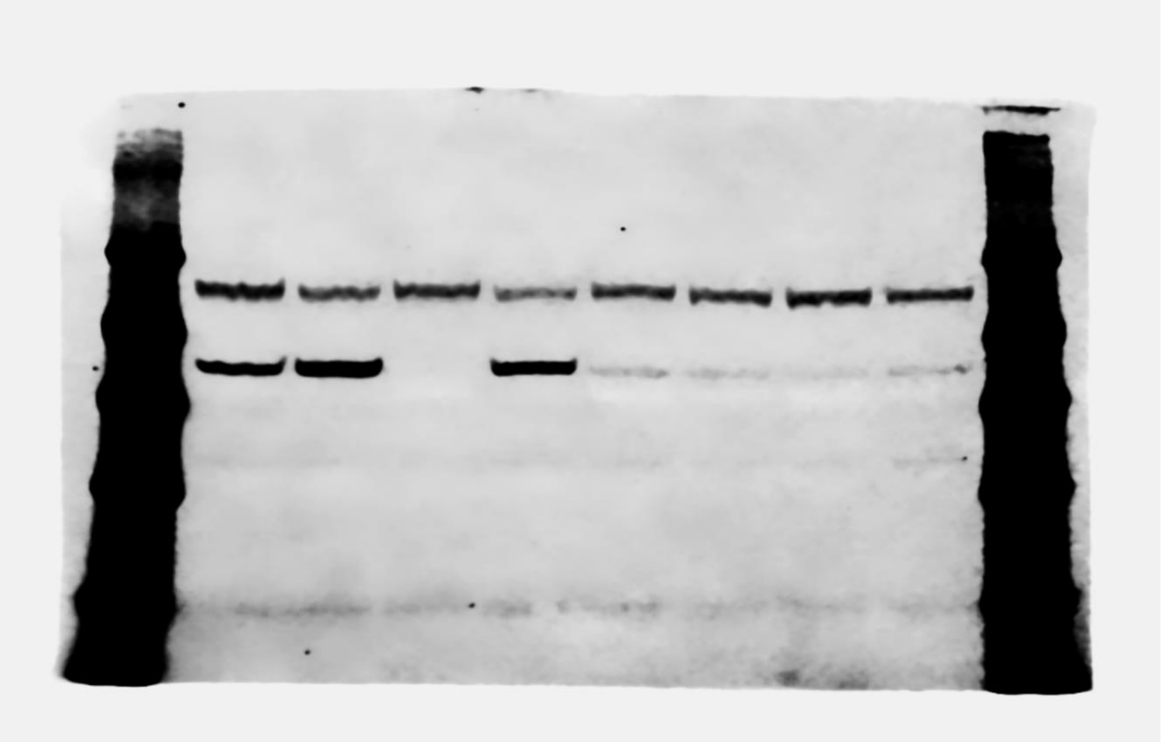

1A

p100

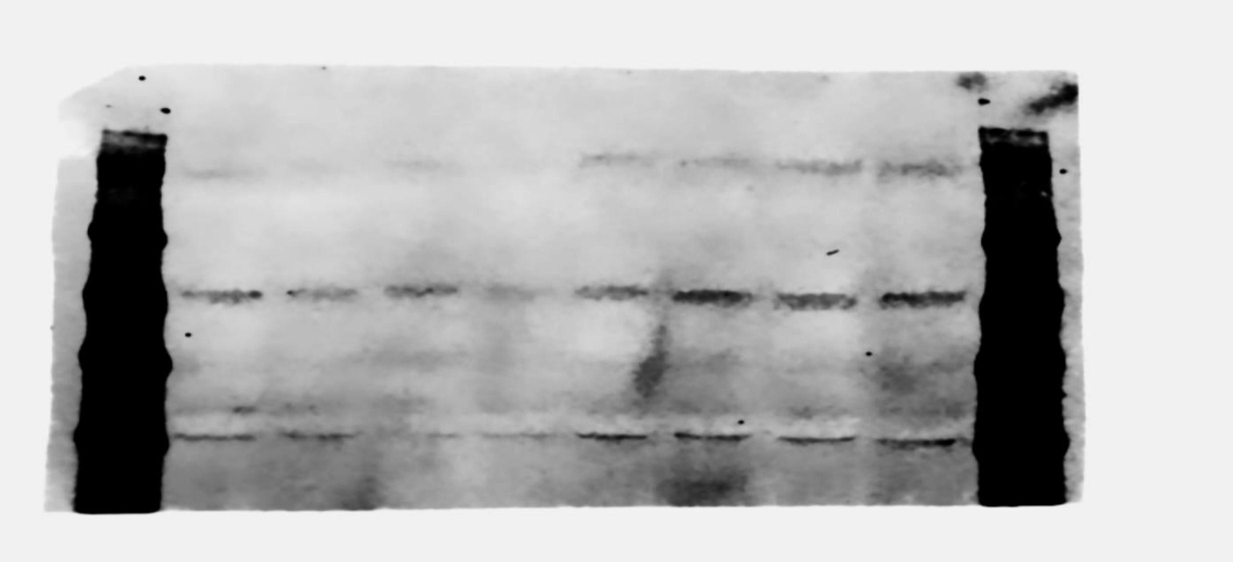

1A

p105

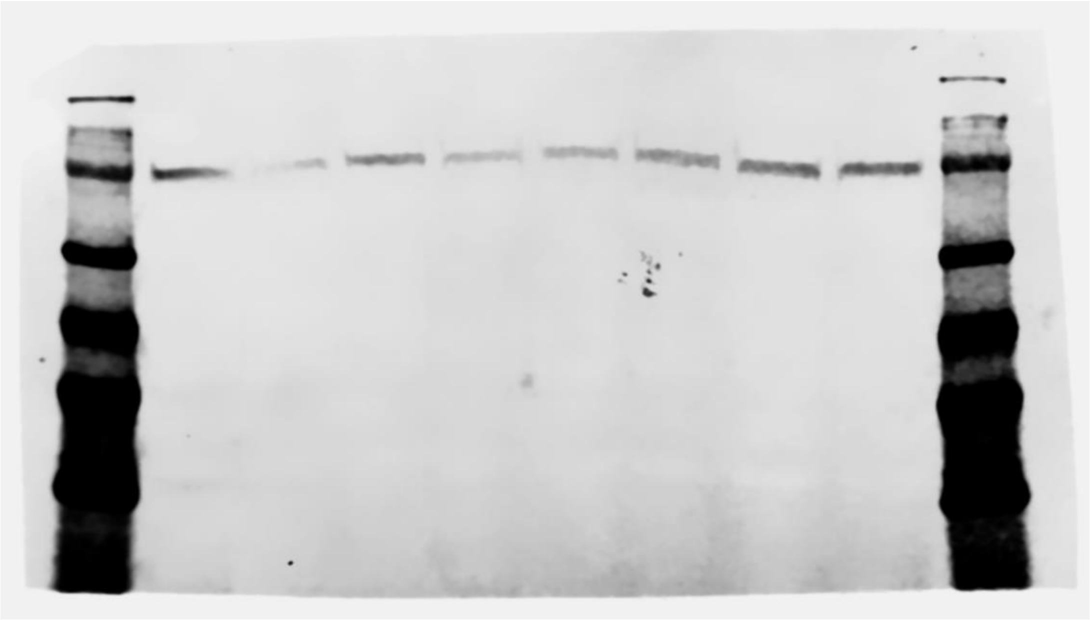

1A

RelB

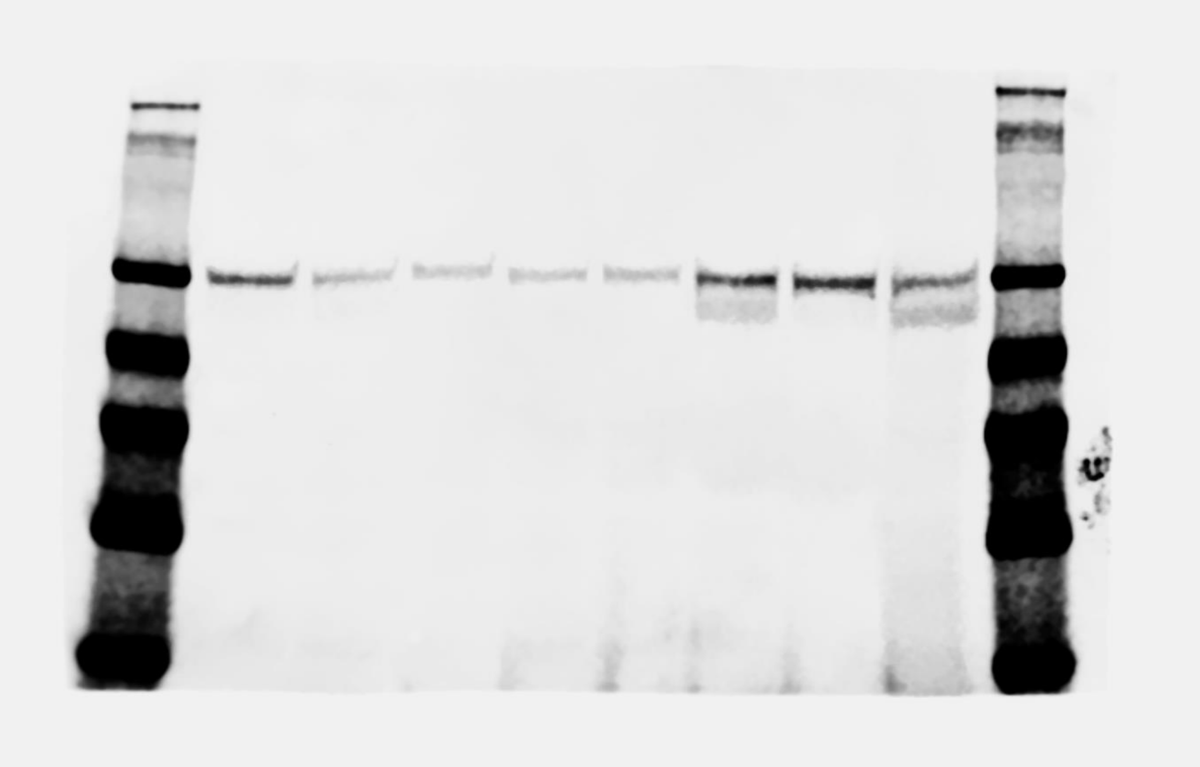

1A

cRel

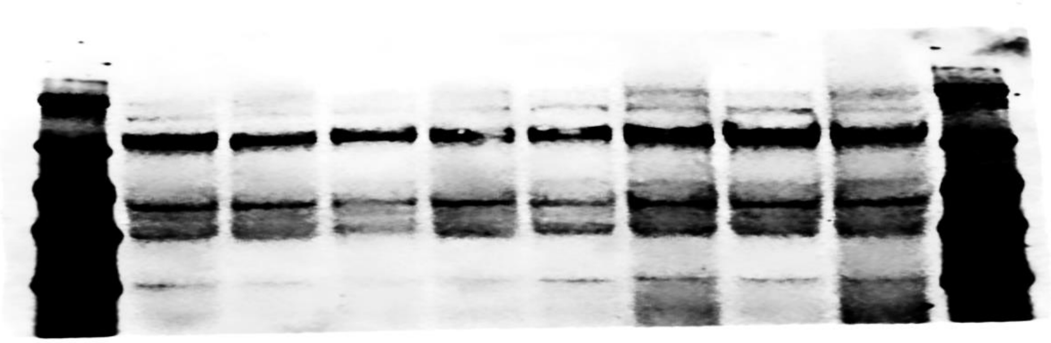

1A pIKKα/β

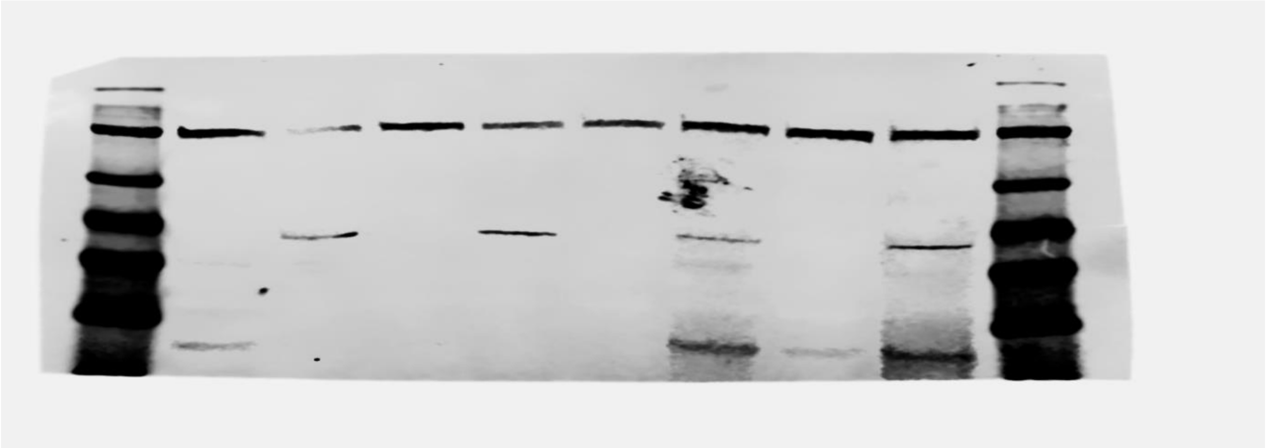

1A

IKKα

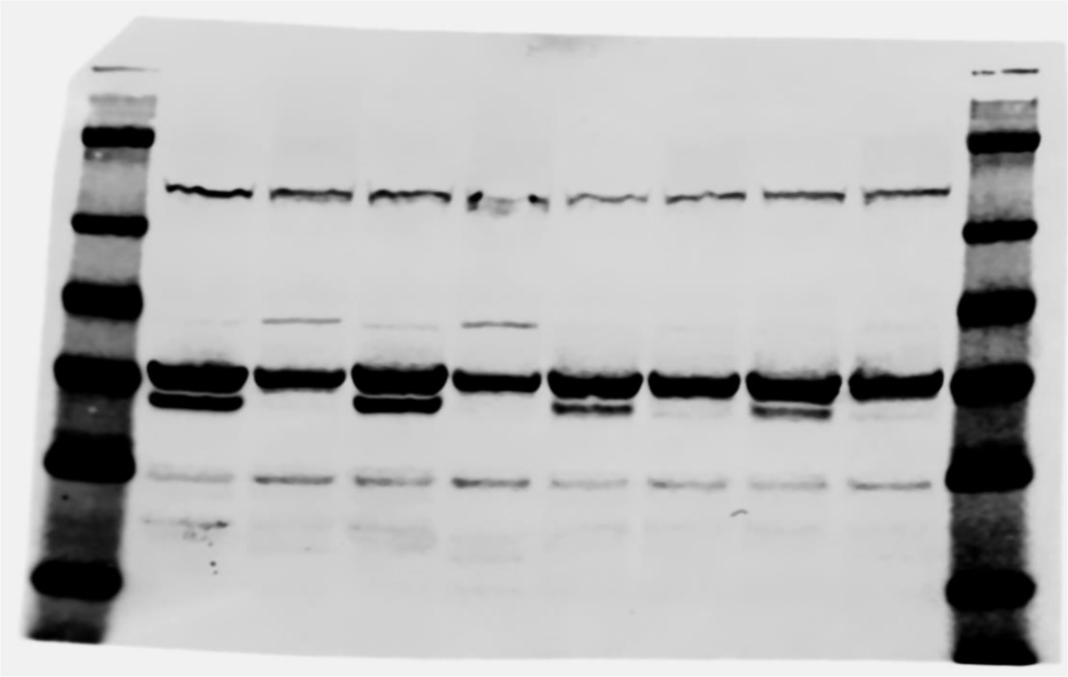

1A

IKKβ

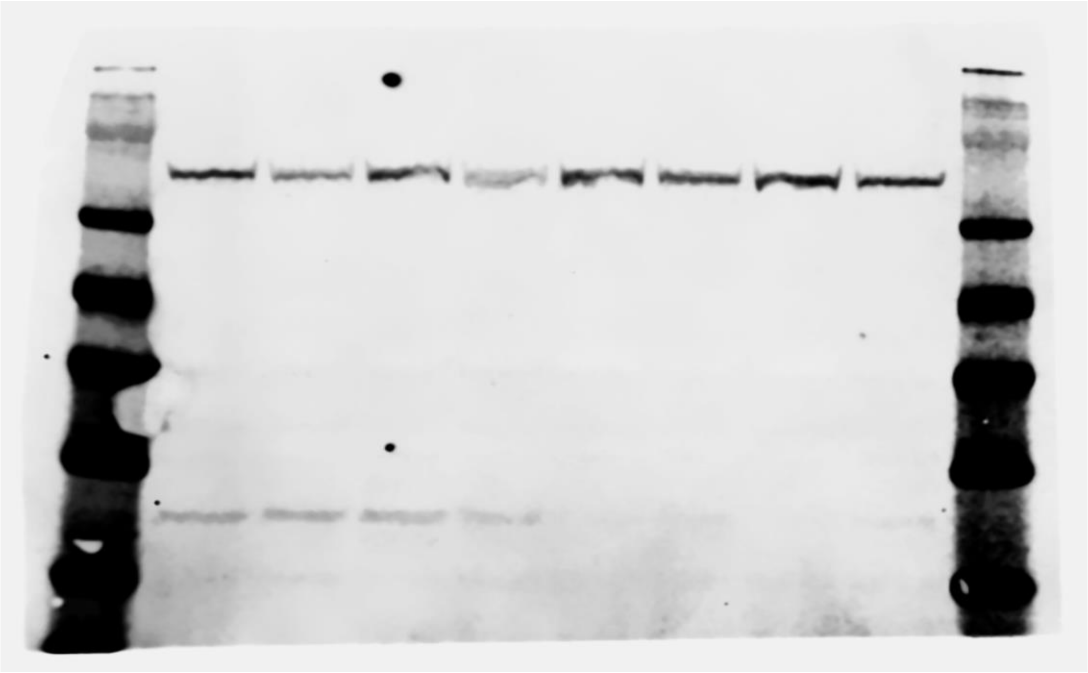

1A

pIKBα

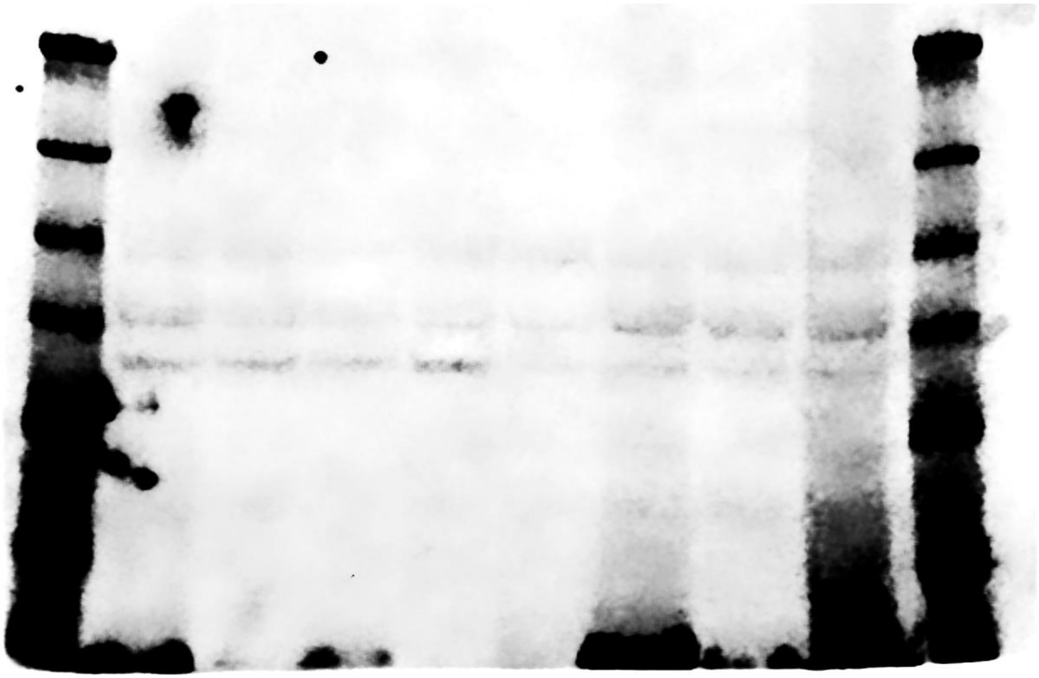

1A

IKBα

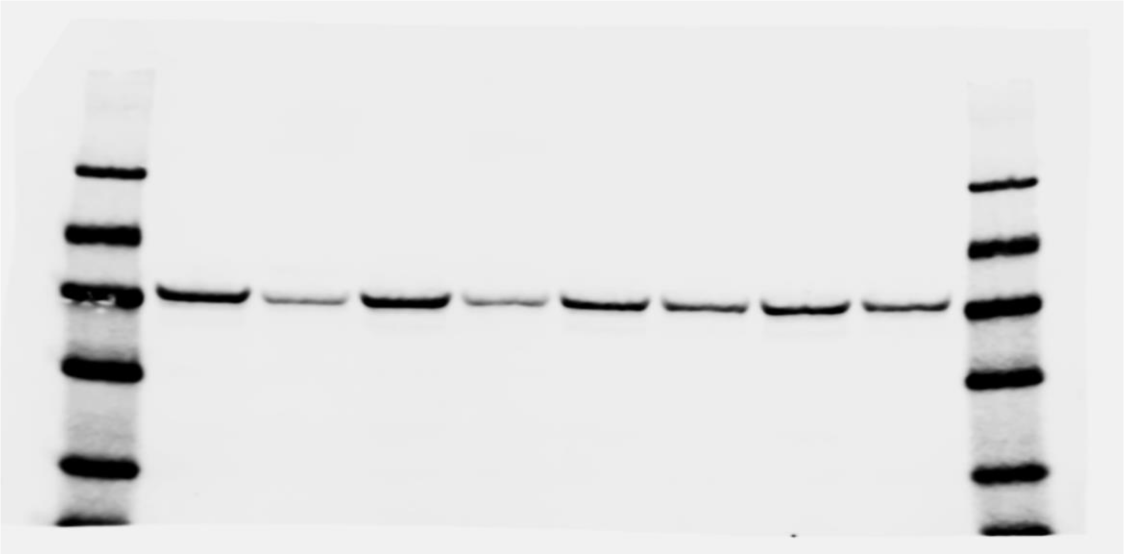

1A pH2AX

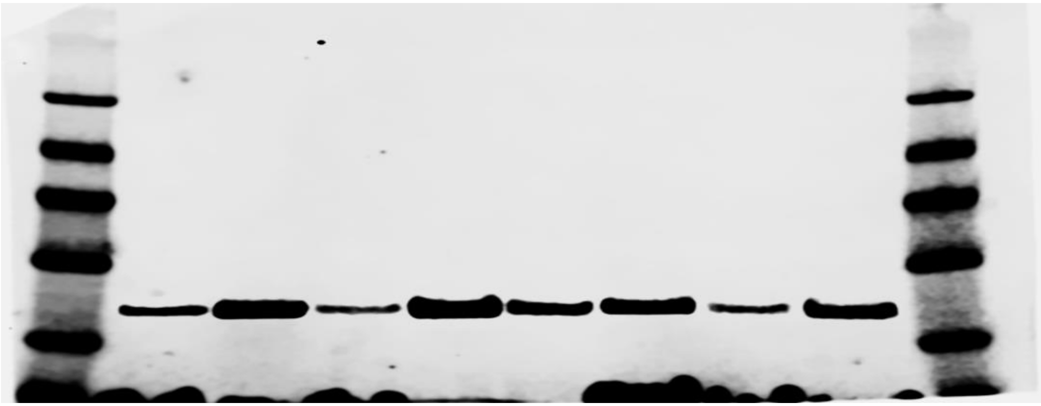

1A H2AX

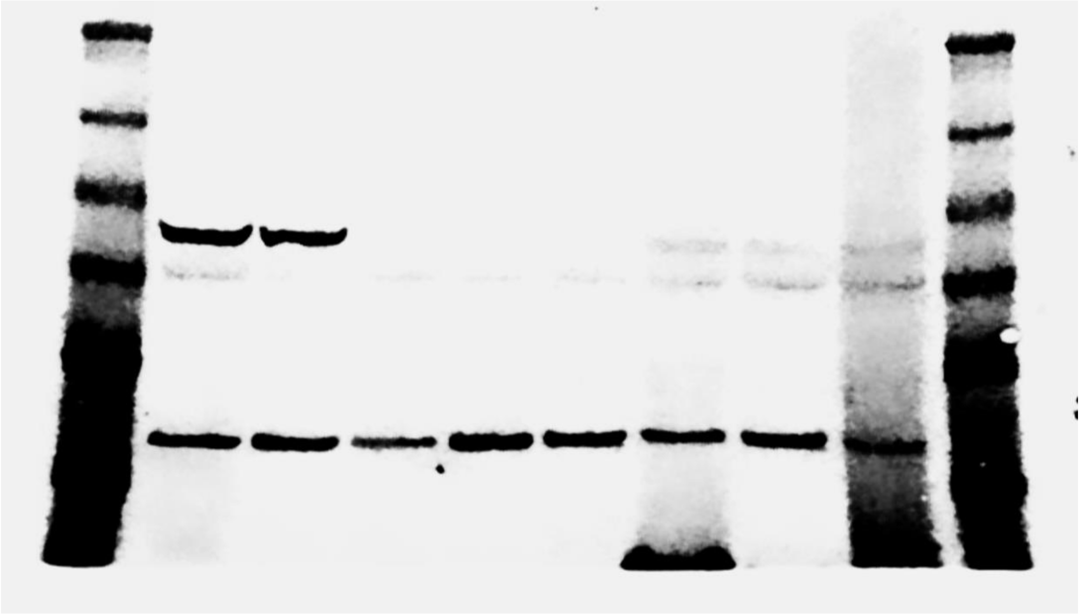

1A

ER alpha 66

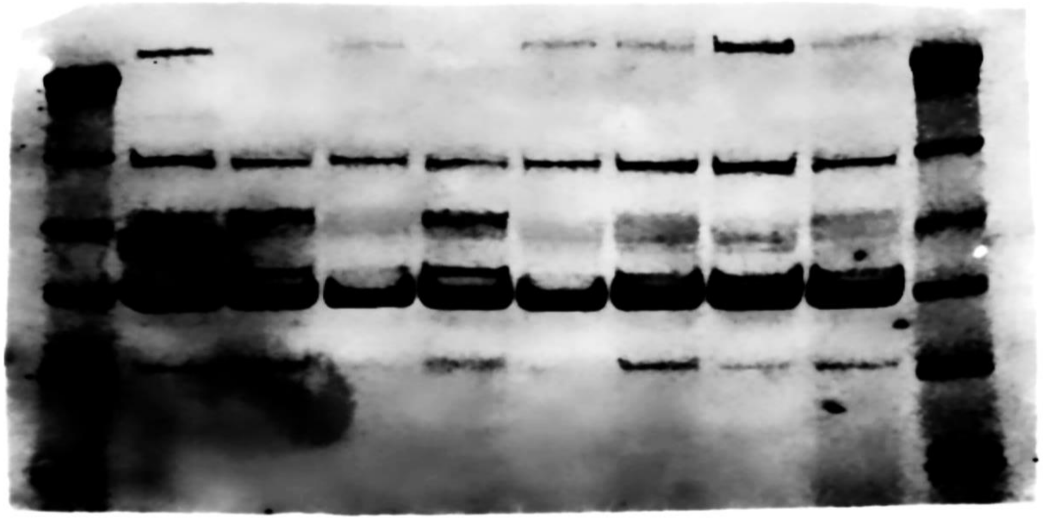

1A RRM2

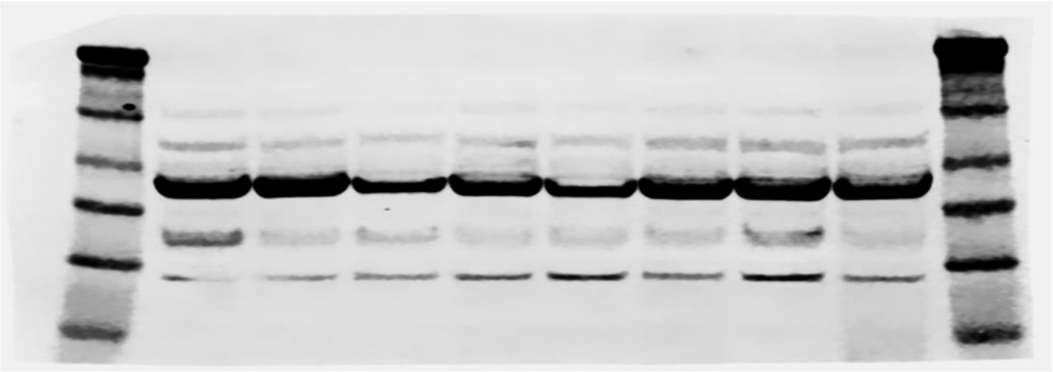

1A GAPDH

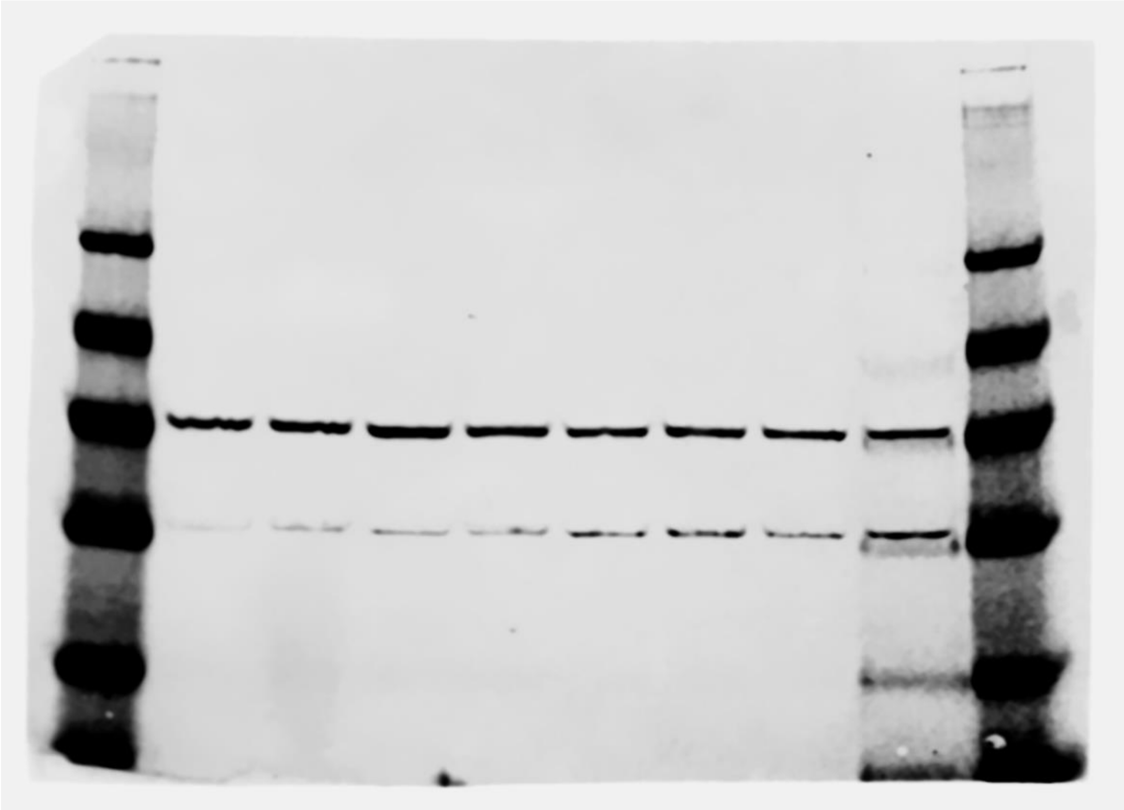

1B

Cyclin E2

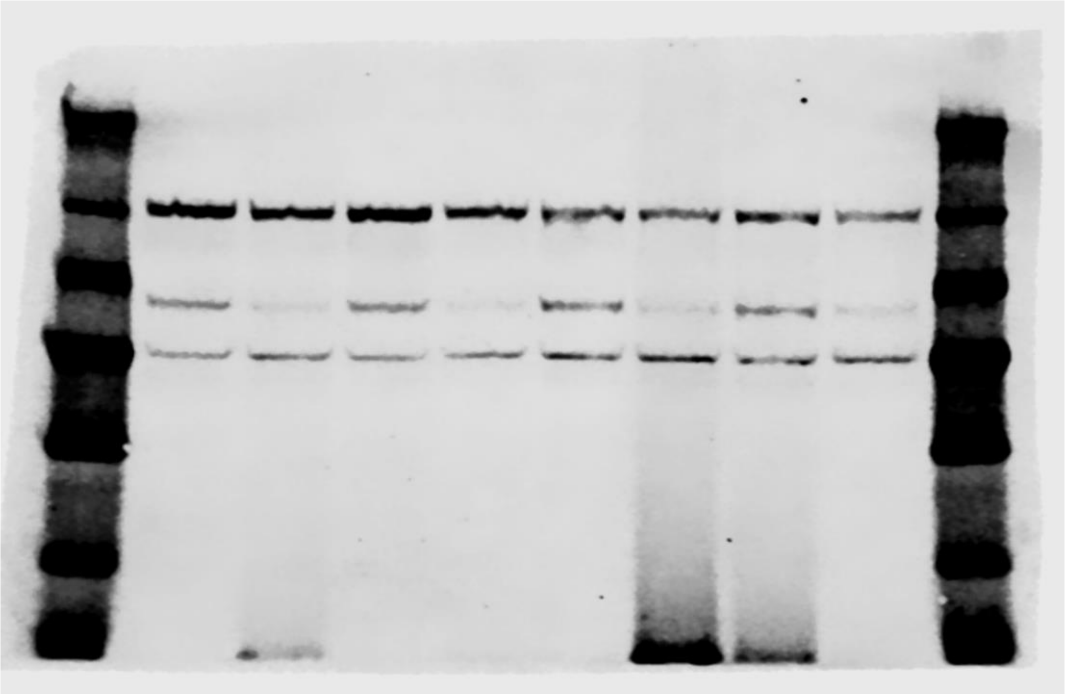

1B

Cyclin B1

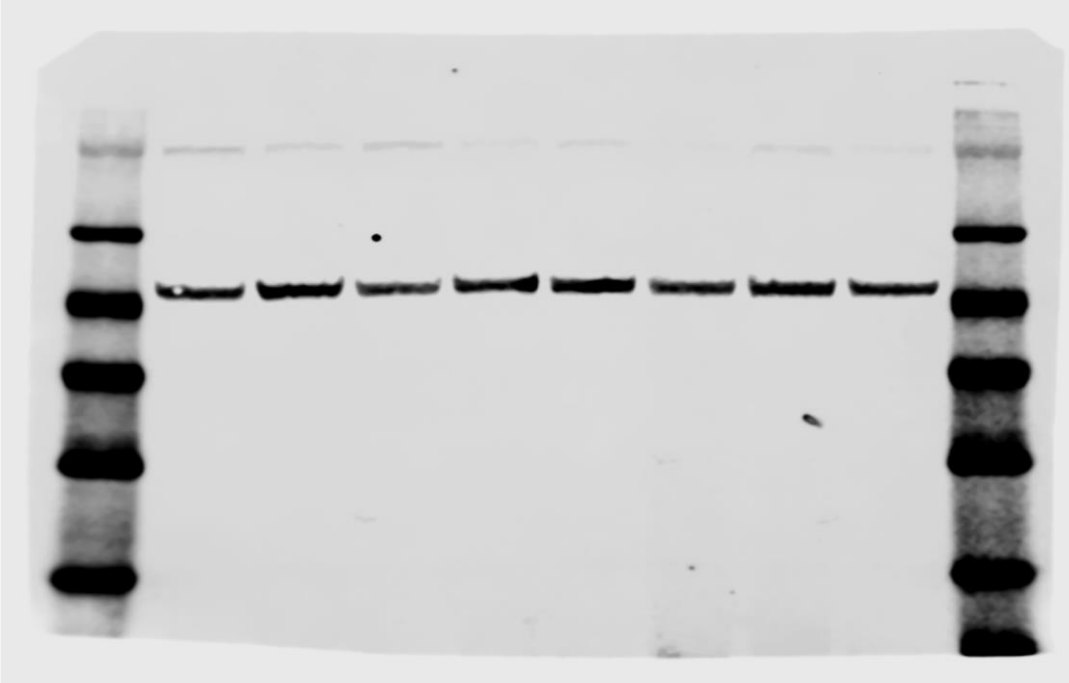

1B

Cyclin A2

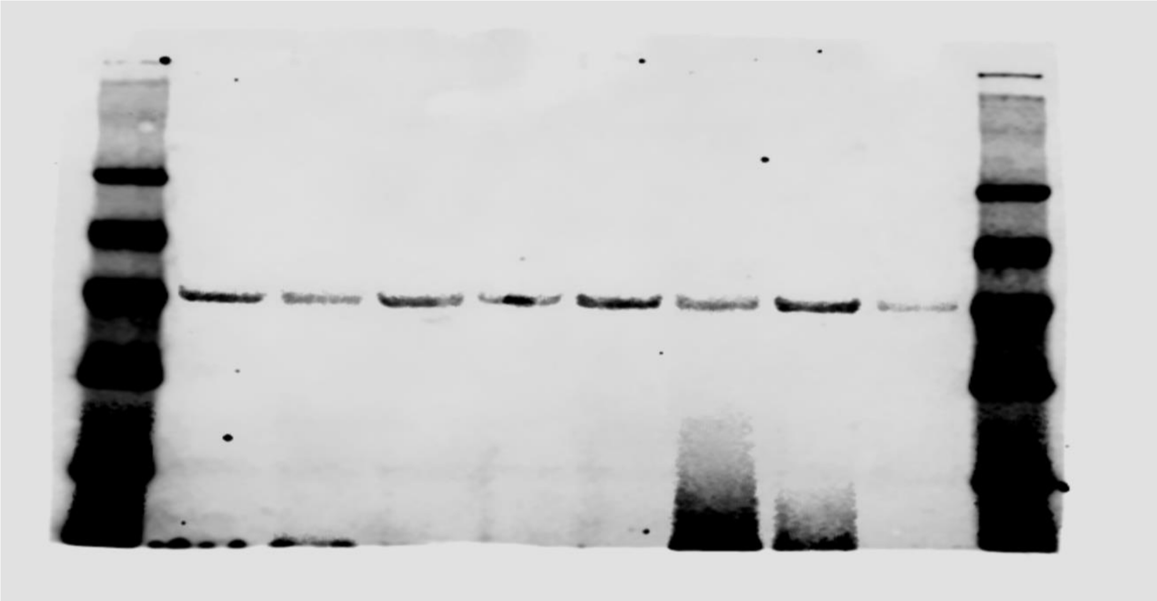

1B Cyclin D1

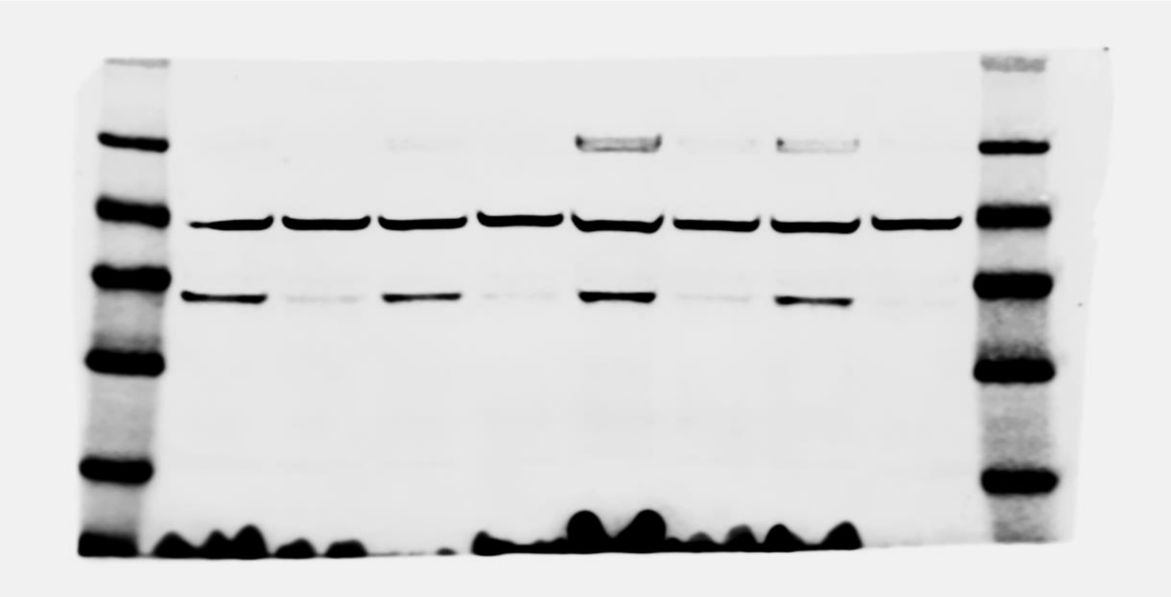

1B

pRb (S807)

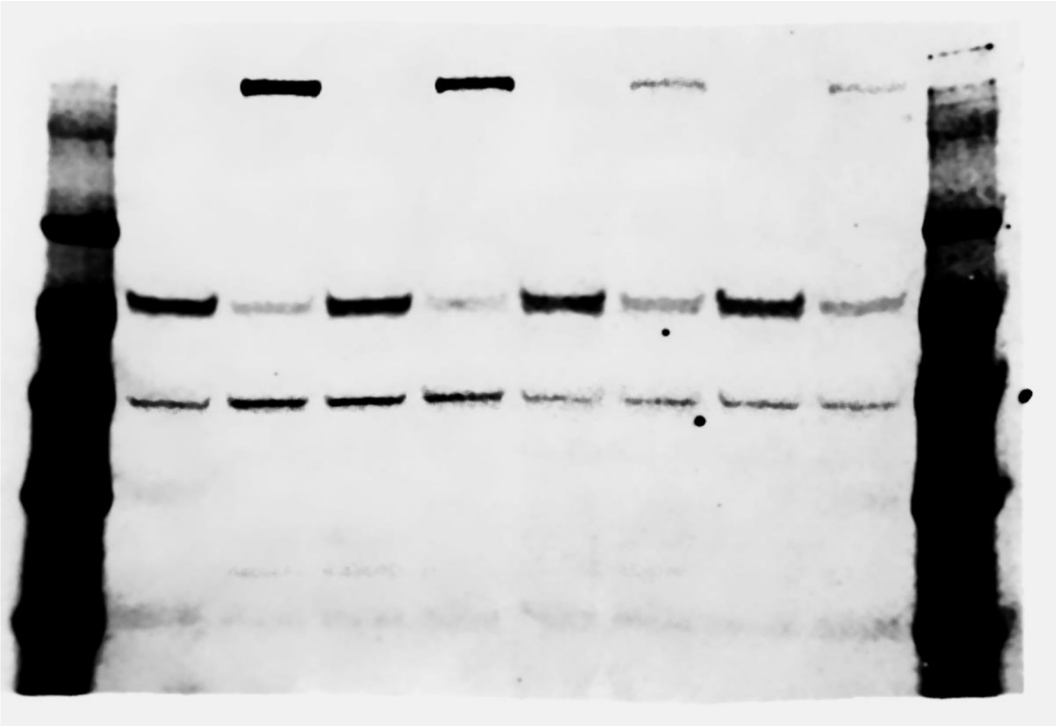

1B

Rb

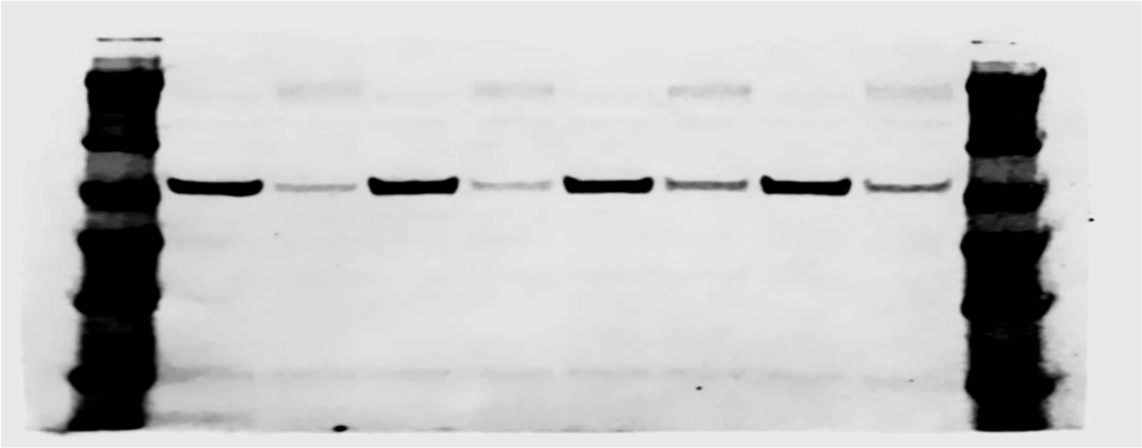

1B

p21

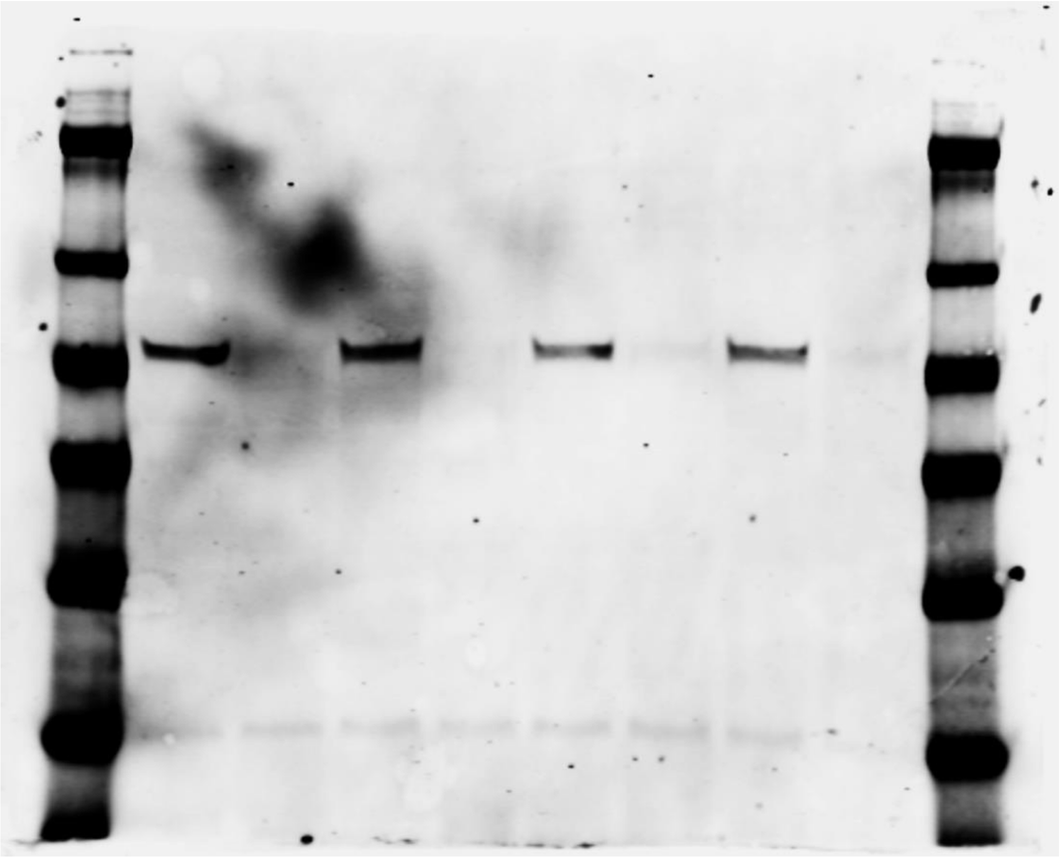

1B

pp53 (S392)

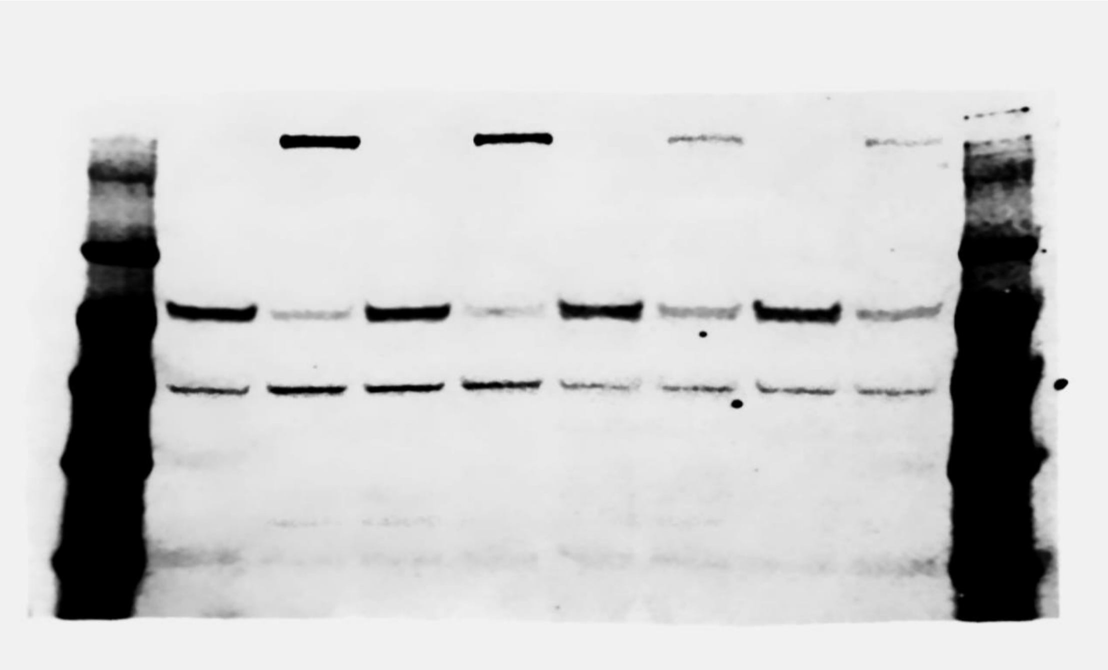

1B

p53

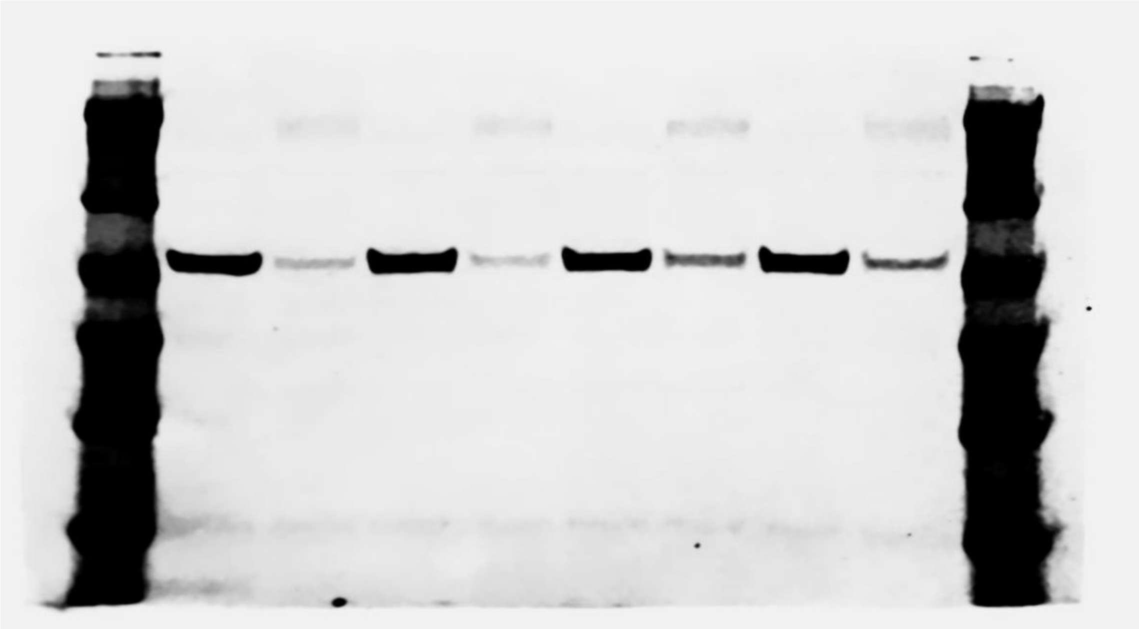

1B

Mut p53

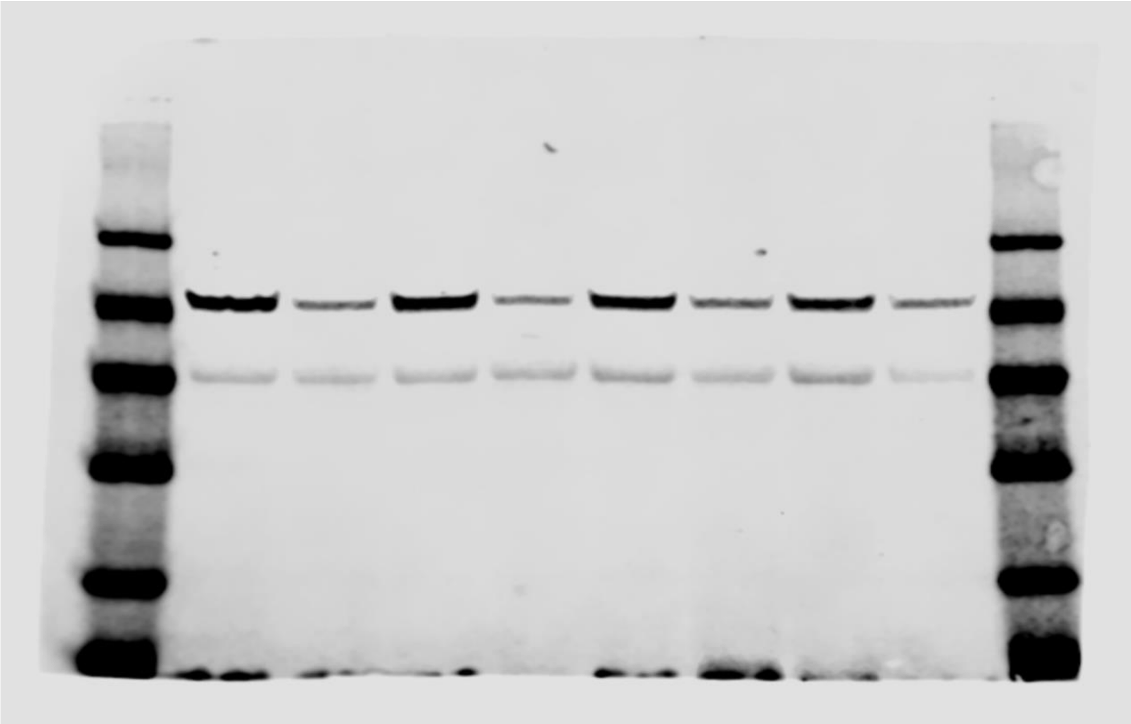

1B

pAkt (S473)

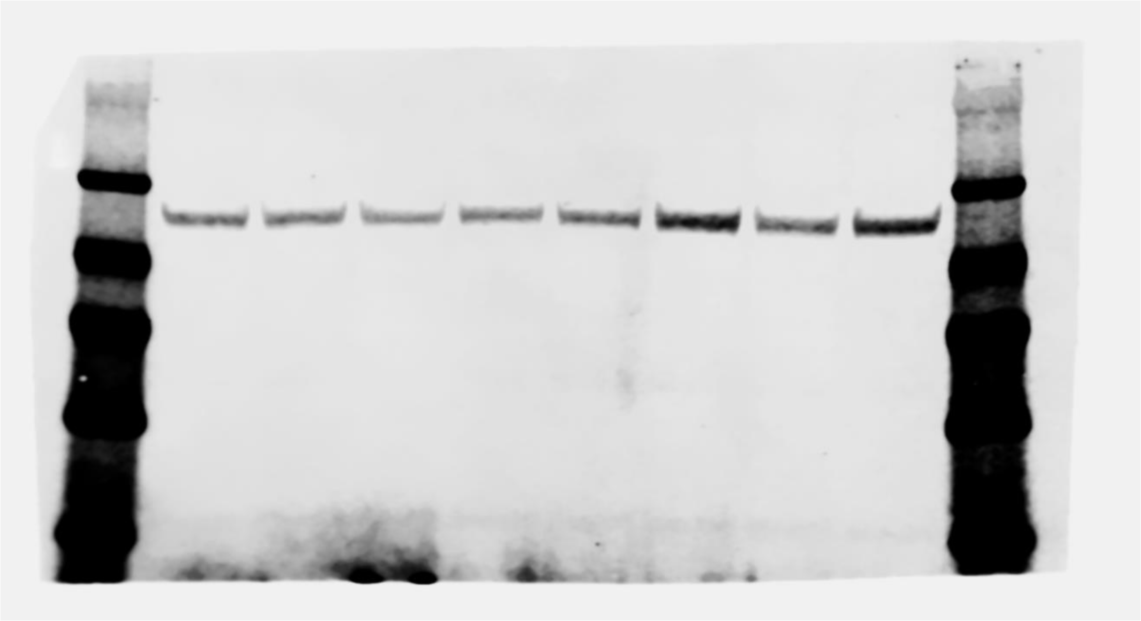

1B

pAkt (T308)

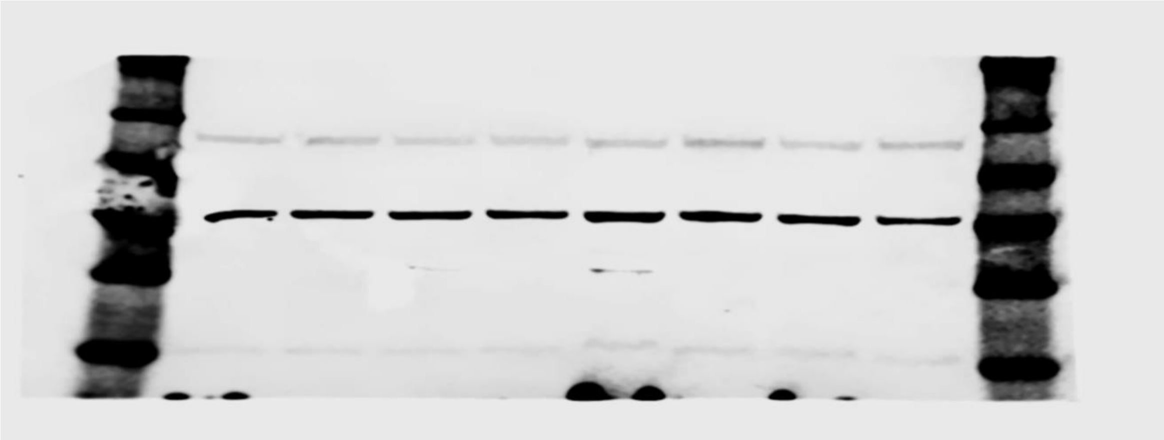

1B

Akt

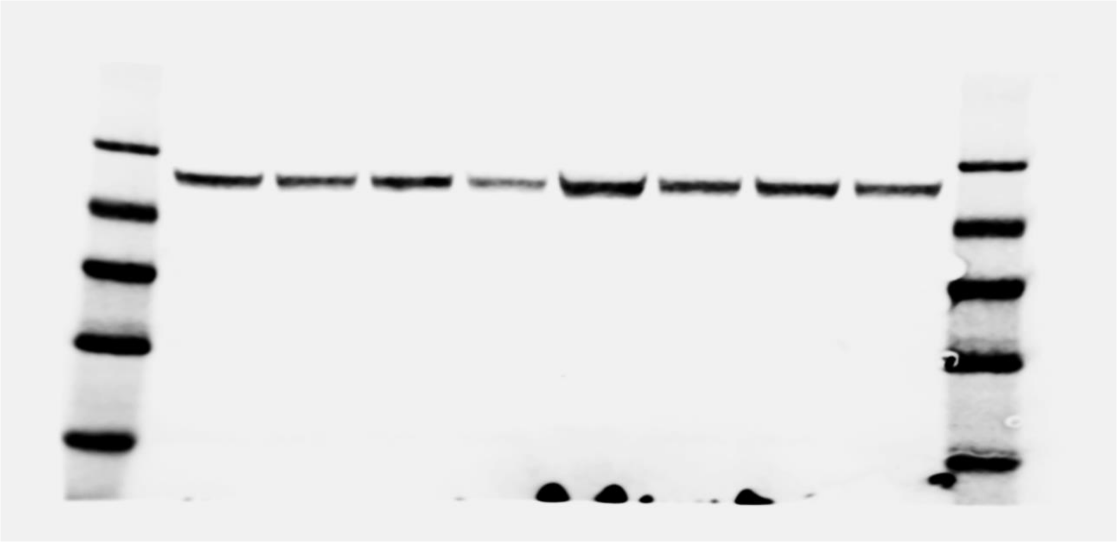

1B

p100

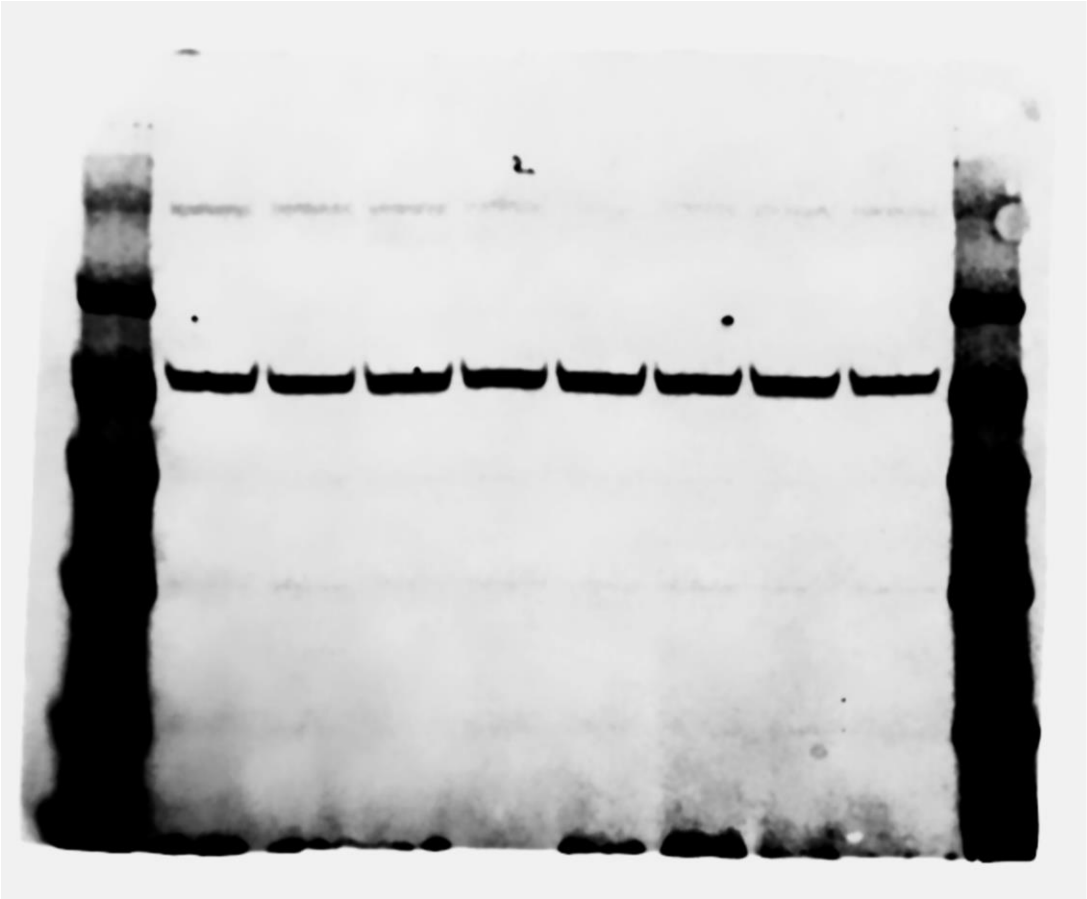

1B

p105

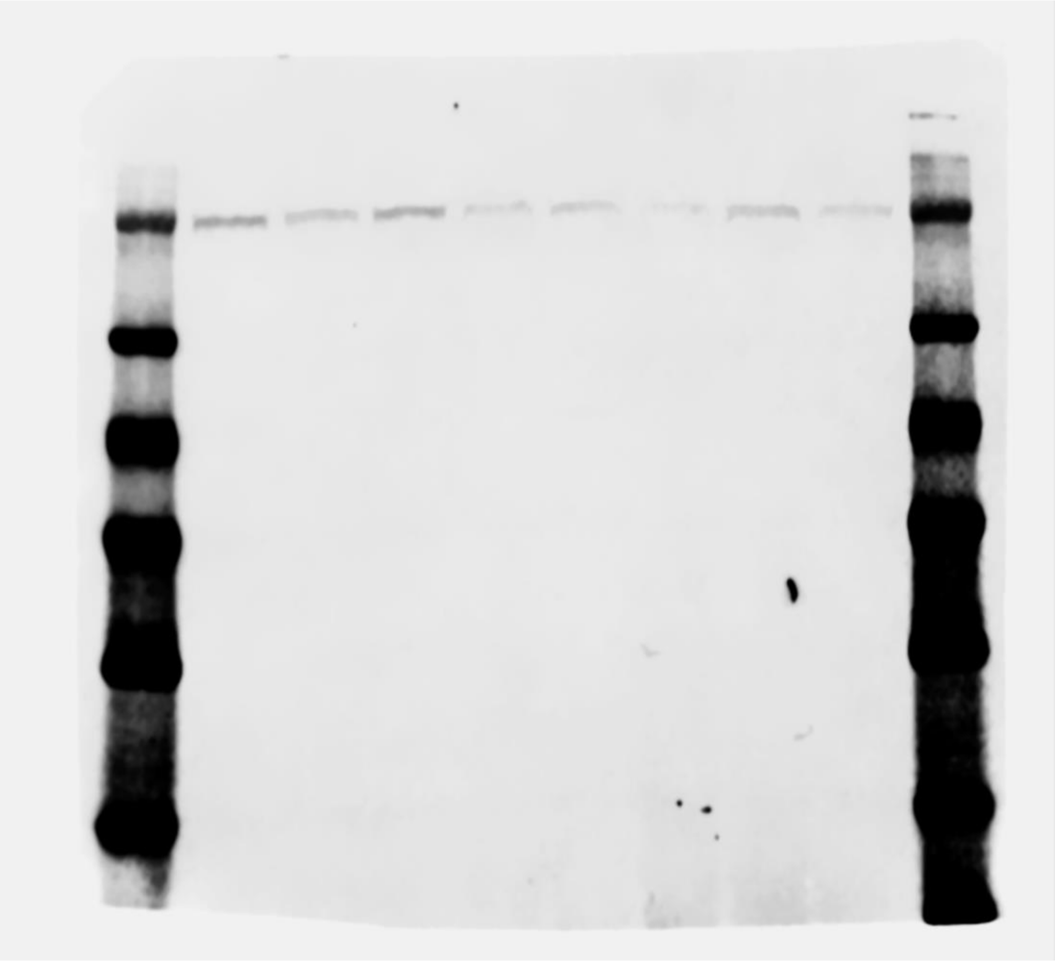

1B

Rel B

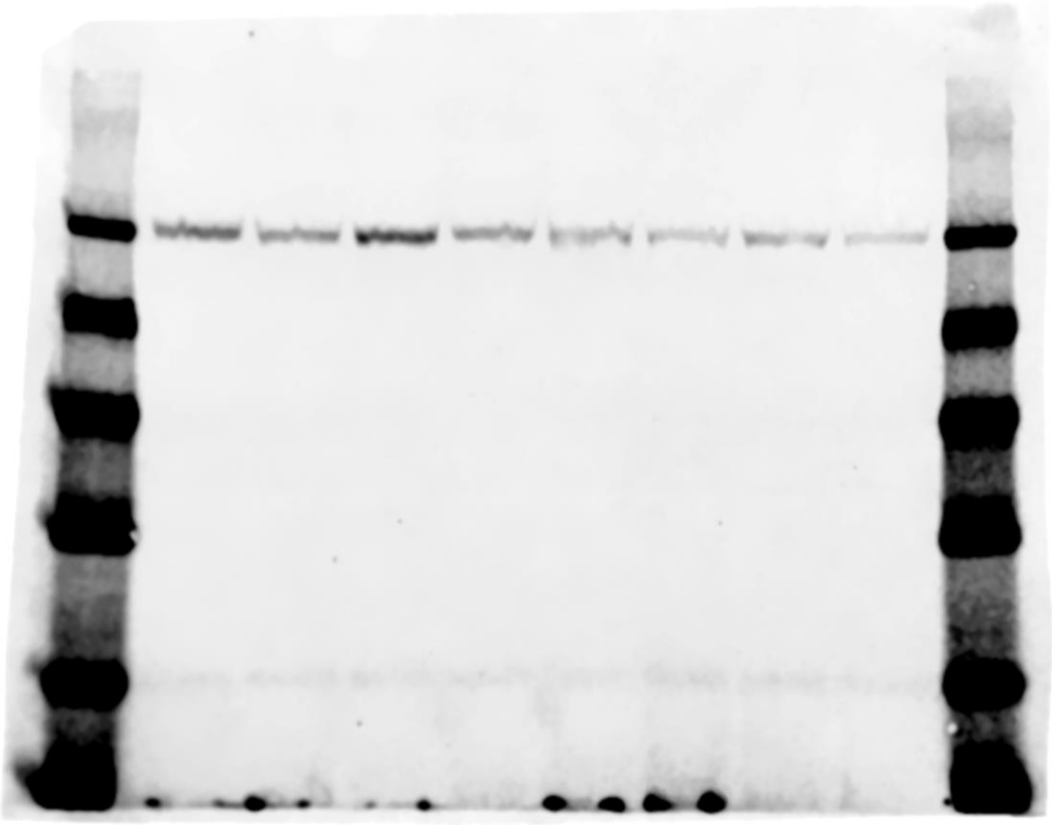

1B

cRel

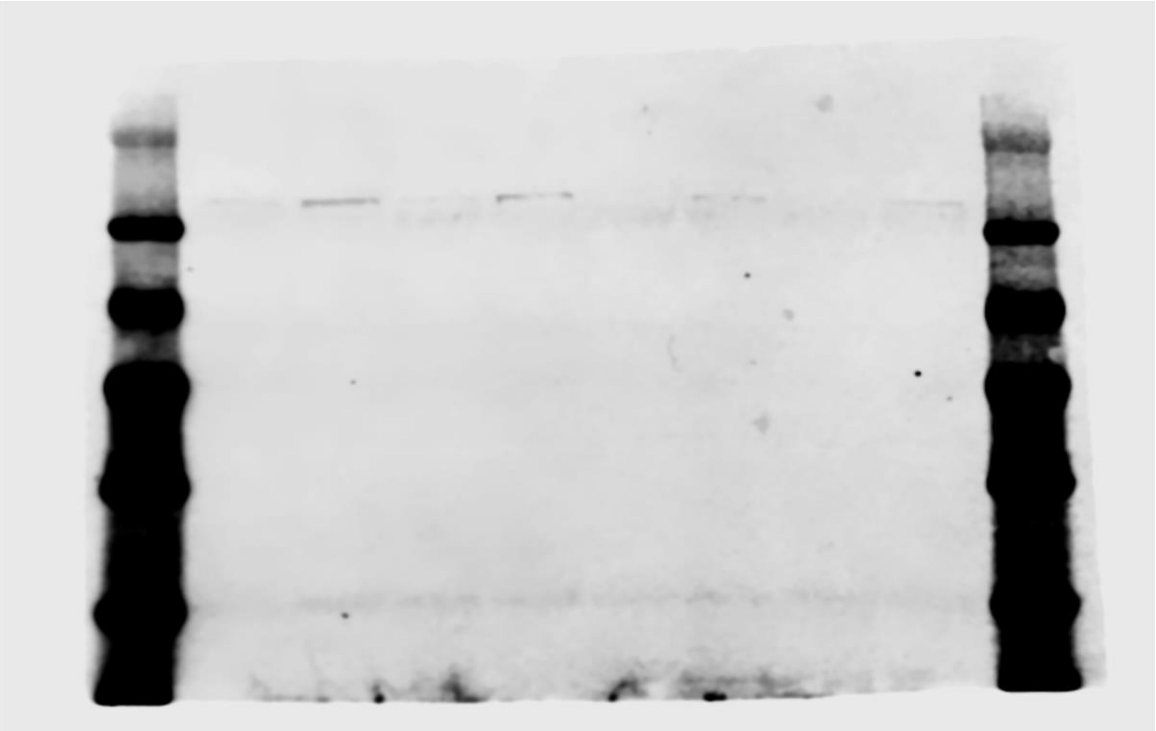

1B

pIKKα/β

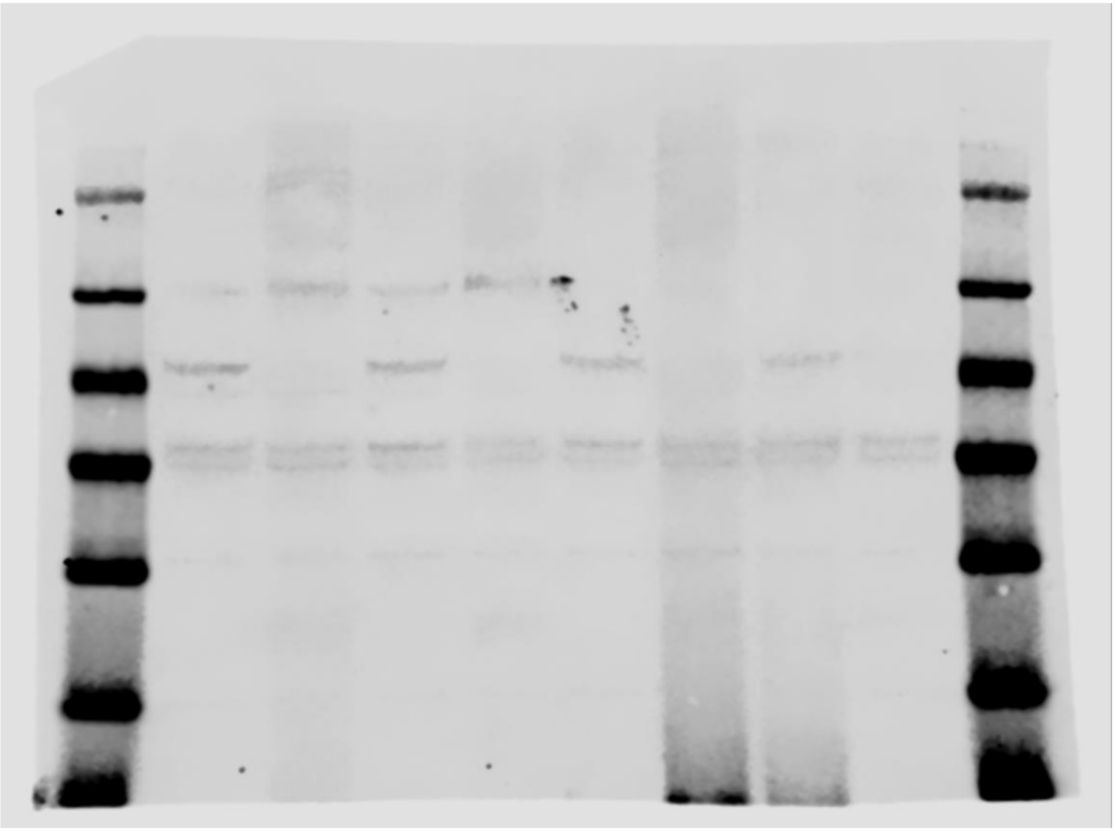

1B

IKKα

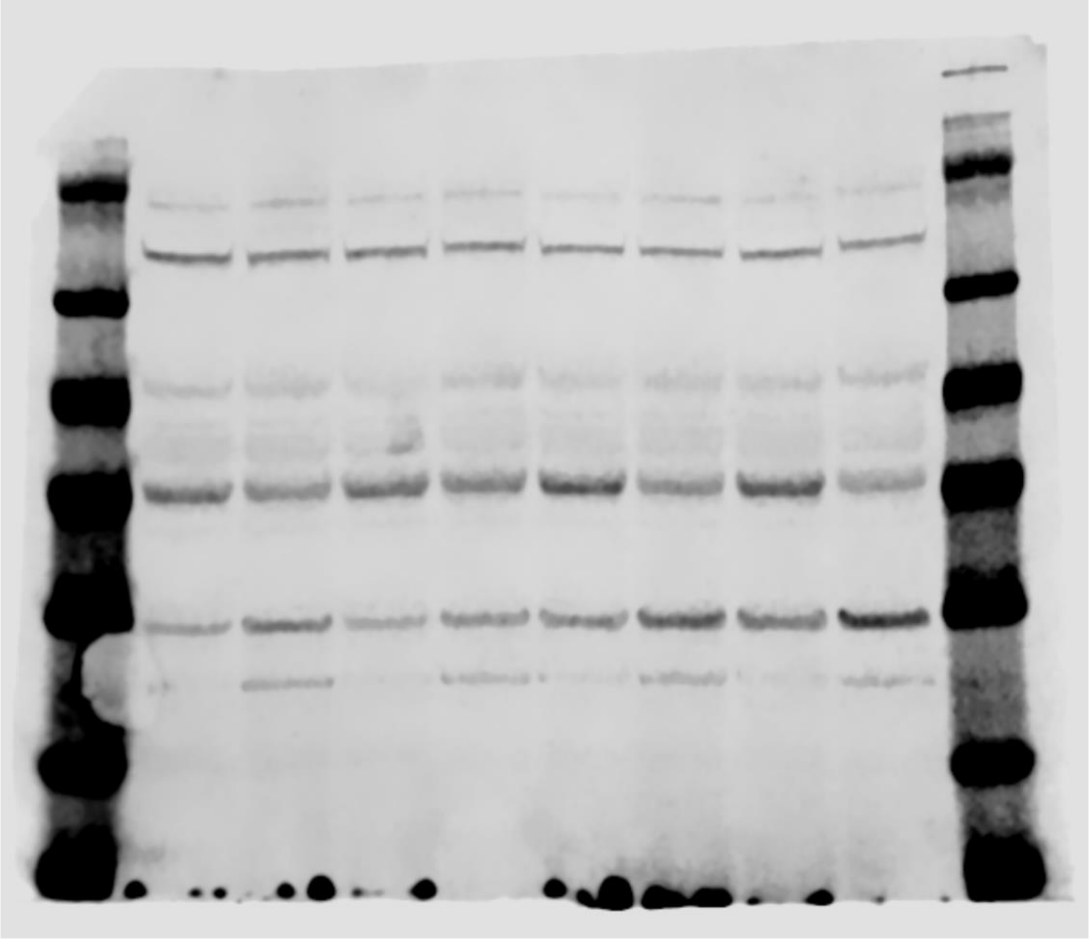

1B

IKKβ

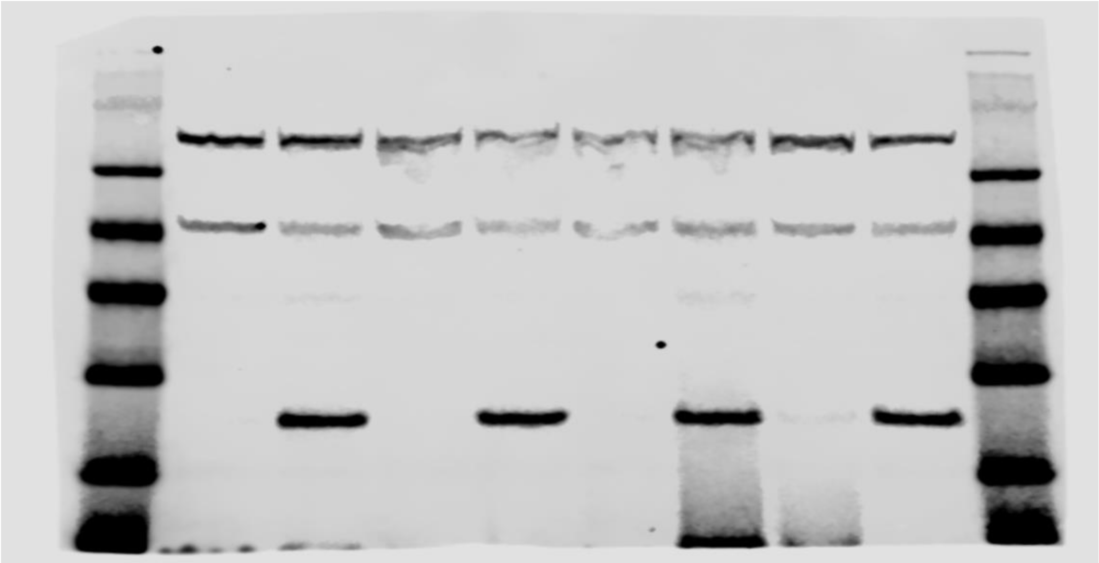

1B

pIKBα

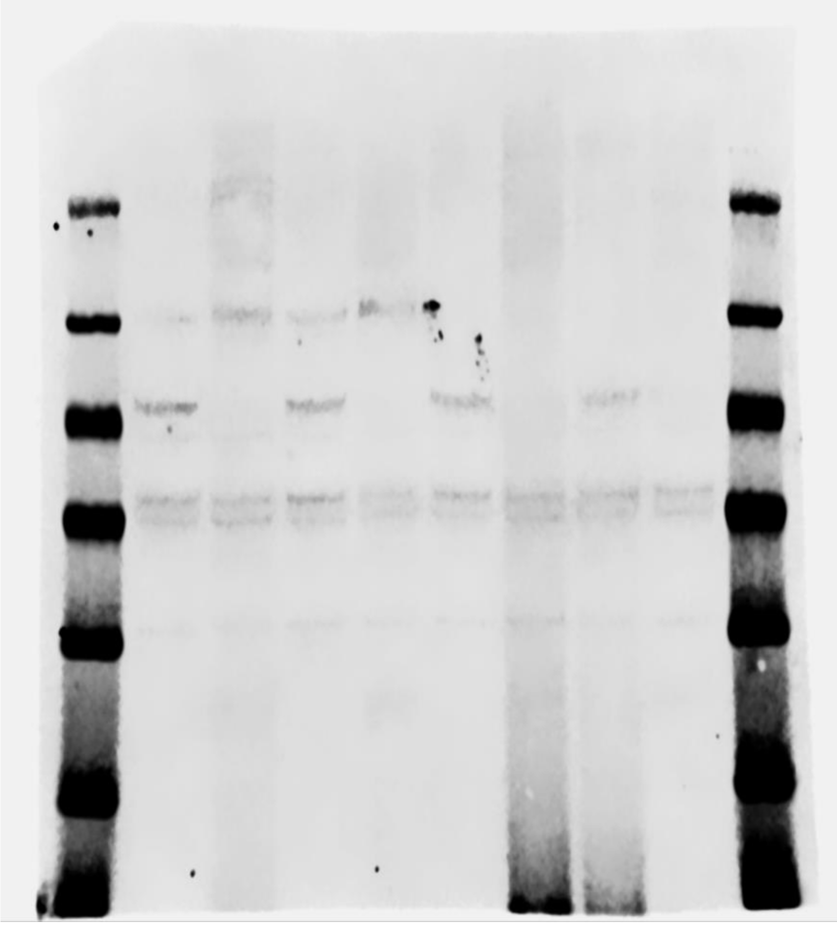

1B

IKBα

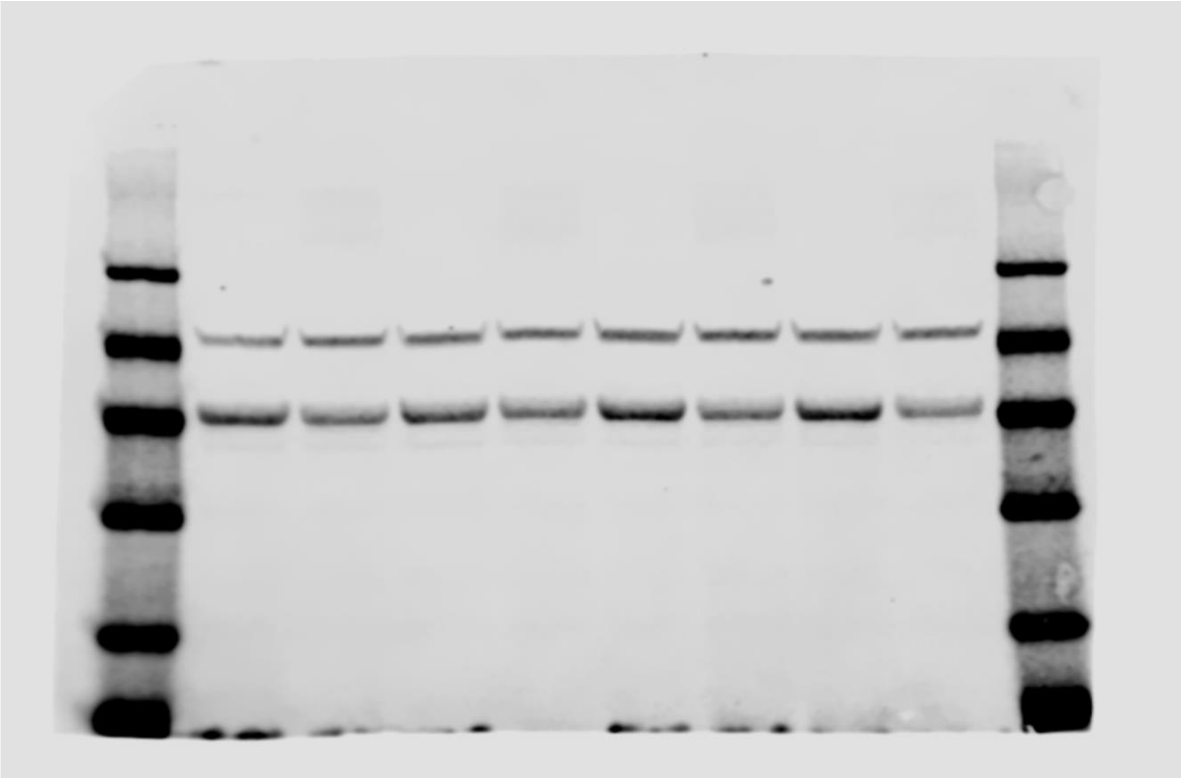

1B

pH2AX

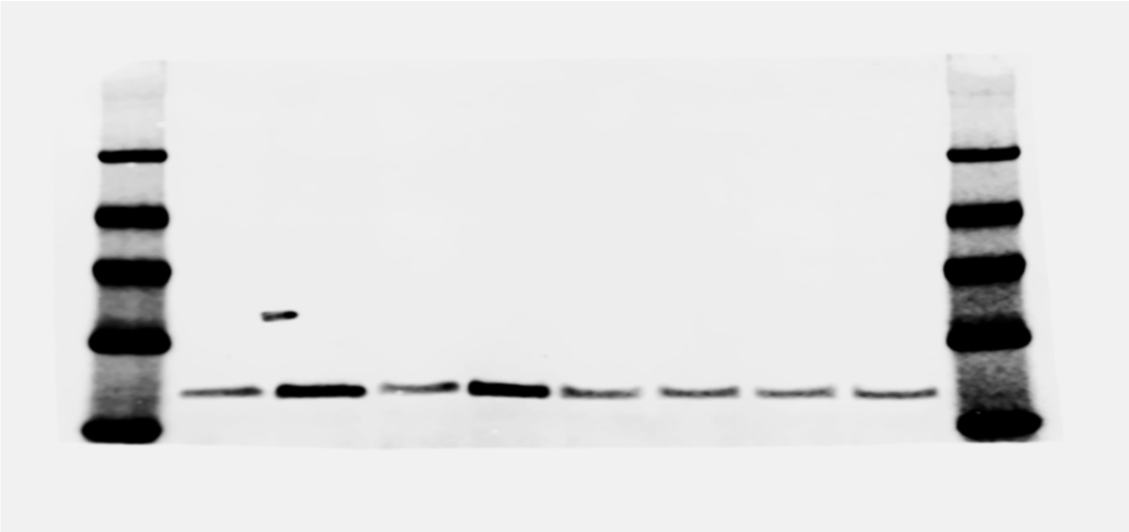

1B

H2AX

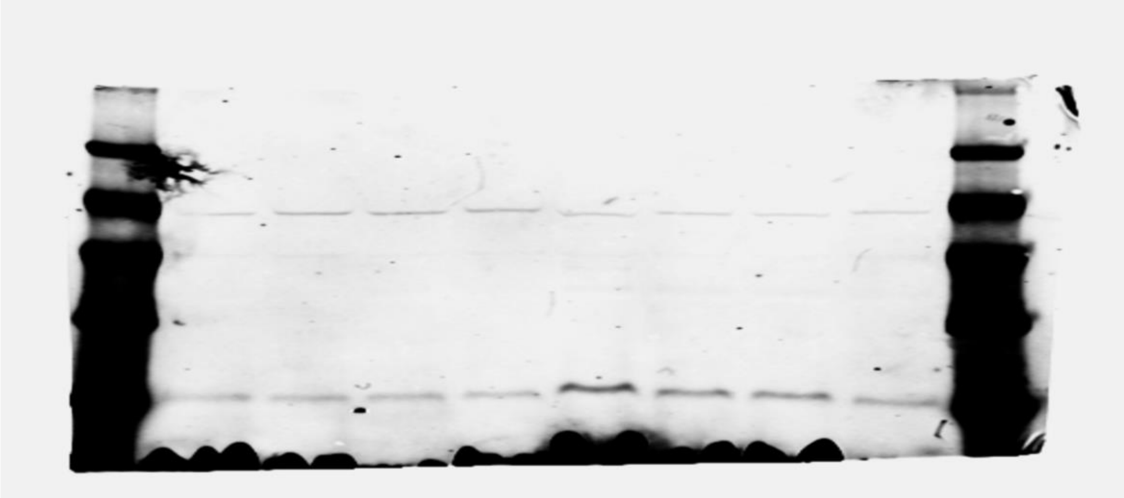

1B

ER alpha 66

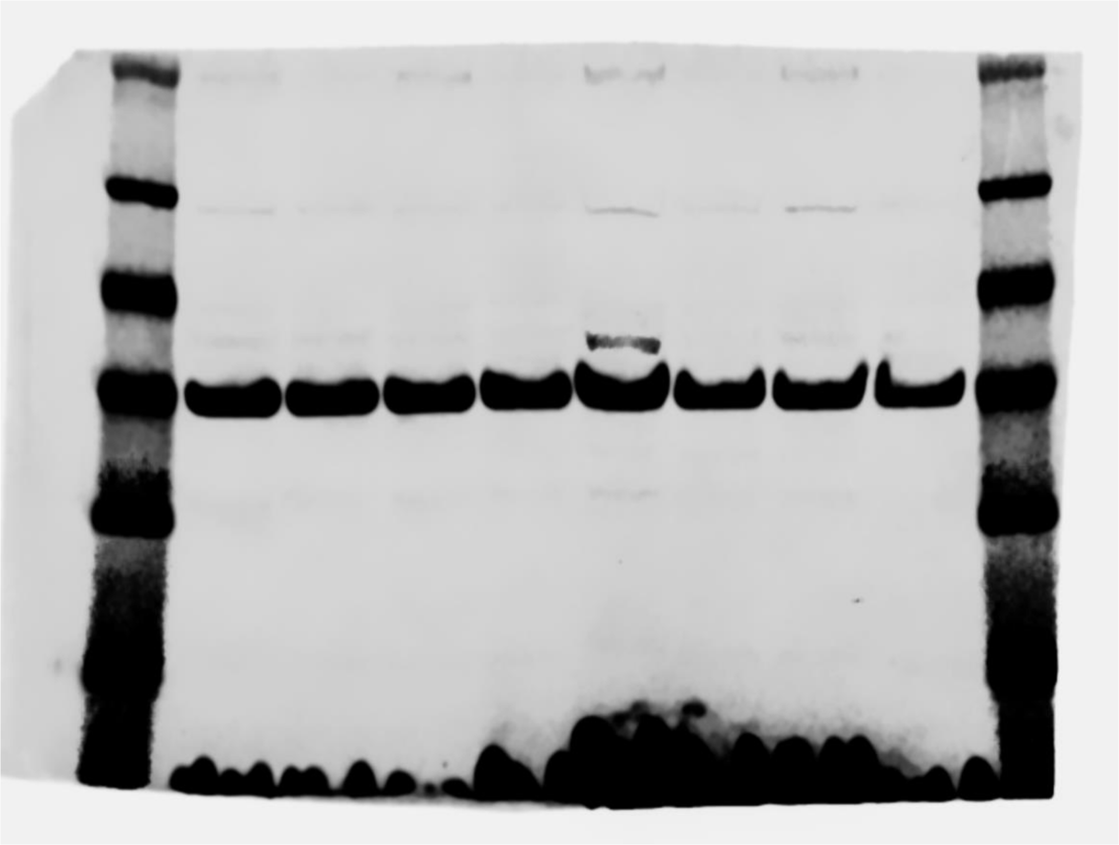

1B

RRM2

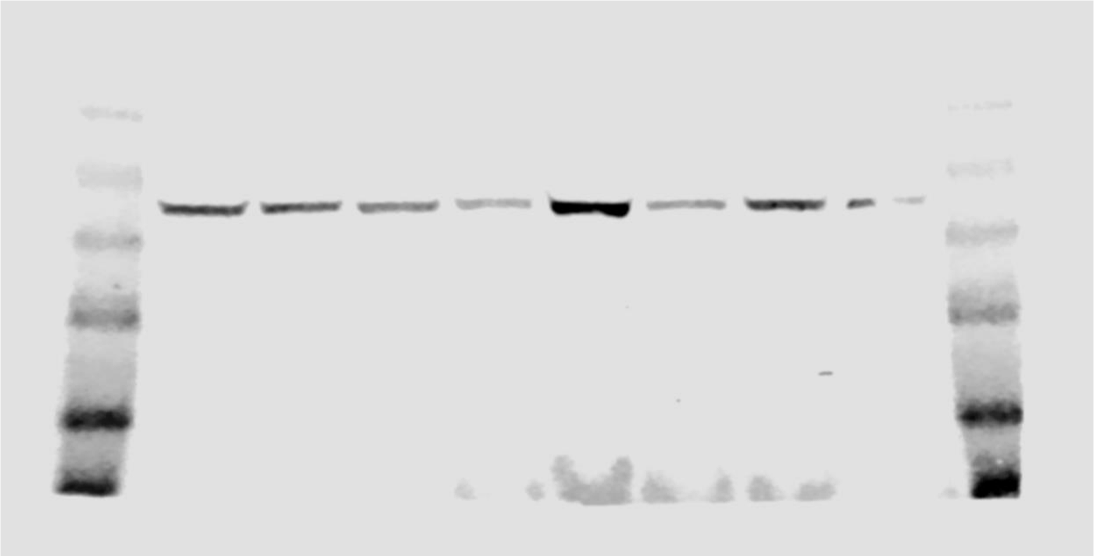

1B

GAPDH

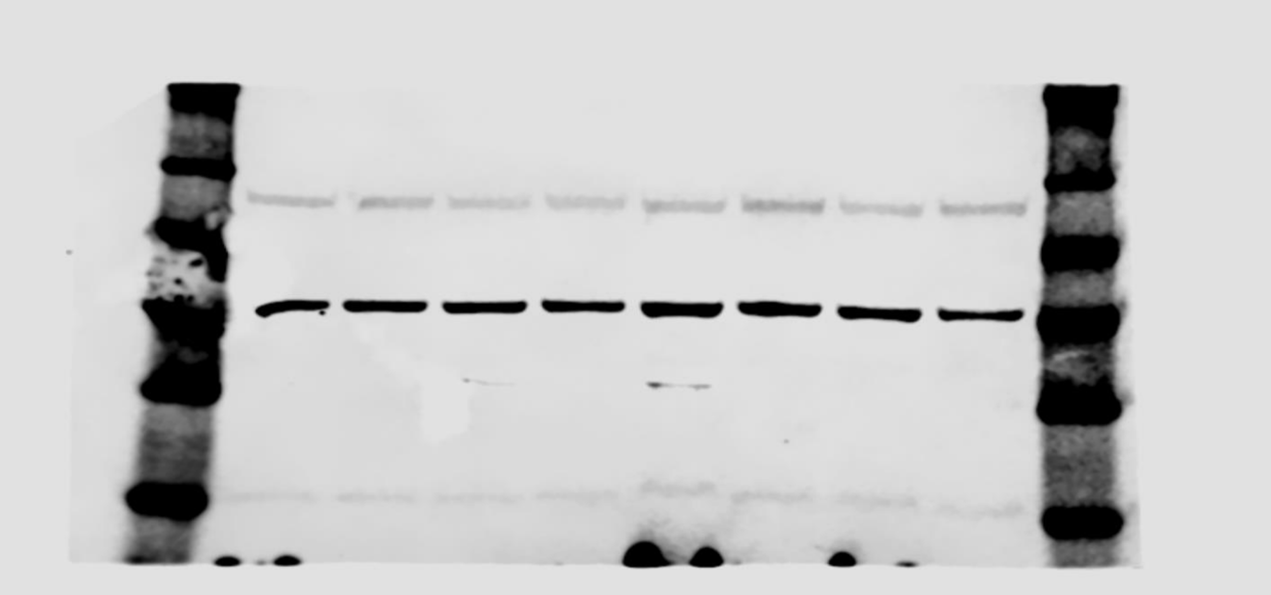

2A

Cyclin D1

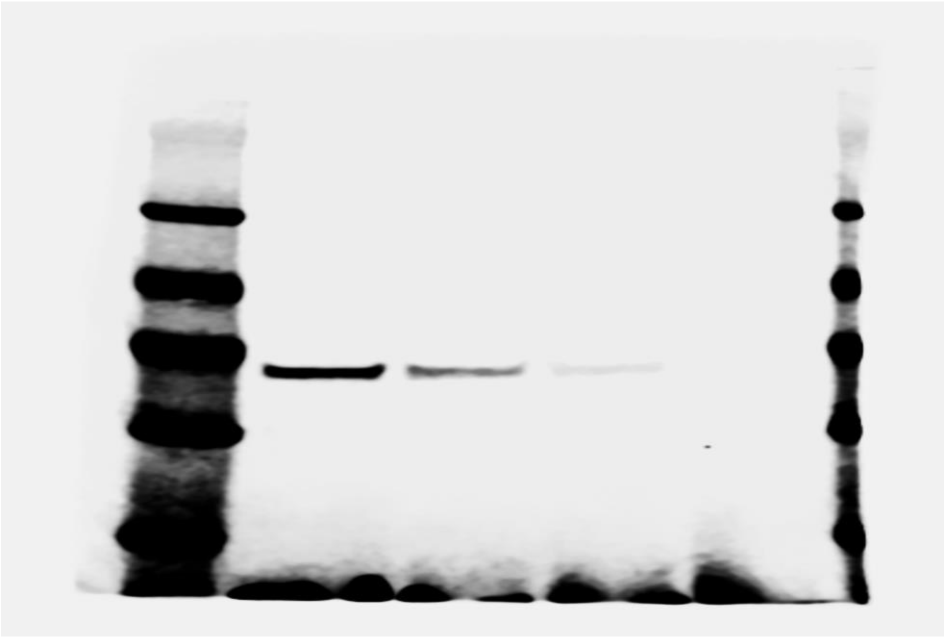

2B

pRb S807

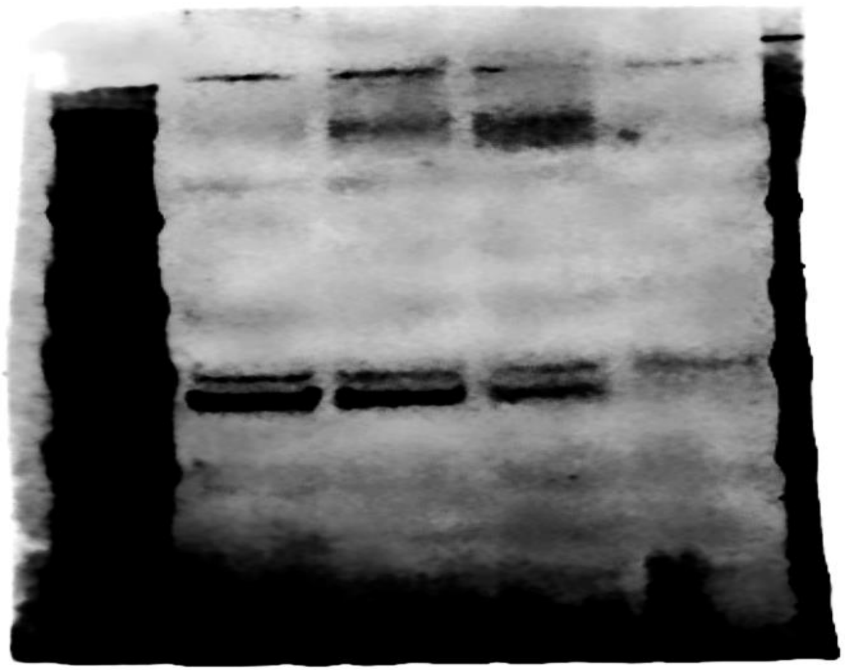

2B

Rb

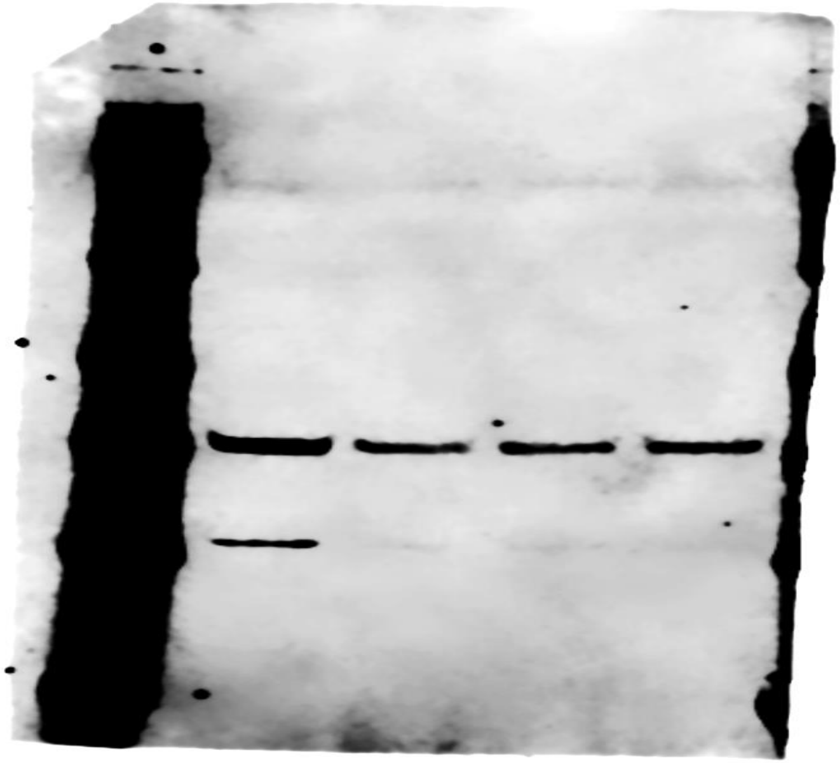

2B

Akt

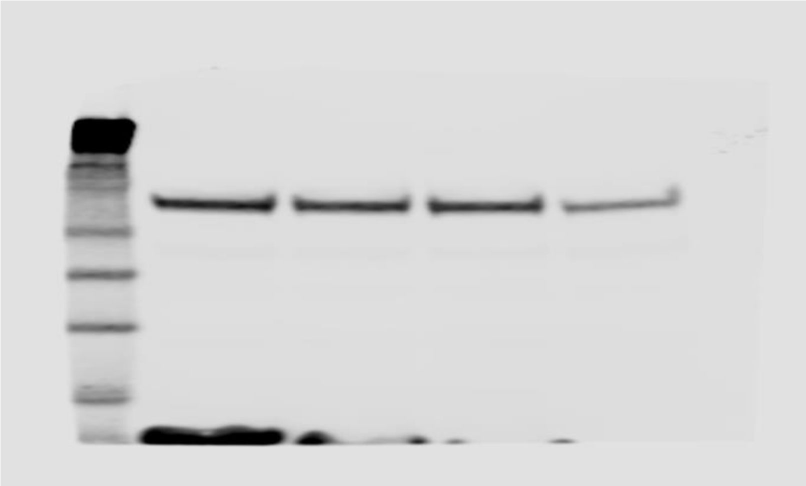

2B

p21

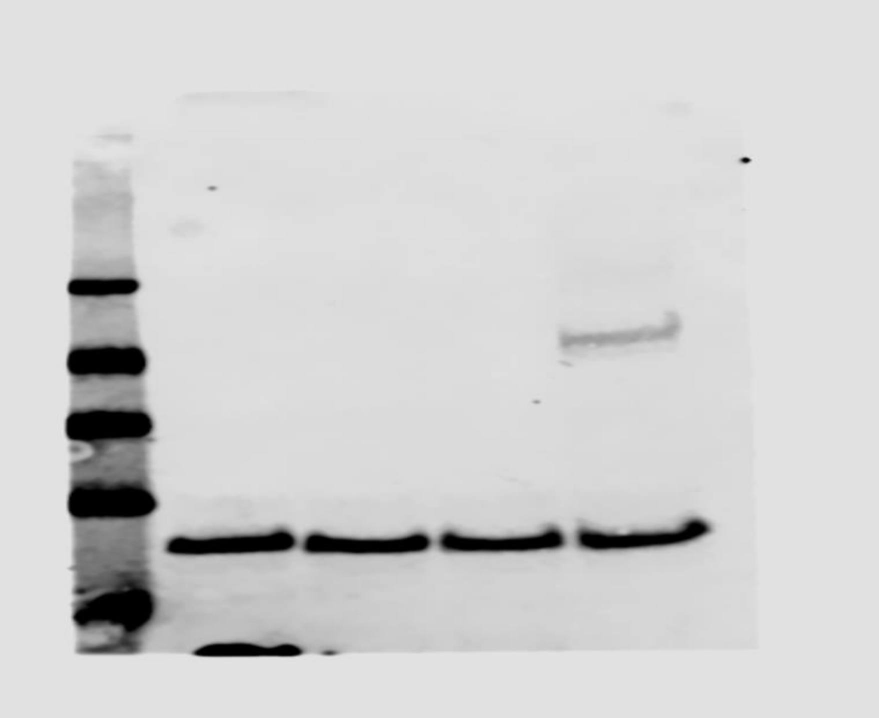

2B

IKBα

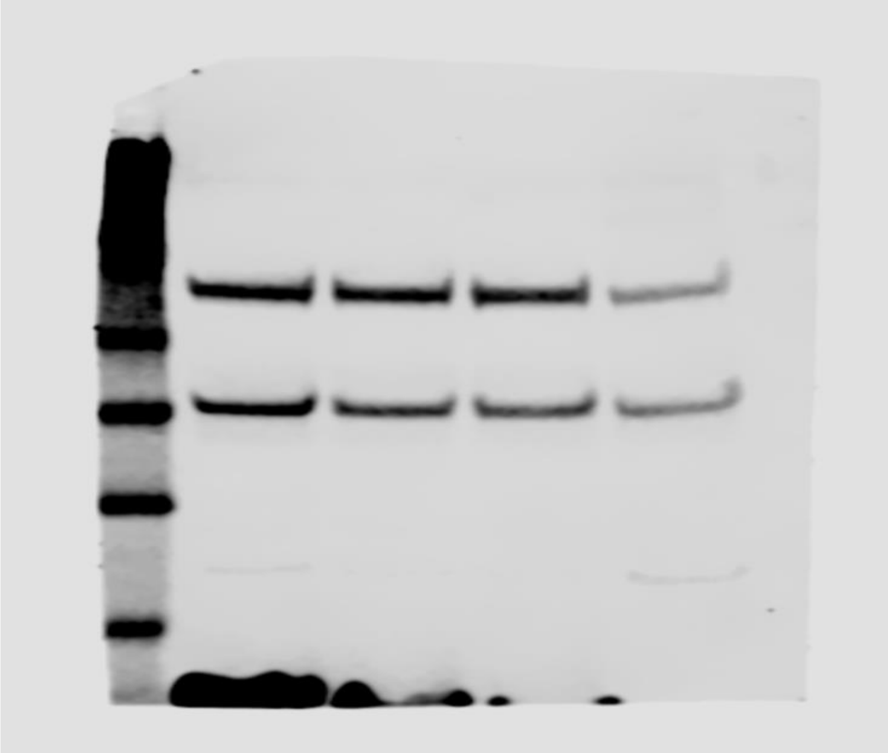

2B

RelB

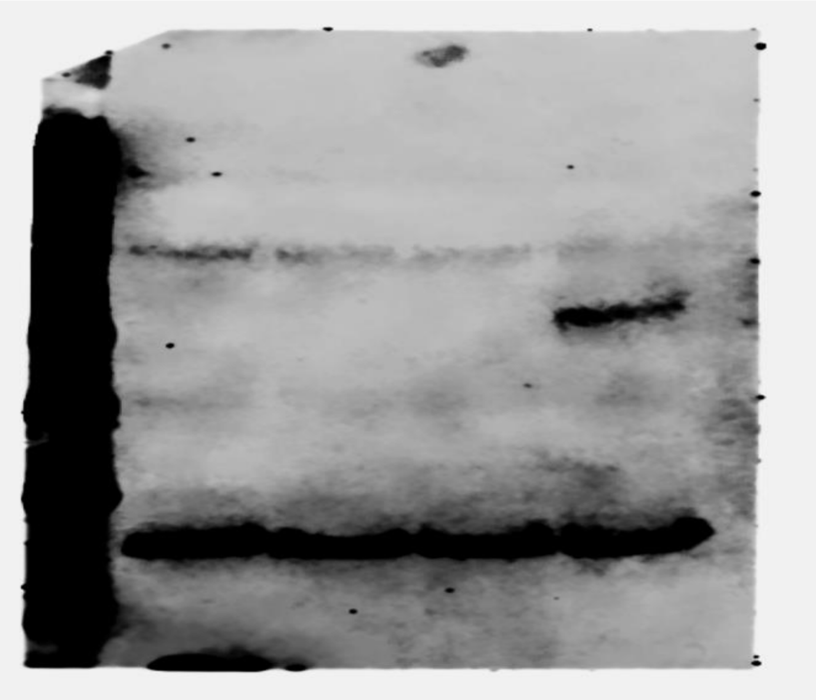

2B

pH2AX

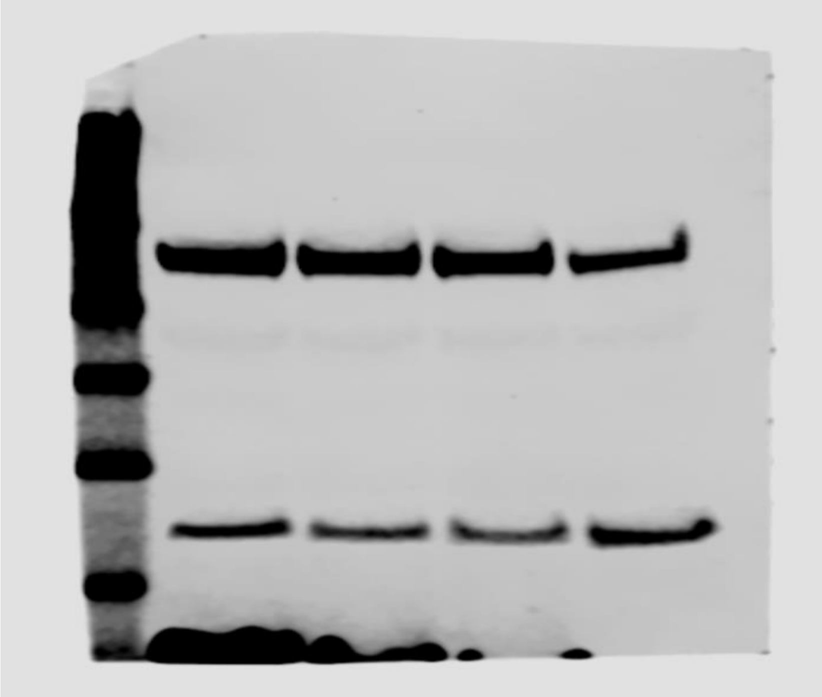

2B

H2AX

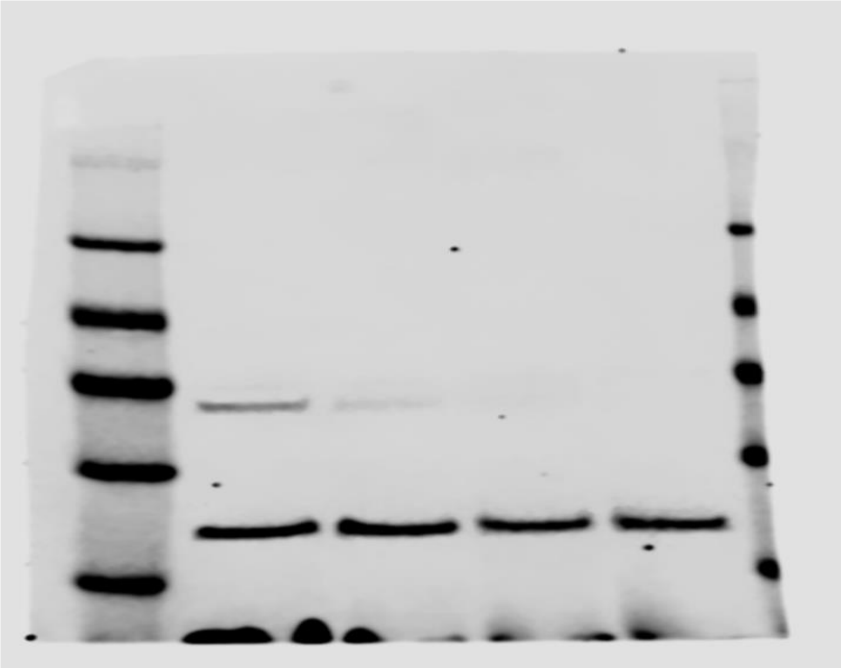

2B

RRM2

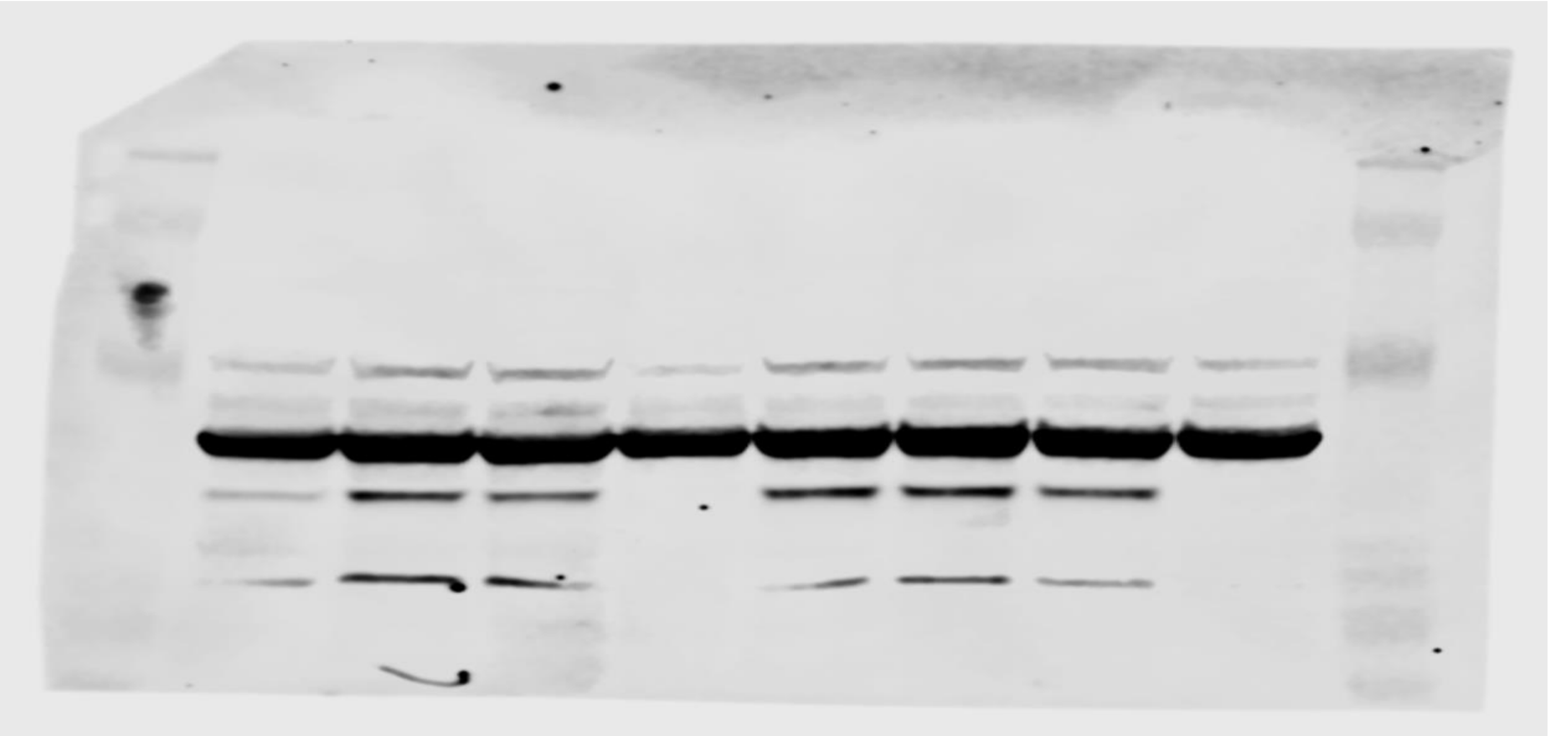

2B

GAPDH

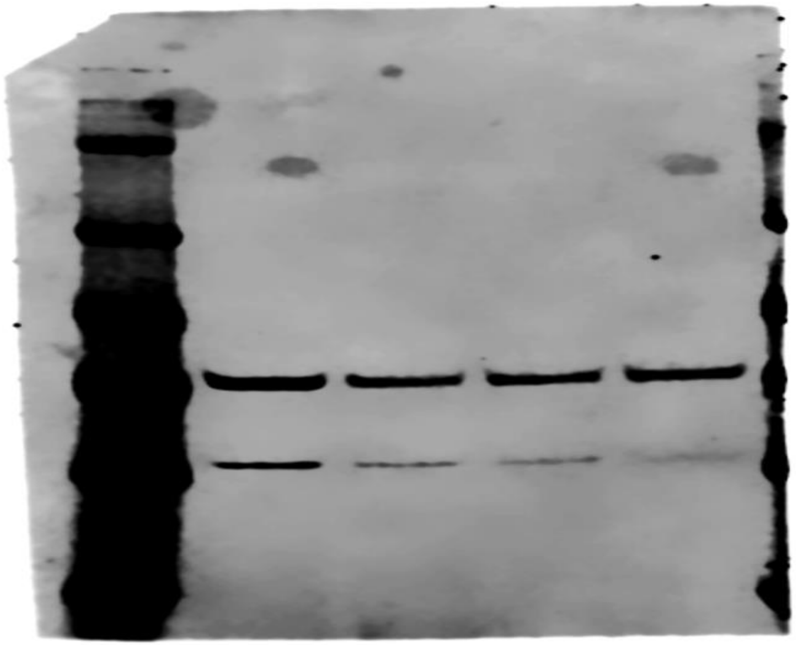

2B

Cyclin D1

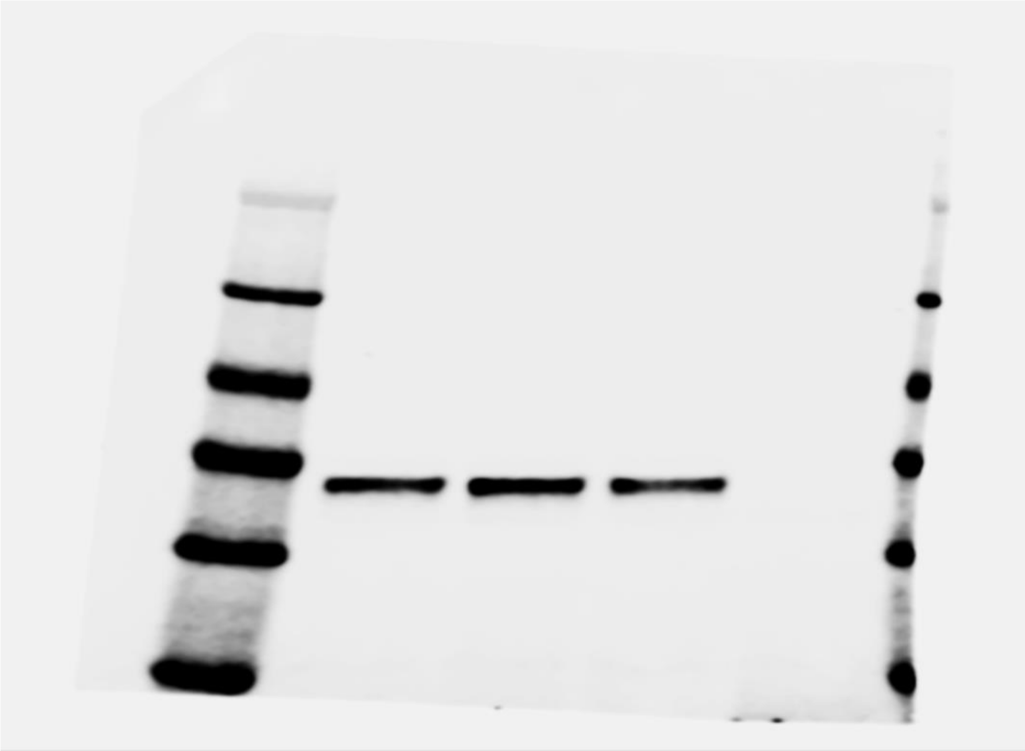

2B

pRb S807

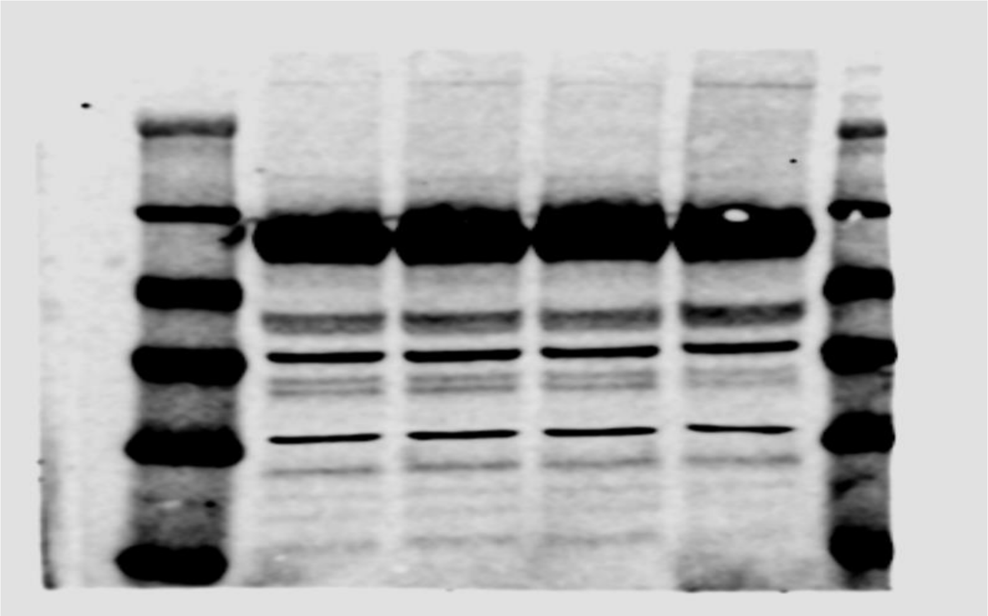

2B

Rb

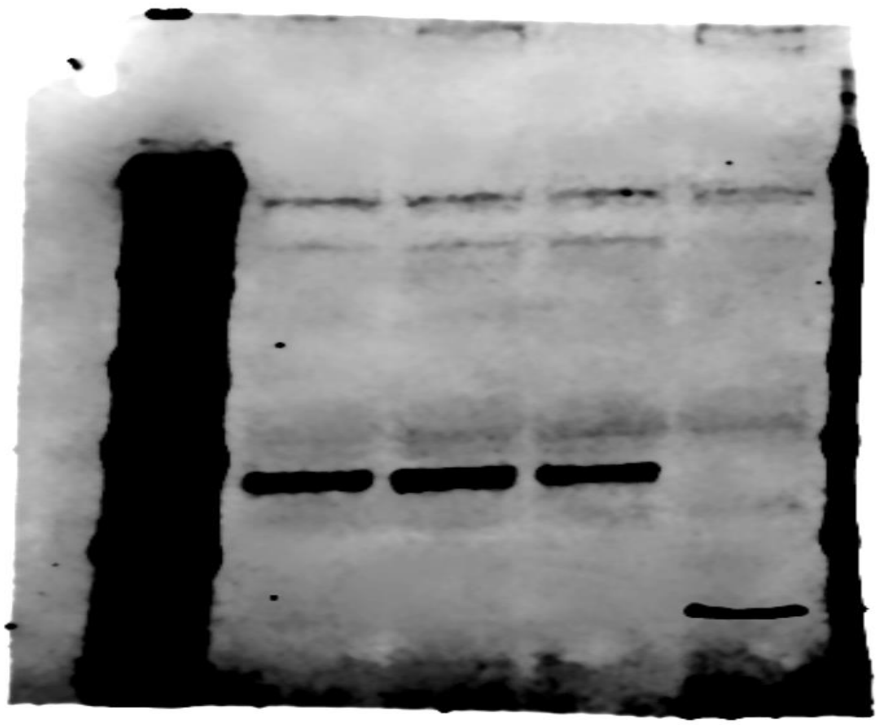

2B

Akt

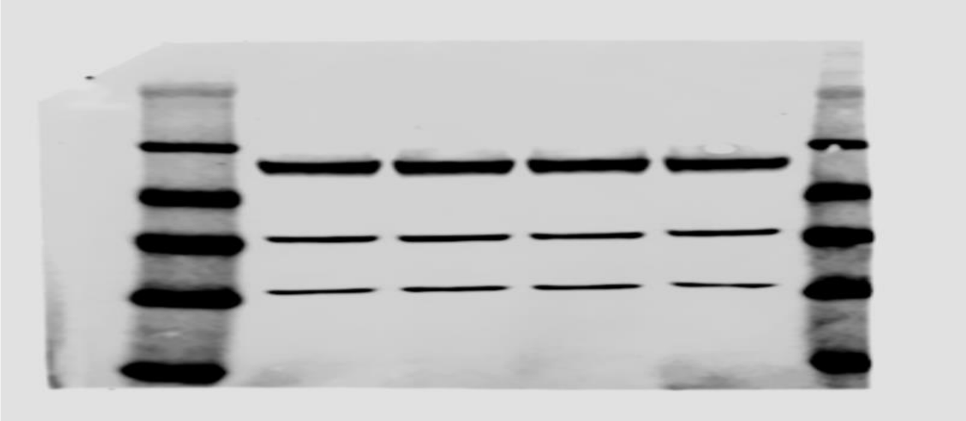

2B

p21

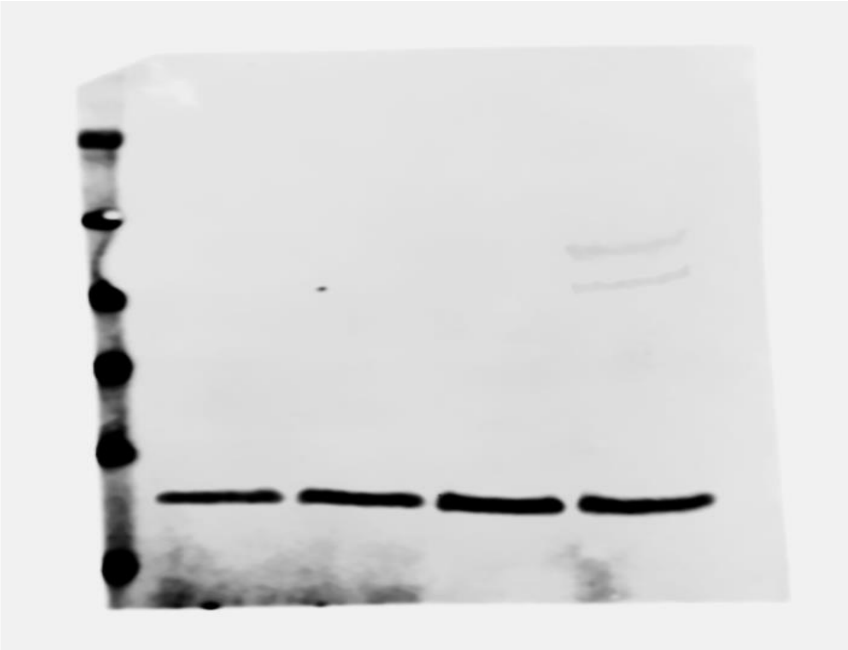

2B

IKBα

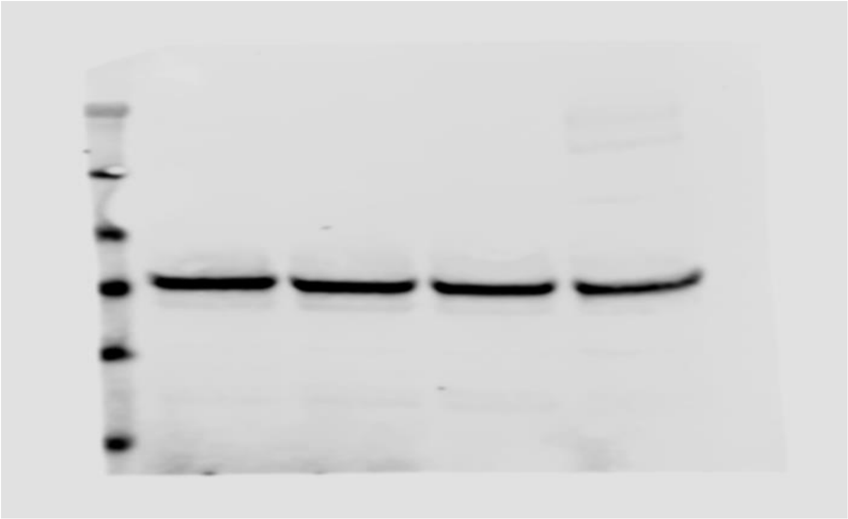

2B

RelB

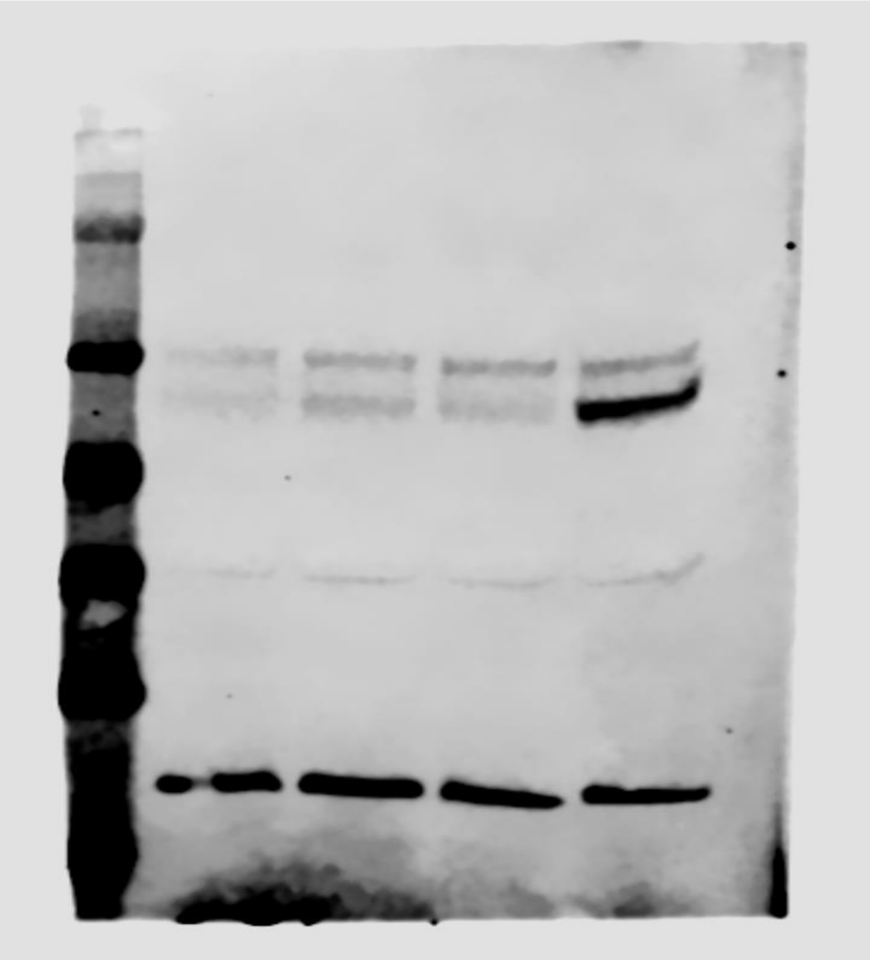

2B

pH2AX

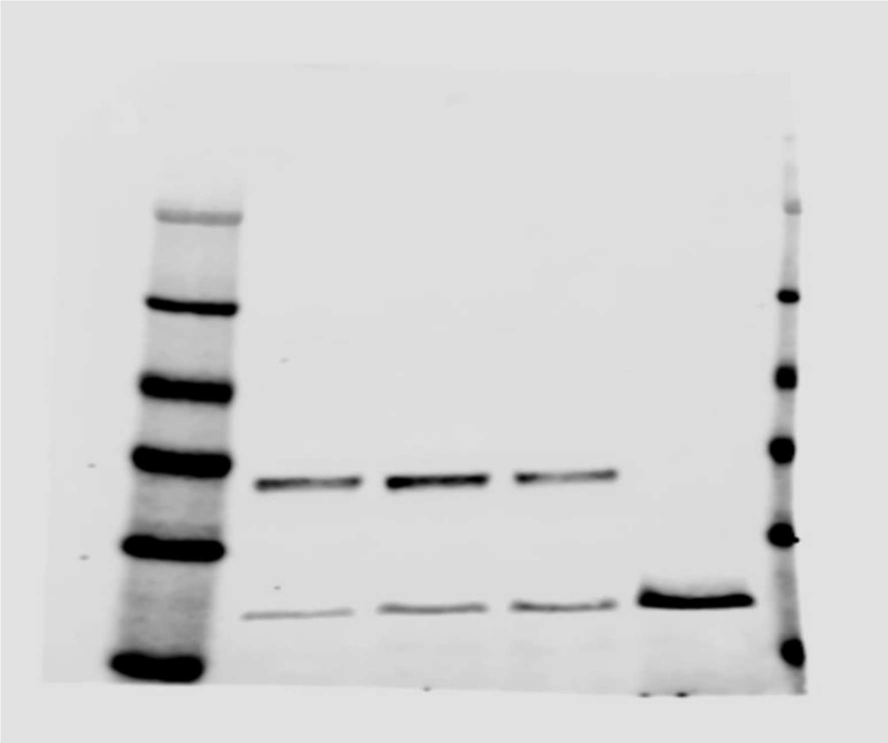

2B

H2AX

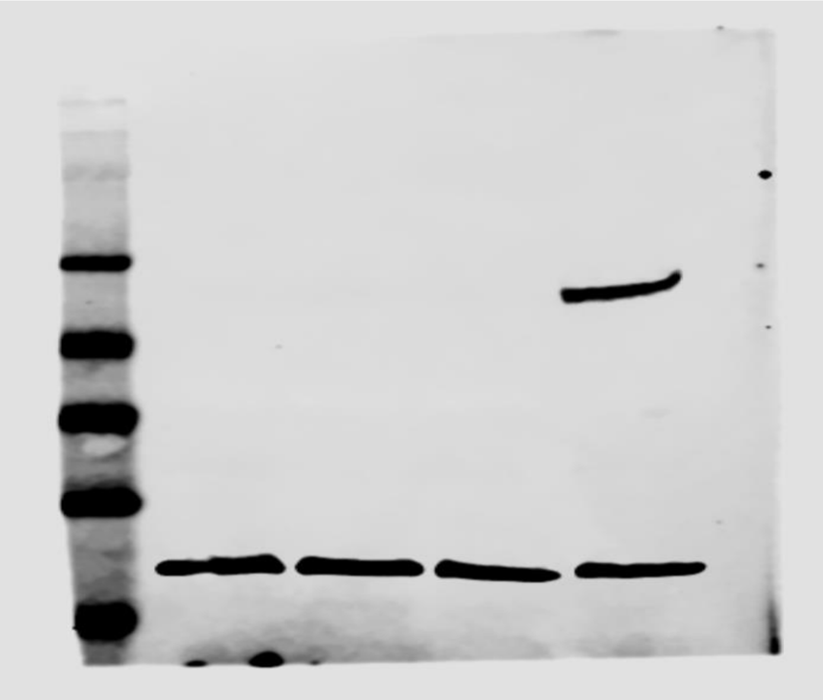

2B RRM2

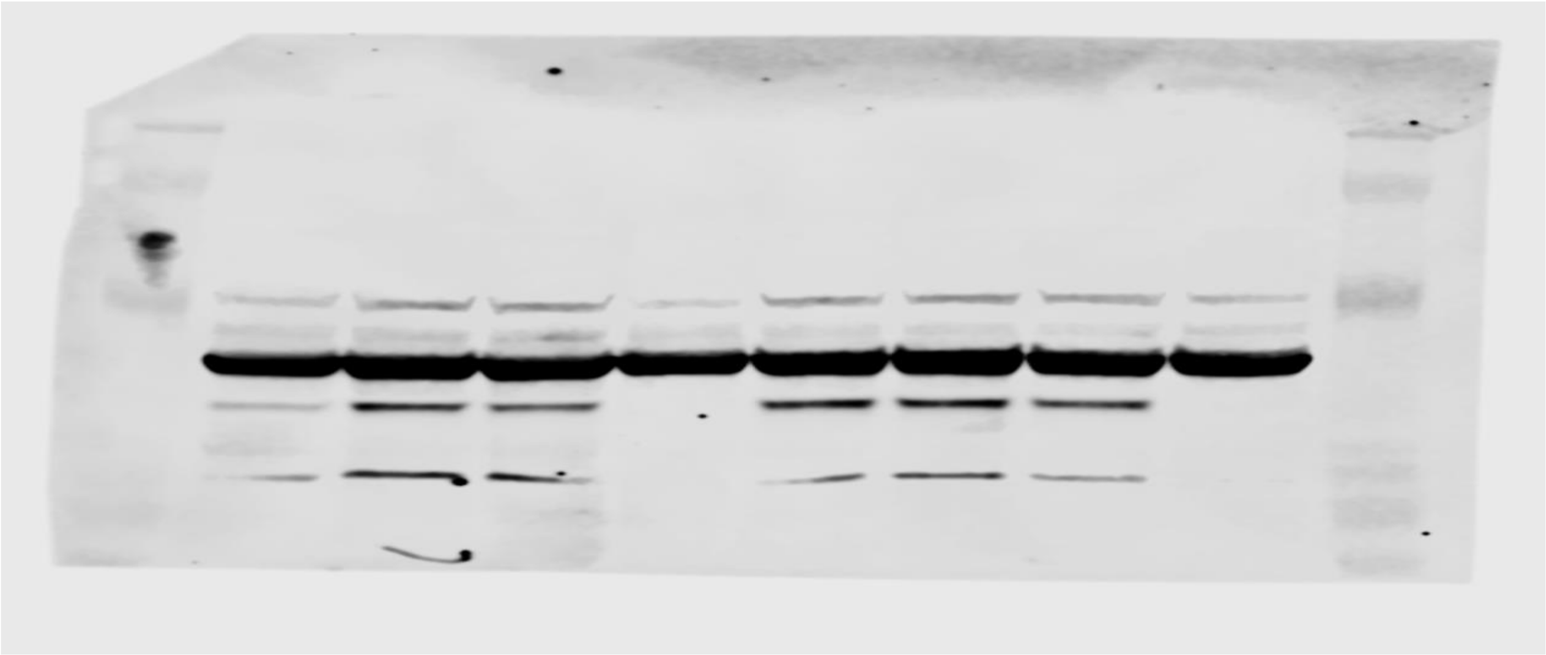

2B GAPDH

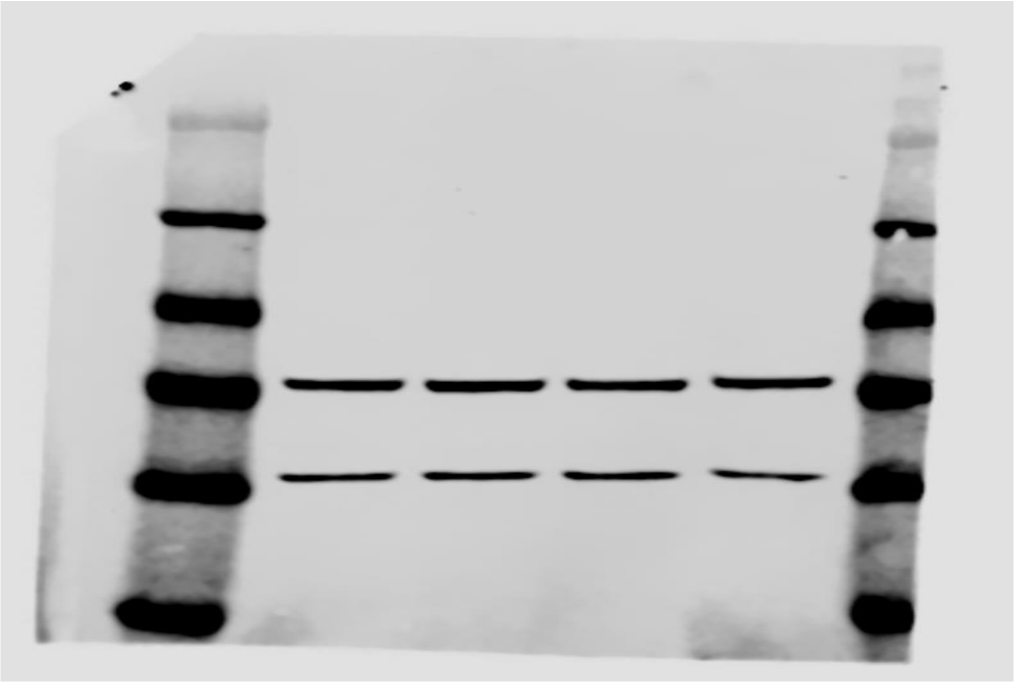

2B

Cyclin D1

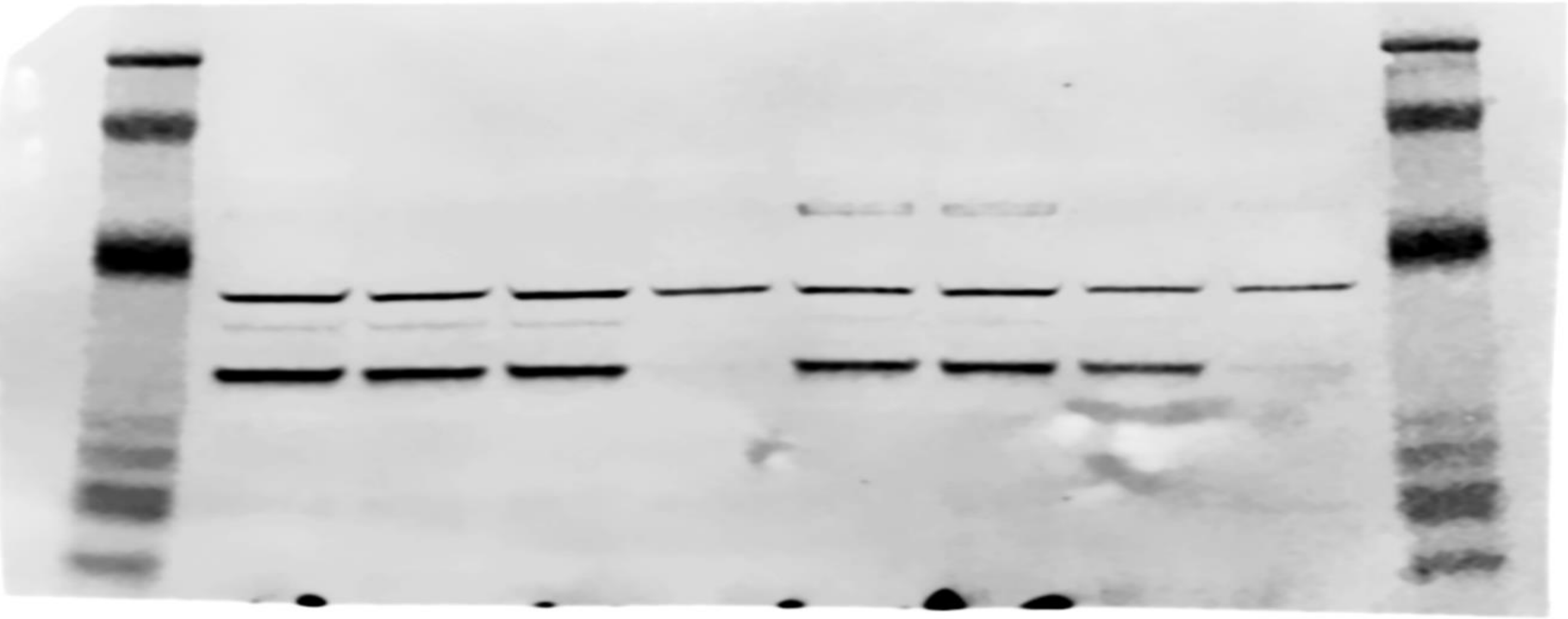

2B

Cyclin E2

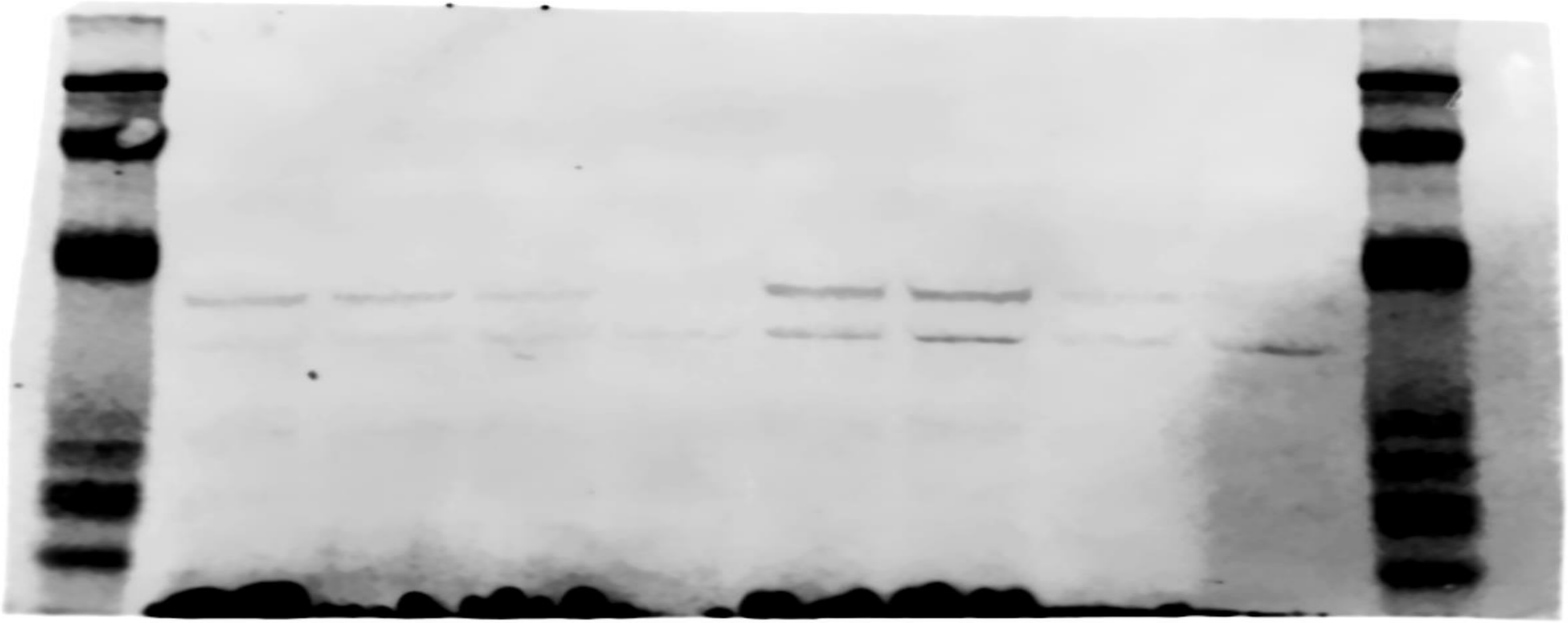

2B

pRb

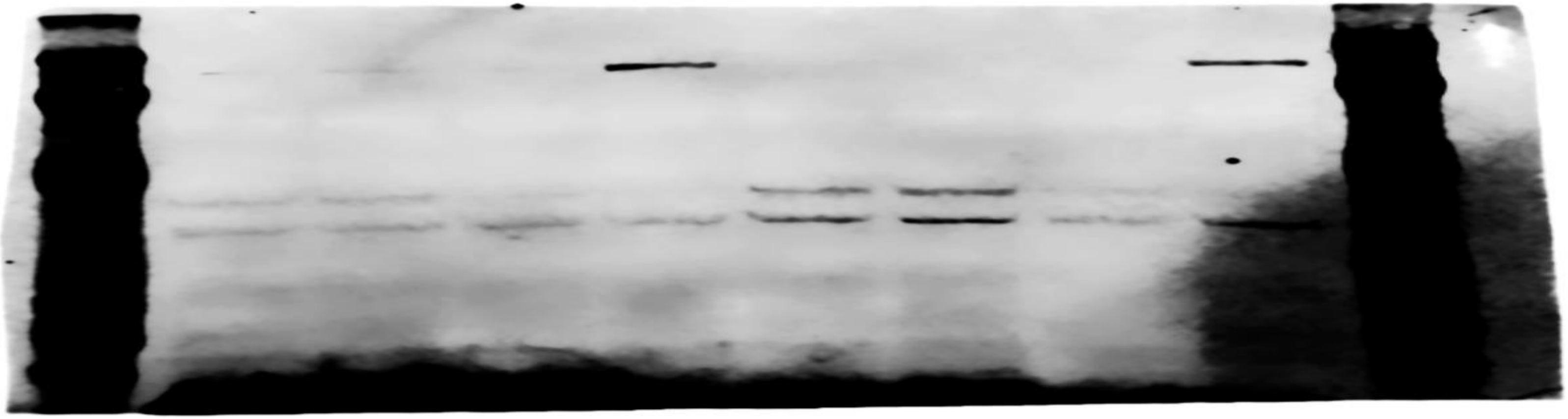

2B Rb

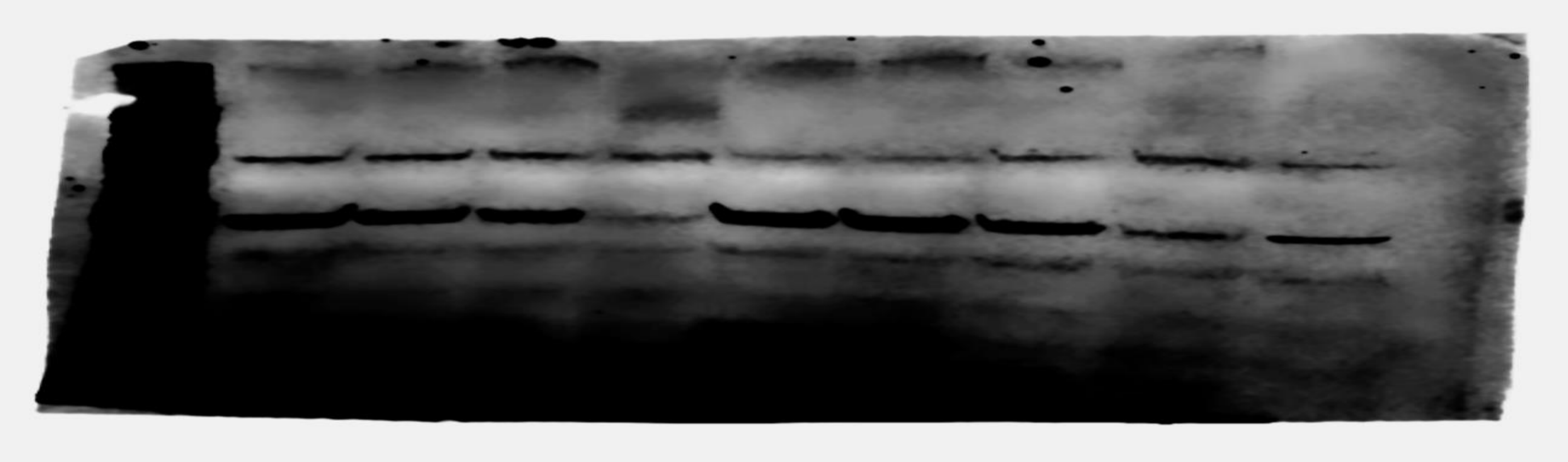

2B

Akt

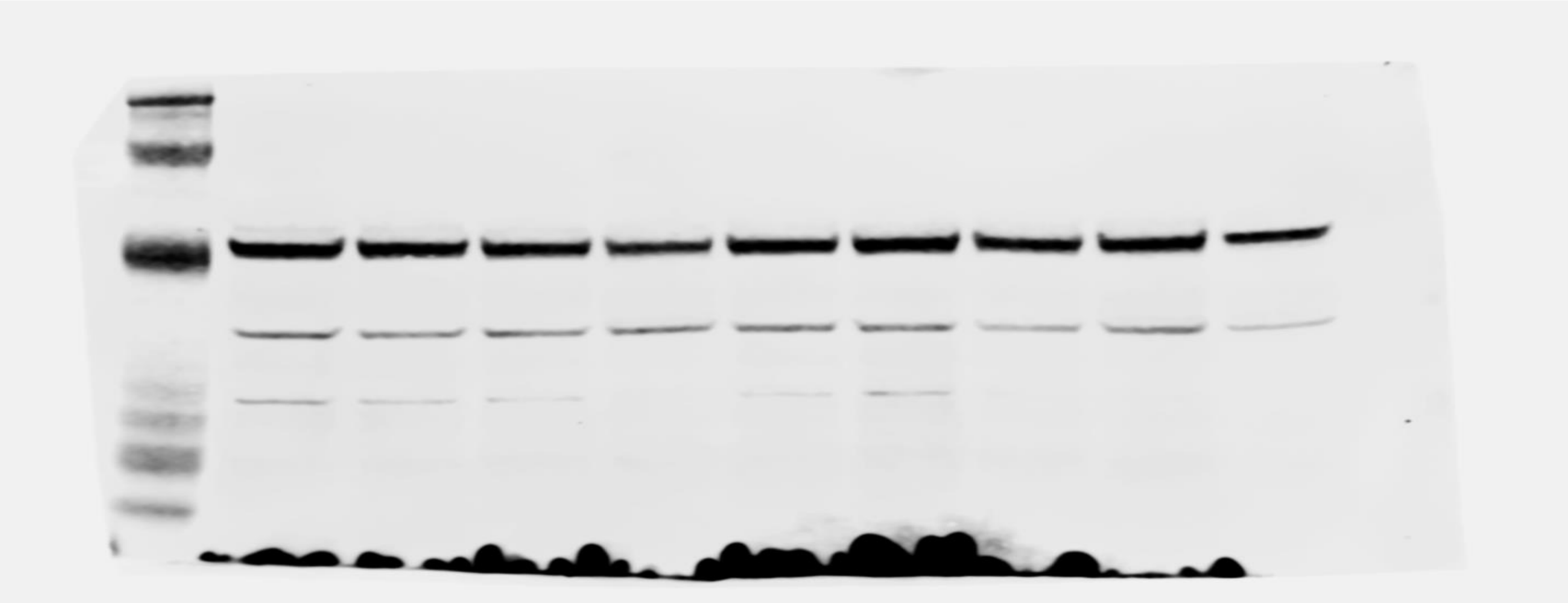

2B

p100

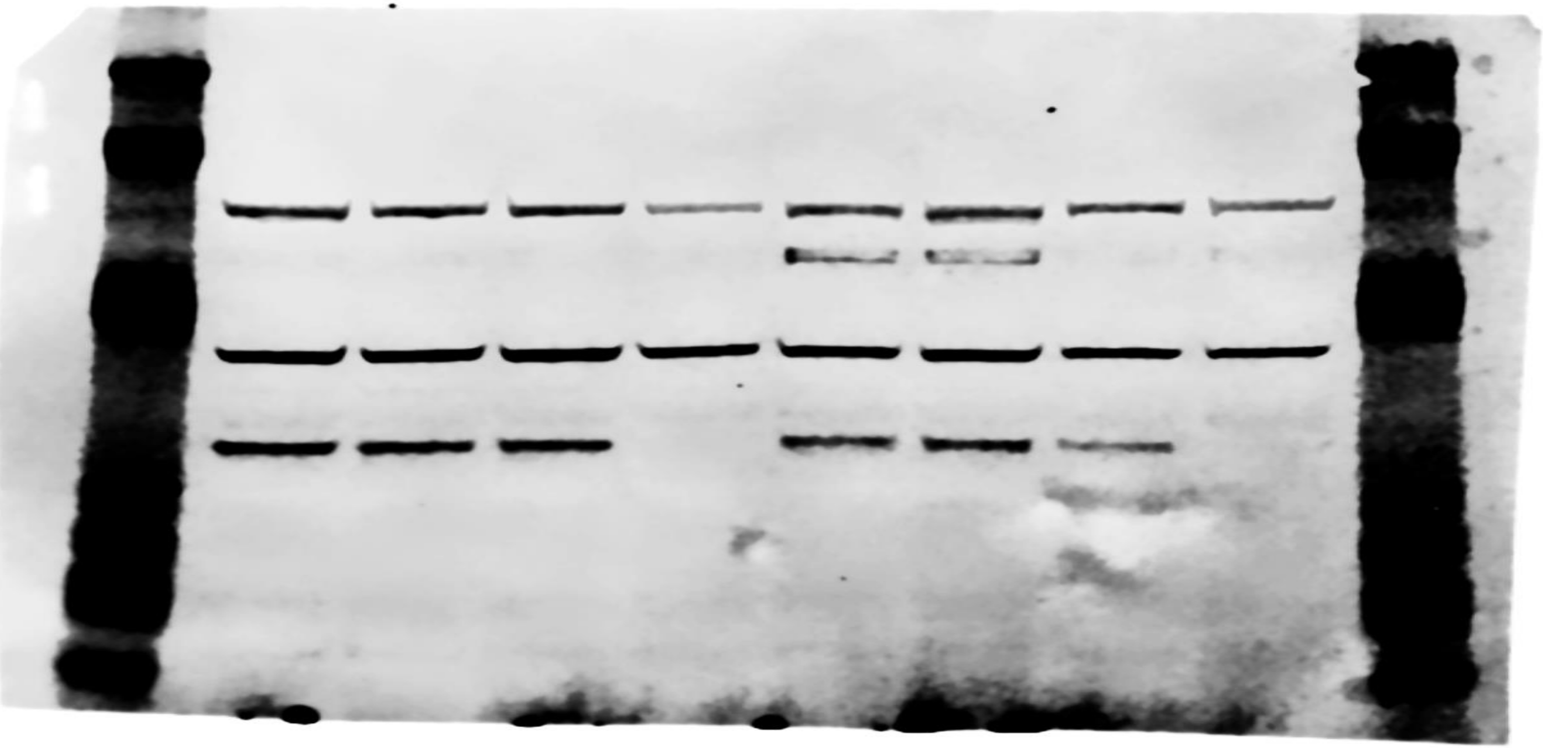

2B

cRel

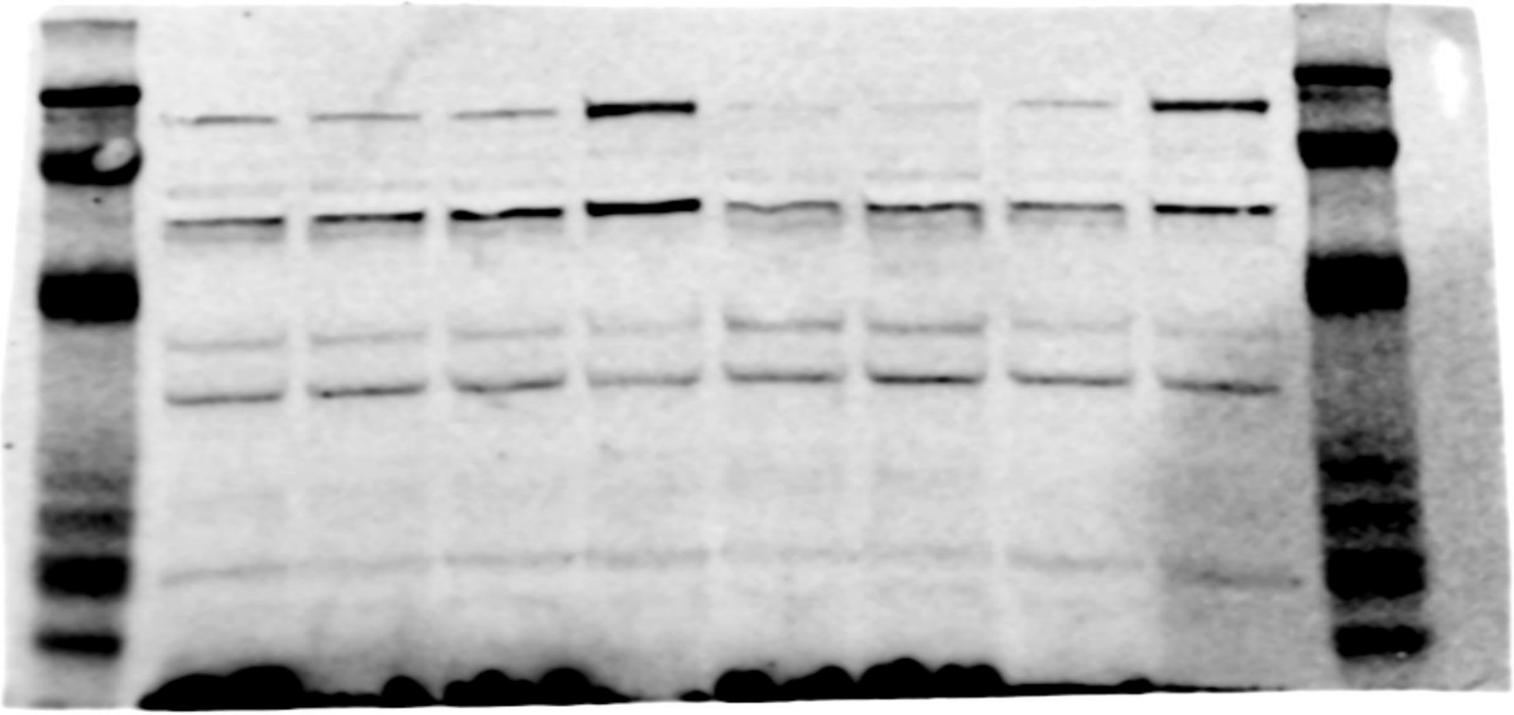

2B

Mut p53

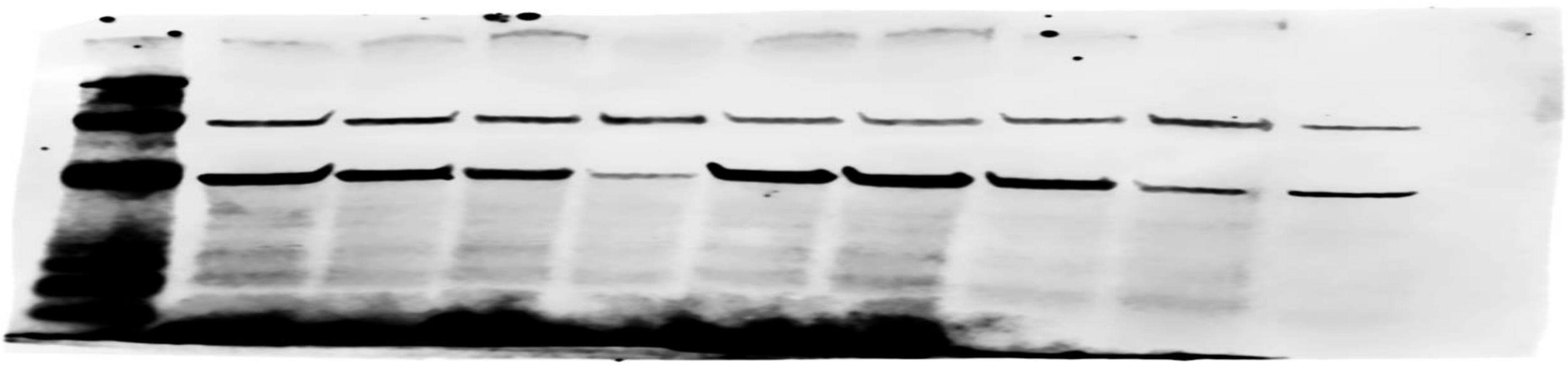

2B RRM2

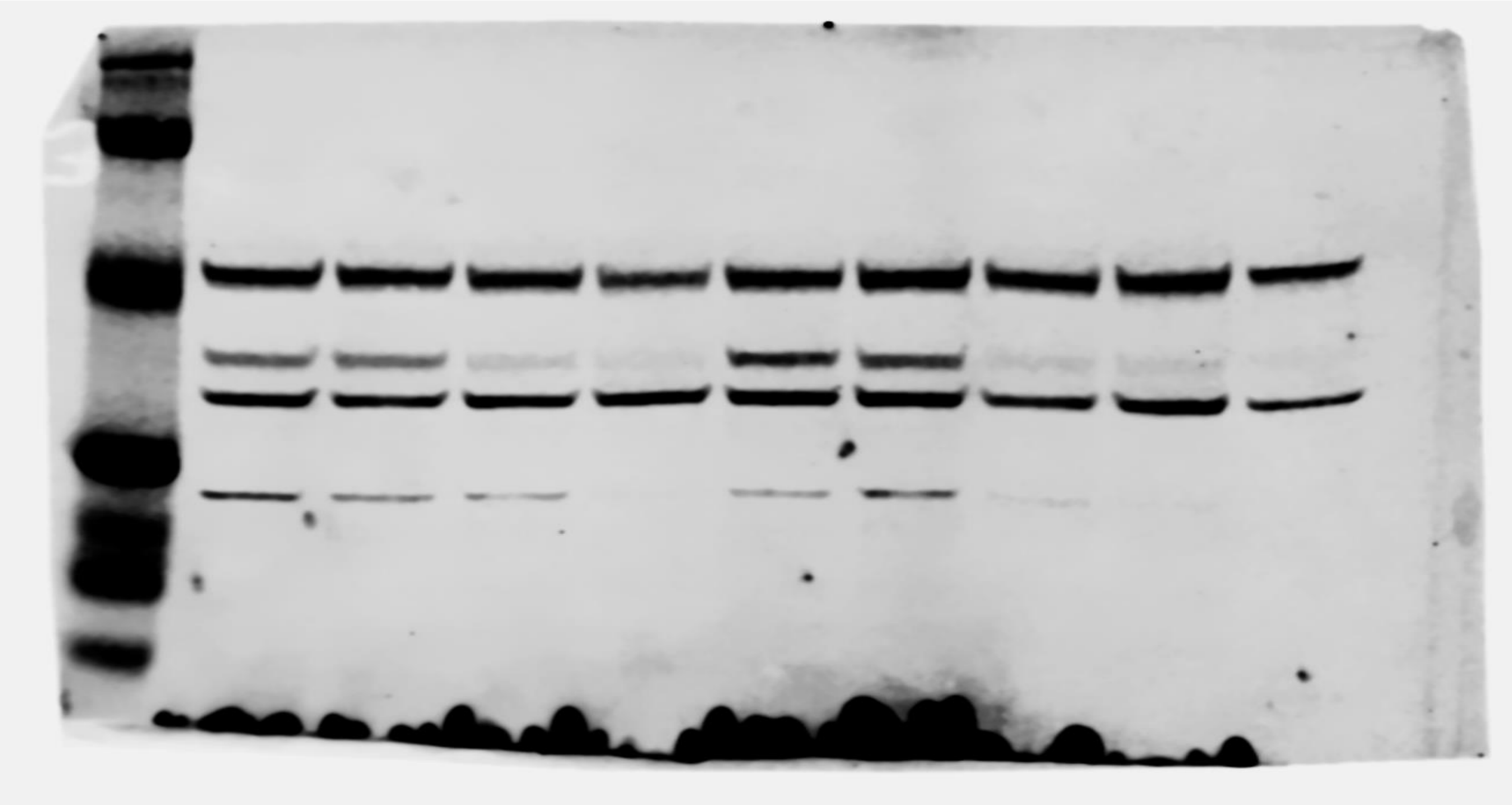

2B

GAPDH

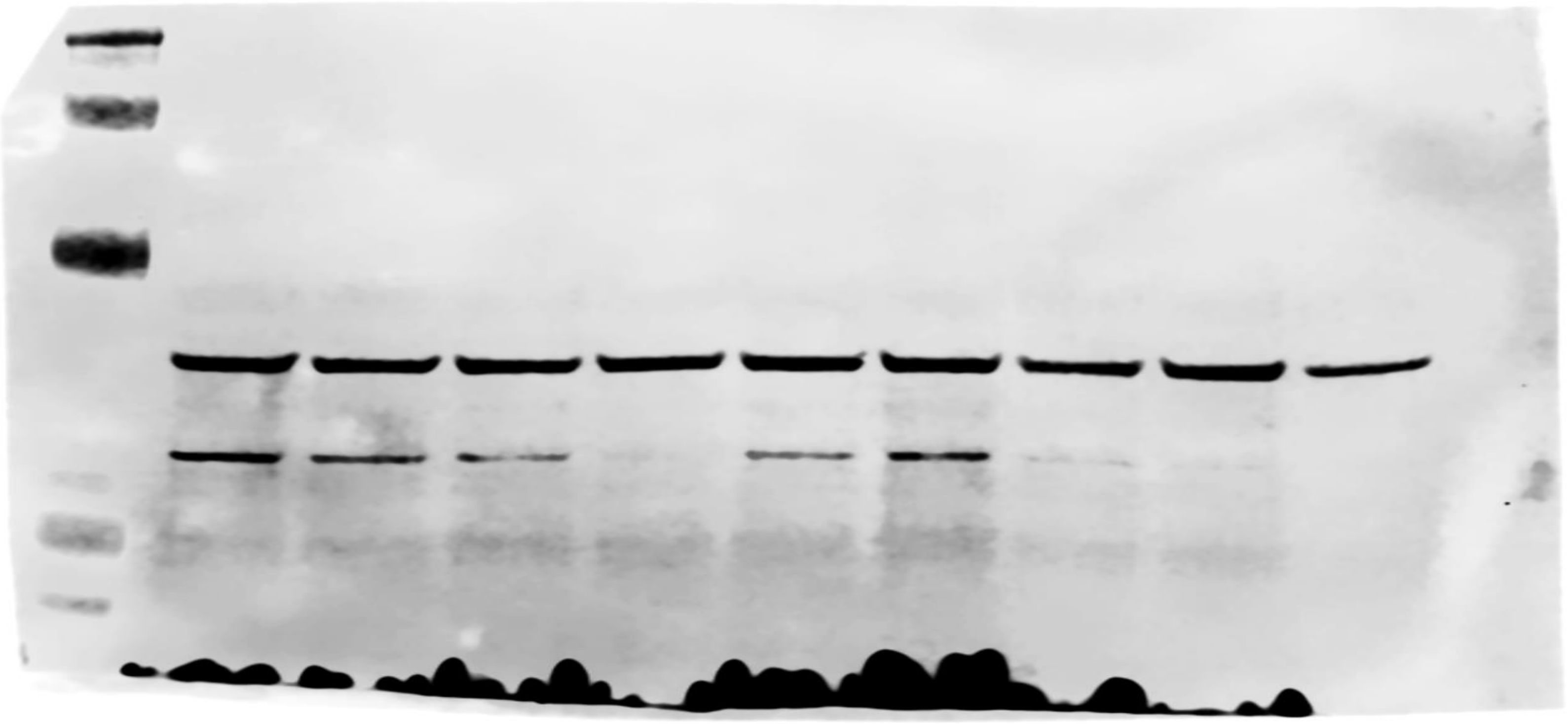

2B

Cyclin D1

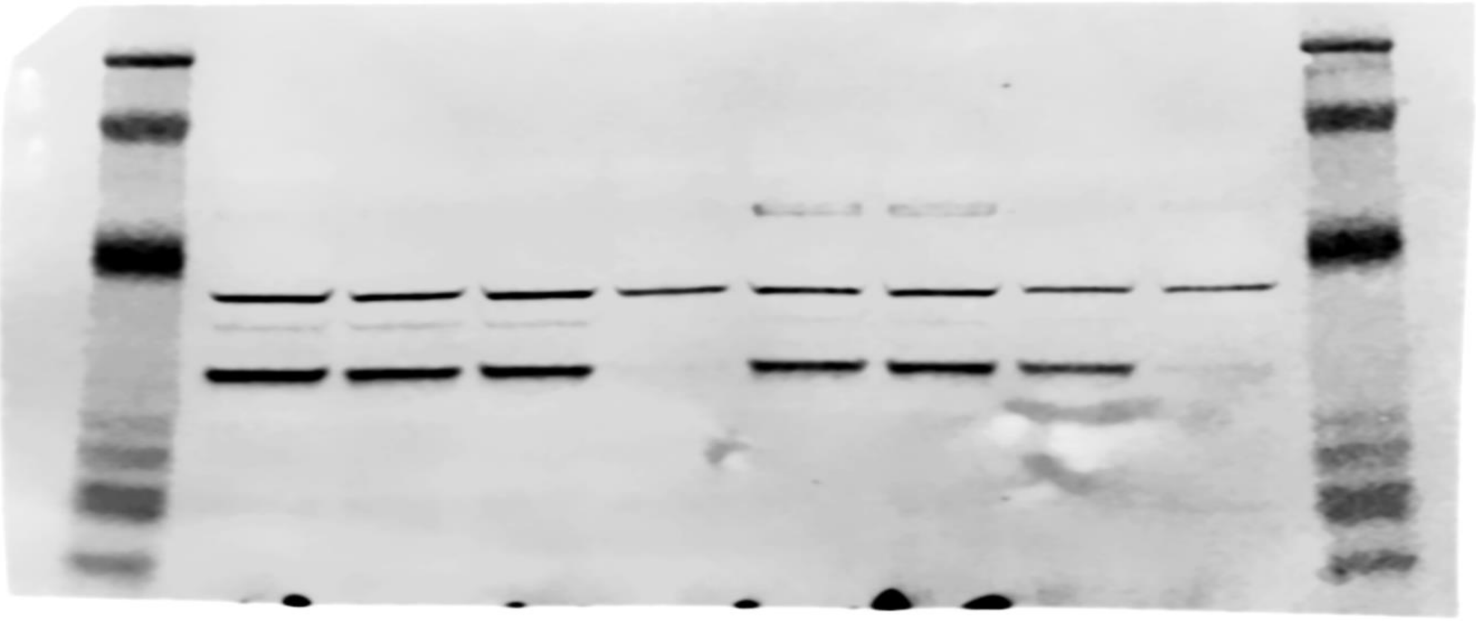

2B

Cyclin E2

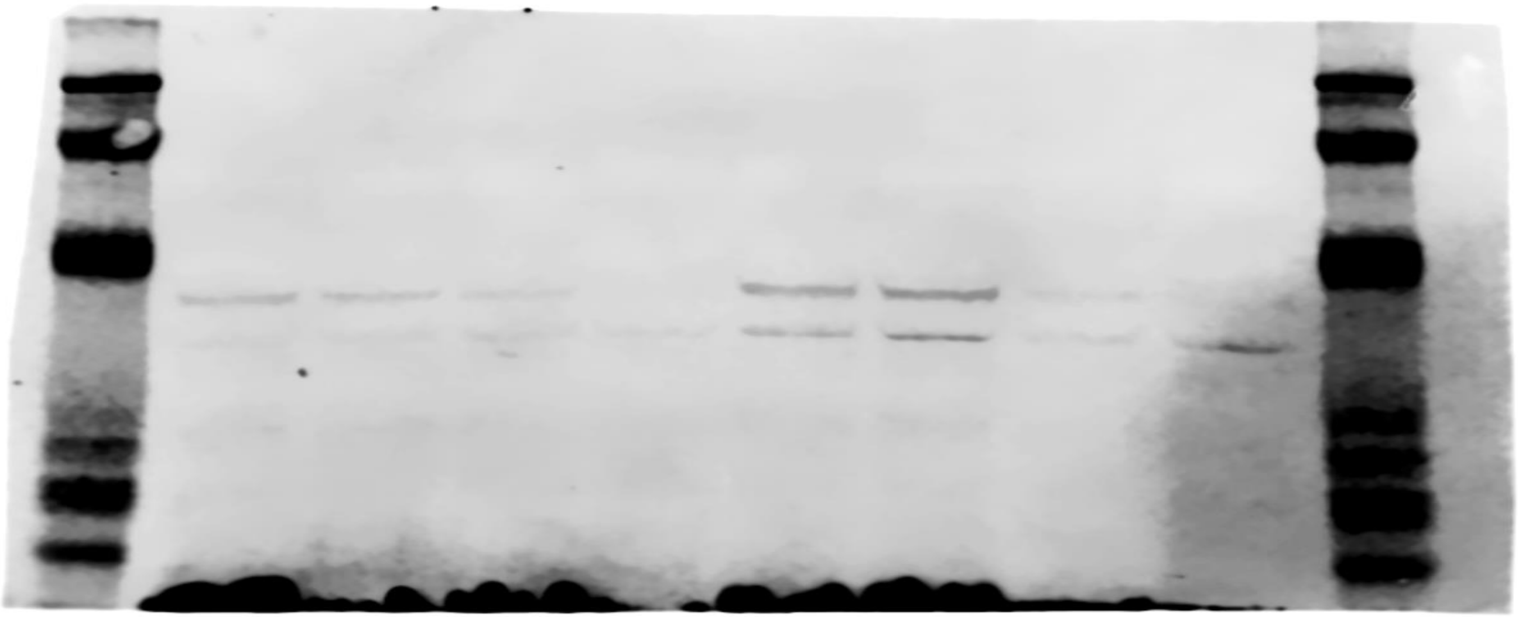

2B

pRb

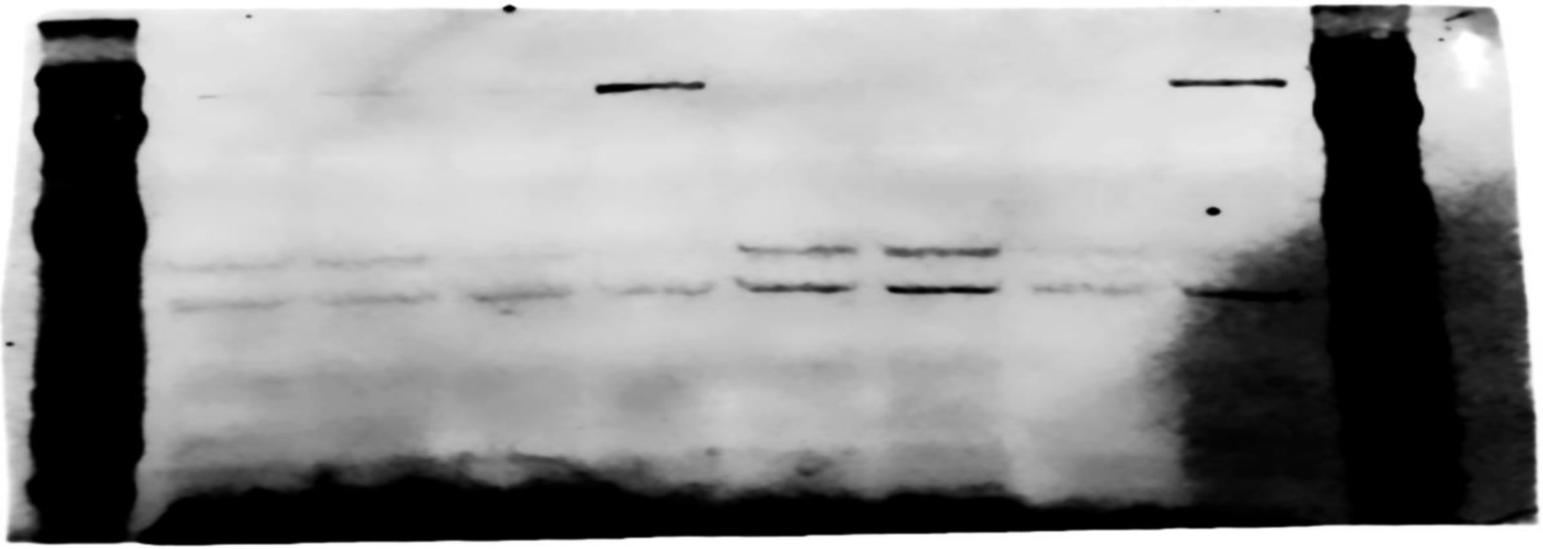

2B

Rb

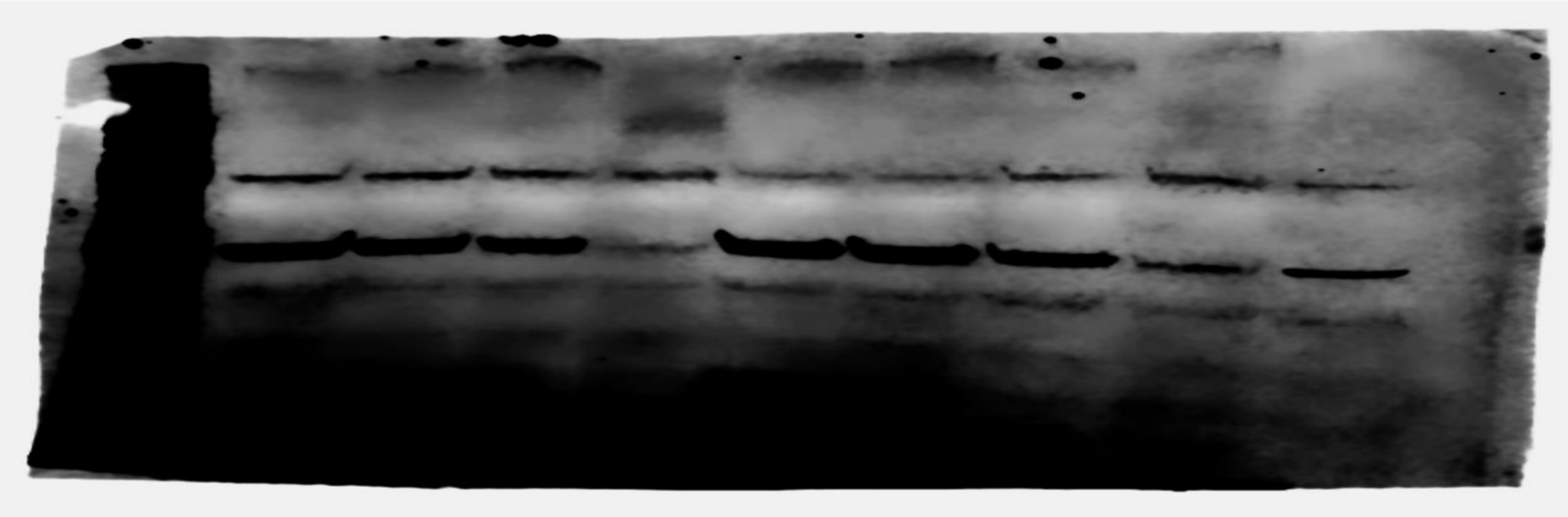

2B

Akt

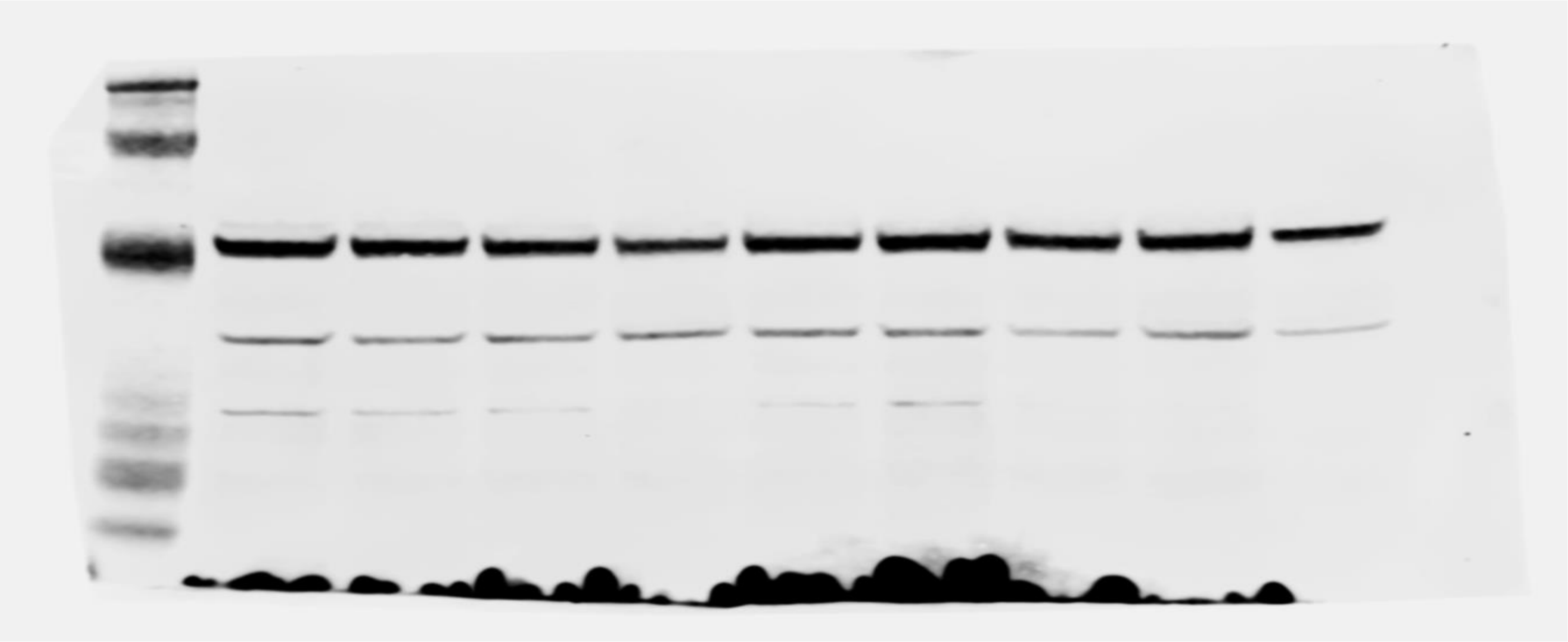

2B

p100

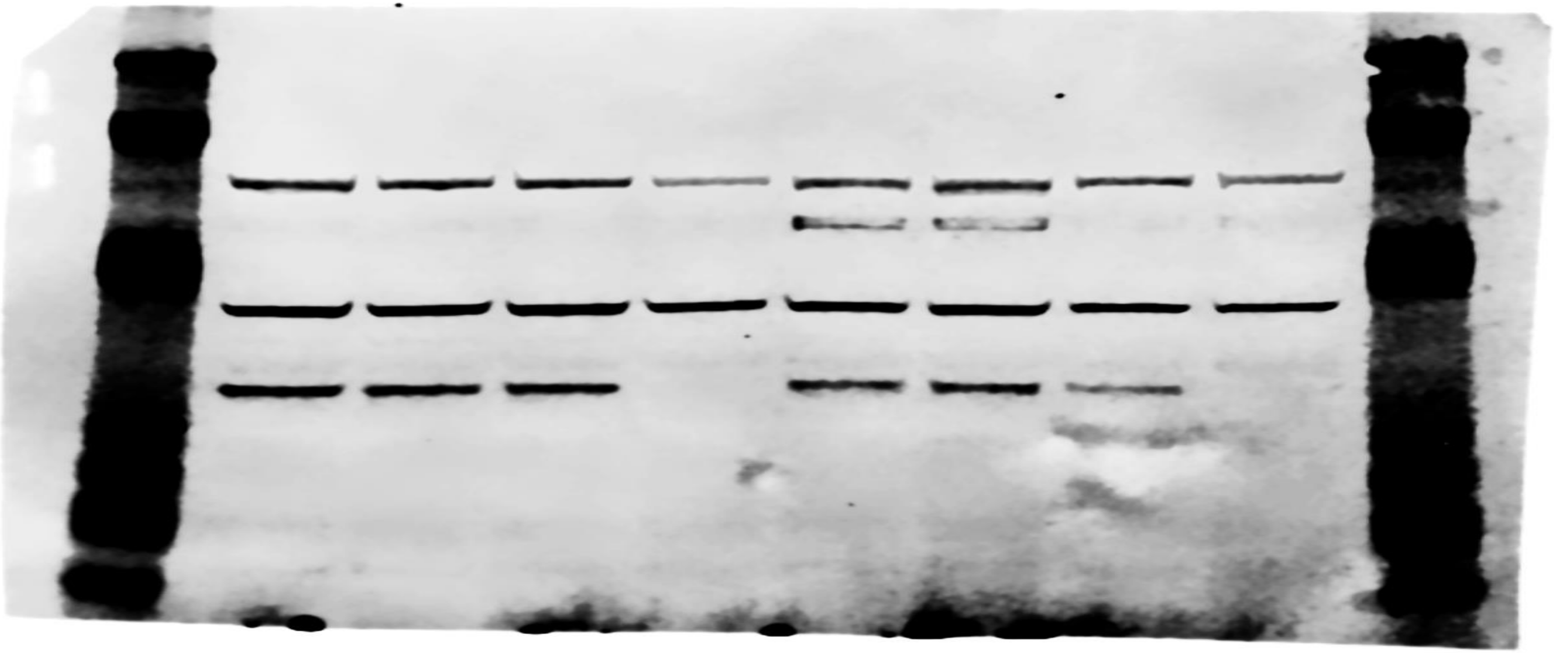

2B

cRel

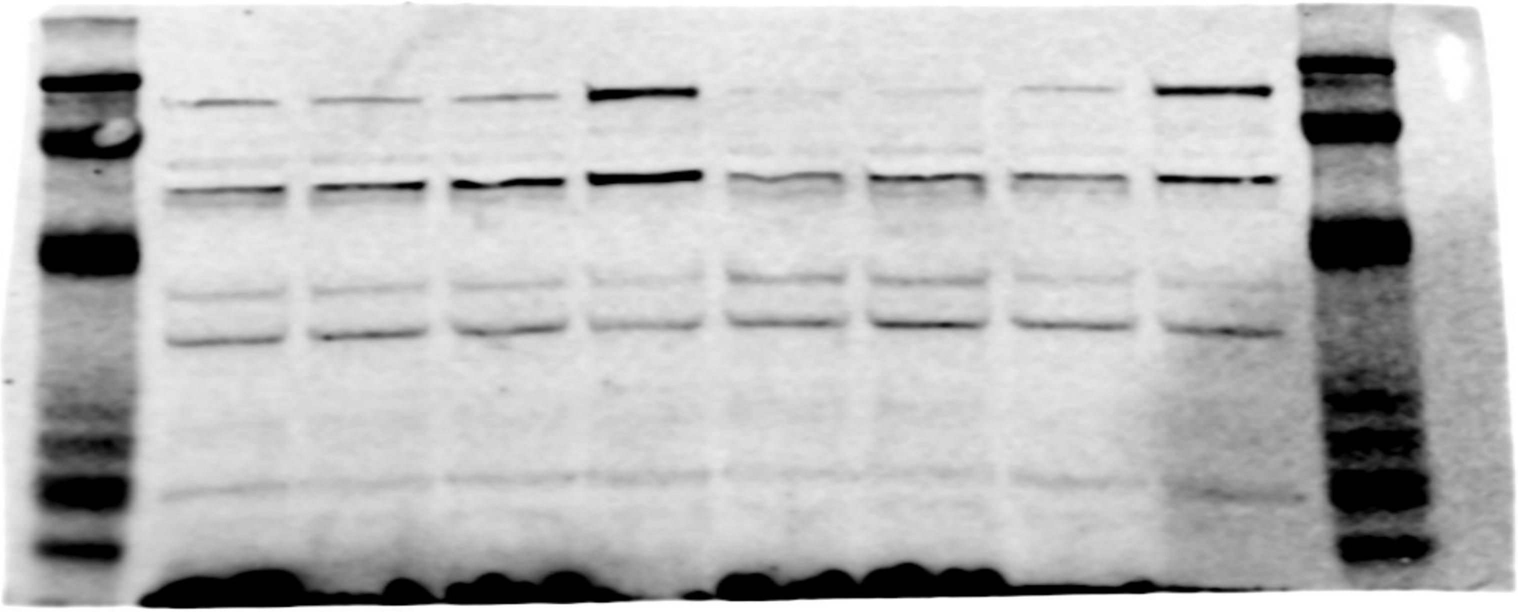

2B

Mut p53

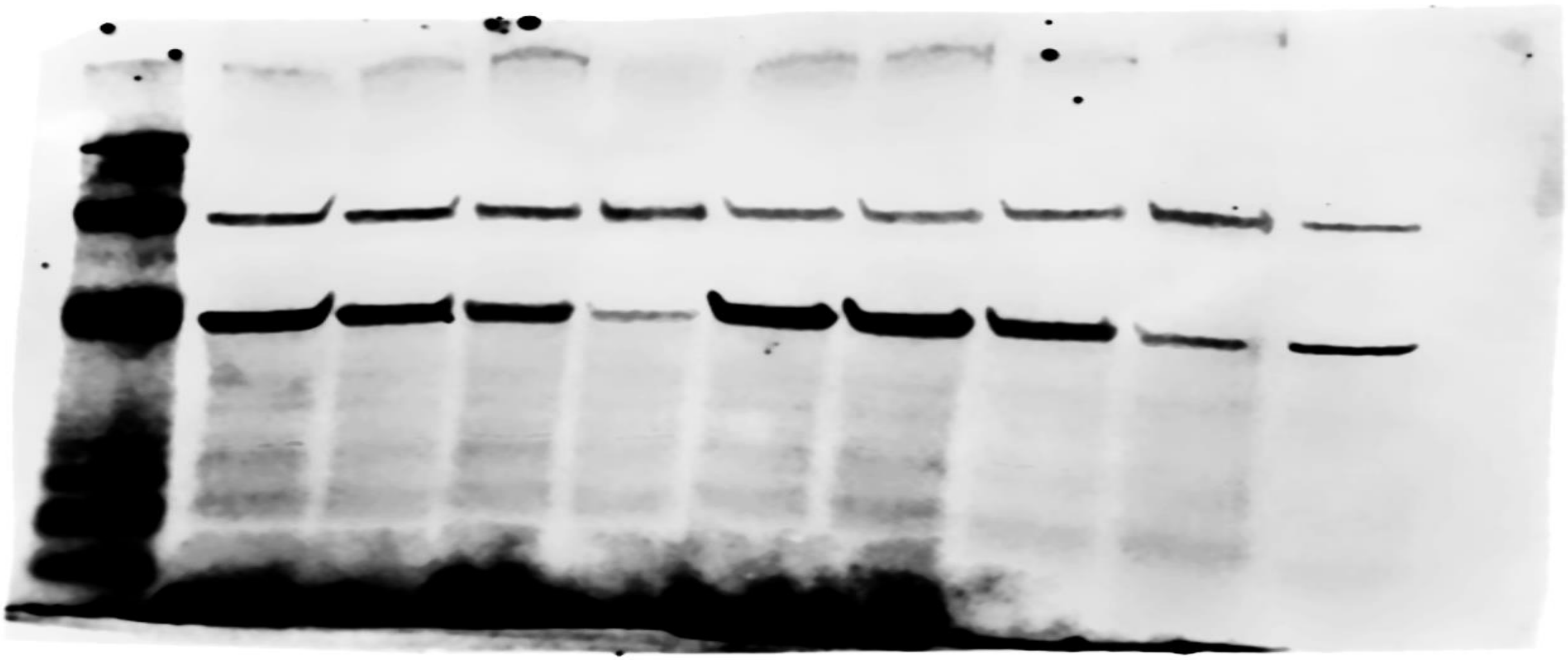

2B

RRM2

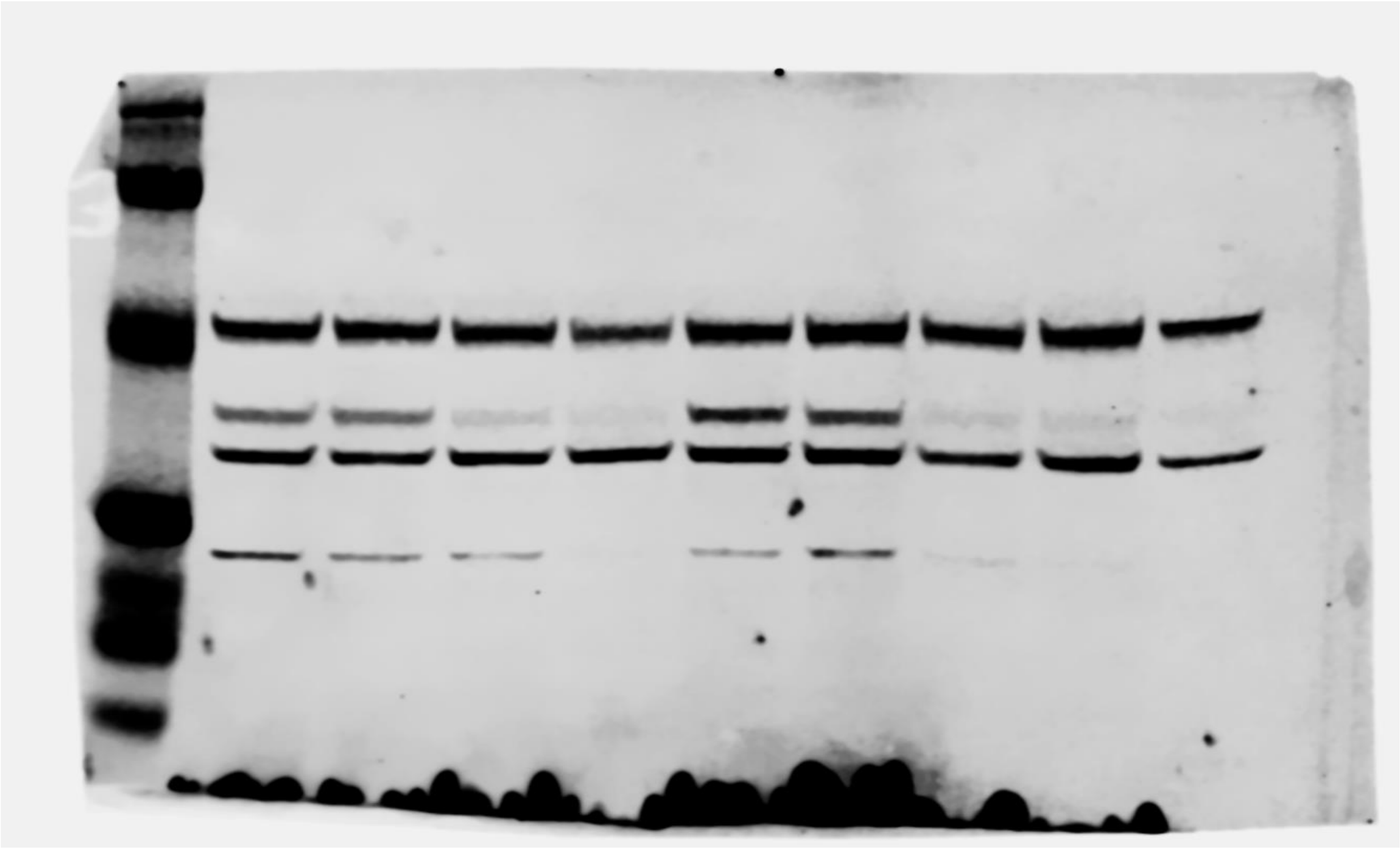

2B

GAPDH

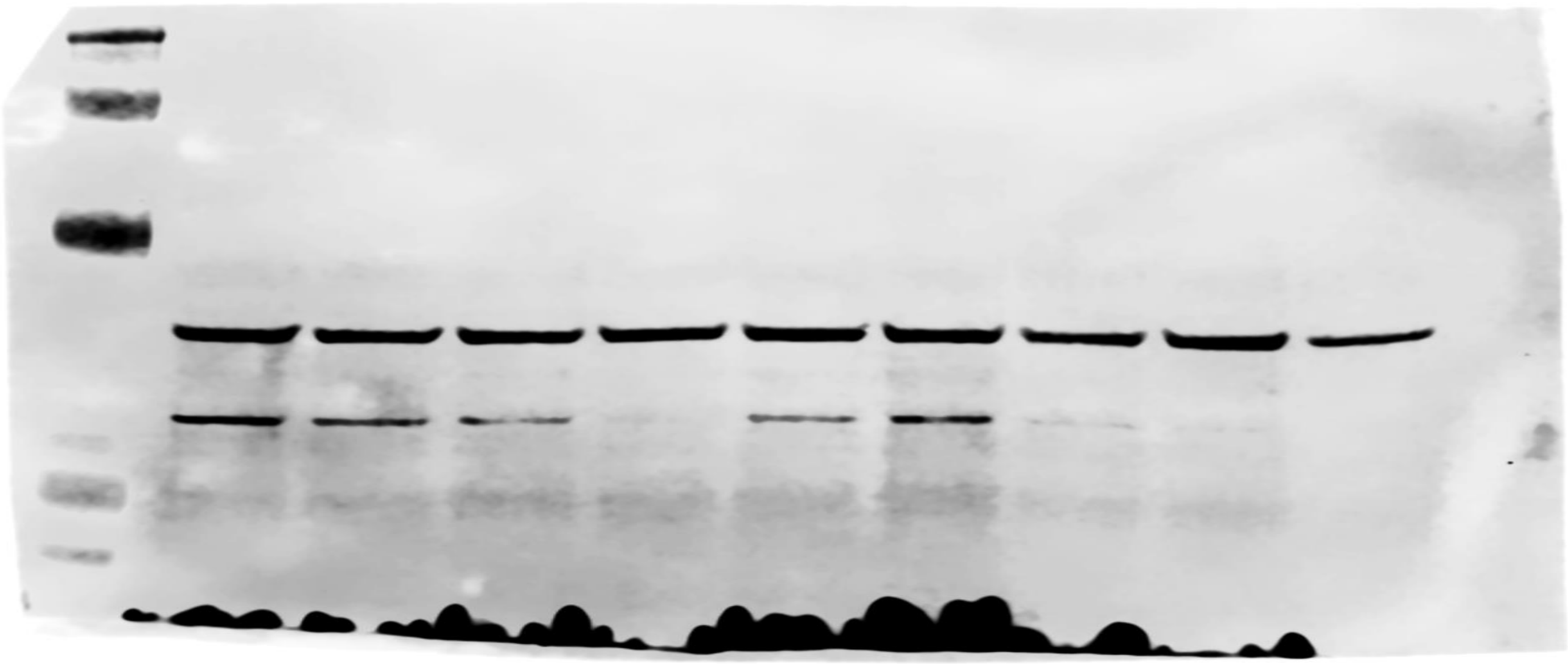

Supplement: Supplementary file 1 [file cancers-16-00975-s001.zip › cancers-2872647-supplementary.pdf]
